# *In vitro* and *in silico* analysis of synthesized *N*-benzyl indole-derived hydrazones as potential anti-triple negative breast cancer agents†

**DOI:** 10.1039/d5ra02194d

**Published:** 2025-04-25

**Authors:** Urva Farooq, Faizullah Khan, Suraj N. Mali, Uzma Ghaffar, Javid Hussain, Ajmal Khan, Somdatta Y. Chaudhari, Hind A. AL-Shwaiman, Abdallah M. Elgorban, Rahul D. Jawarkar, Waseem Ul Islam, Ahmed Al-Harrasi, Zahid Shafiq

**Affiliations:** a Institute of Chemical Sciences, Bahauddin Zakariya University Multan 60800 Pakistan zahidshafiq@bzu.edu.pk; b Department of Pharmacy, Abdul Wali Khan University Mardan KPK Pakistan; c Natural and Medical Sciences Research Centre, University of Nizwa P. O. Box 33, PC 616, Birkat Al Mauz Nizwa Sultanate of Oman aharrasi@unizwa.edu.om; d Department of Pharmaceutical Chemistry, School of Pharmacy, Dr D.Y. Patil Deemed to be University Navi Mumbai India; e Department of Biological Sciences and Chemistry, University of Nizwa Oman; f Department of Chemical and Biological Engineering, College of Engineering, Korea University 145 Anam-ro, Seongbuk-gu Seoul 02841 Republic of Korea; g Department of Pharmaceutical Chemistry, Modern College of Pharmacy Nigdi Pune India; h Department of Botany and Microbiology, College of Science, King Saud University P. O. Box 2455 Riyadh 11451 Saudi Arabia; i Department of Pharmaceutical Chemistry, Dr Rajendra Gode Institute of Pharmacy, University-Mardi Road Ghatkheda Amravati Maharashtra 444602 India; j Department of Pharmaceutical Chemistry, Birla Institute of Technology Mesra India

## Abstract

Triple-negative breast cancer (TNBC) is one of the most aggressive forms of breast cancer, and it is characterized by a high recurrence rate and the rapid development of drug resistance across various subtypes. Currently, there is no targeted therapy, which is specifically approved for the treatment of TNBC. In this study, we synthesized a series of *N*-benzyl indole-3-carboxaldehyde-based hydrazones and subjected them to *in vitro* anticancer studies on MCF-10A and MDA-MB-231 breast cancer (BC) cell lines. Our *in vitro* results suggested that all the compounds exhibited significant anti-TNBC activity, especially on MDA-MB-231 cells. Compound 5b showed excellent activity on MDA-MB-231 (IC_50_ = 17.2 ± 0.4 nM). Furthermore, molecular docking analysis revealed that this compound had a higher binding affinity towards the target EGFR (epidermal growth factor receptor) with a docking score of −10.523 kcal mol^−1^. The molecular dynamics simulation of complex 5b:3W2S showed stable binding over a period of 100 ns. A detailed multi-linear regression (MLR) QSAR denoted the importance of key molecular descriptors, such as com_accminus_2A, fringNlipo6A, and sp^3^Cplus_AbSA. These analyses indicate that the synthesized compounds deserve further studies for developing novel and more potent candidates against triple-negative breast cancer.

## Abstract 2

Triple-negative breast cancer (TNBC) is one of the most aggressive forms of breast cancer, and it is characterized by a high recurrence rate and the rapid development of drug resistance across various subtypes.

## Graphical abstract

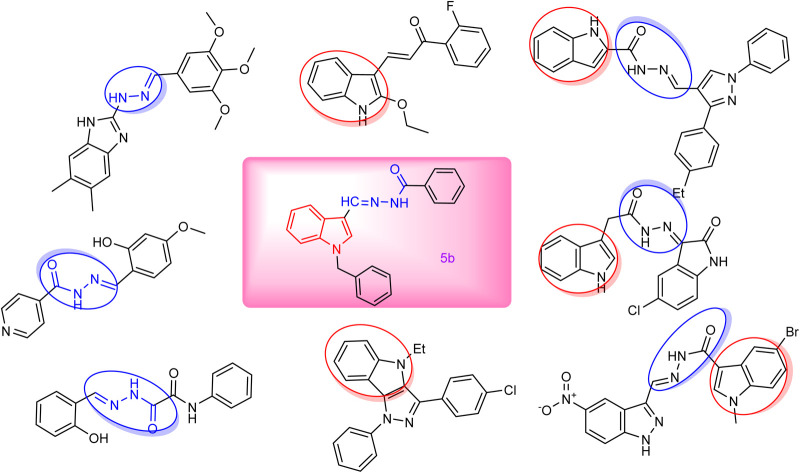

## 1. Introduction

Cancer is a major obstacle to increasing life expectancy and is one of the leading causes of death worldwide.^1–3^ Approximately 1 million cases of breast cancer are diagnosed globally each year, with over 170 000 classified as triple-negative breast cancer (TNBC).^4^ TNBC lacks estrogen and progesterone receptors and exhibits overexpression of HER2, and it overlaps with basal-like breast cancer.^5–10^ This subtype is associated with poor prognosis owing to its high recurrence rate, limited progression-free survival, and the absence of targeted therapies. However, not all TNBC cases have a poor outcome.^11,12^ Although chemotherapy remains the primary treatment, emerging agents, such as PARP inhibitors, show promise and are currently undergoing clinical trials.^12,13^

When the epidermal growth factor receptor (EGFR) is significantly overexpressed, it exhibits distinctive genetic characteristics compared with other tumor types, and treatments, including immunotherapy and chemotherapy, are available for metastatic TNBC.^14^ However, owing to the lack of estrogen receptor (ER) expression, patients with ER-negative tumors have an inadequate response to hormone treatments, making treatment more difficult.^15^ It has also been noted that some polyphenols have the ability to convert ER-negative tumors to ER-positive ones, enhancing prognosis and enabling treatment with selective estrogen receptor modulators (SERMs), such as tamoxifen.^16^ ER stimulation enhances susceptibility to tamoxifen in the ER-BC cell line MDA-MB-231.^17^

**Fig. 1:**
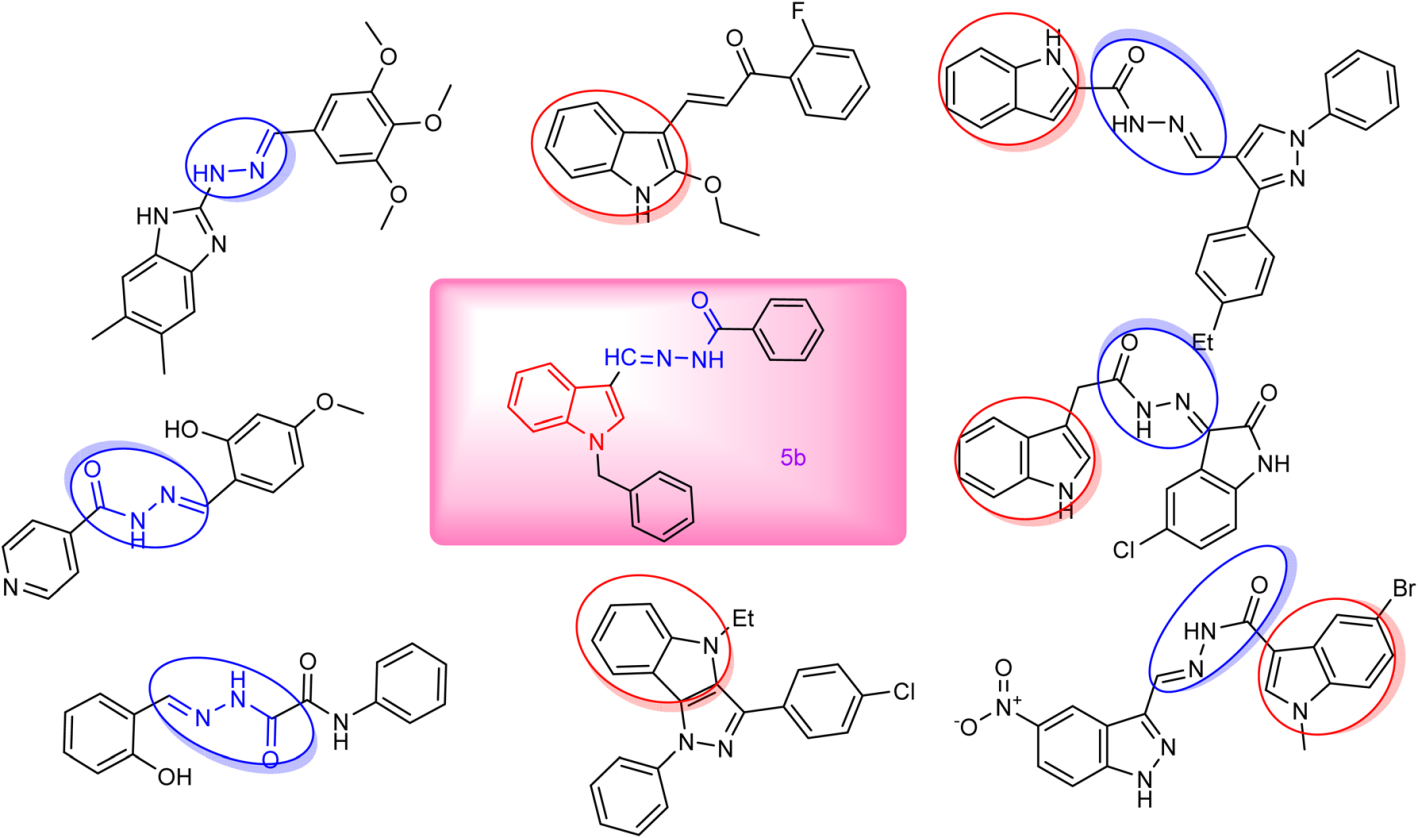
Reported hydrazones as anti-breast cancer (BC) agents against the MDA-MB-231 cell line.

The hydrazone framework provides a distinct structure for a well-defined pharmacophore, making it an important component in medicinal chemistry (Fig. 1). Acting either as a hydrogen bond acceptor or donor, this structure can interact with several amino acids of biological relevance.^18^ Apart from their various pharmacological qualities, including analgesic,^19^ antitubercular,^20^ and antibacterial,^21^ hydrazone derivatives are often vital for the development of anticancer drugs.^22–24^ Heterocycles have exhibited a noteworthy part in anticancer drug discovery and are considered vital pharmacophores for the development of new leading compounds.^25–27^ Nitrogen-based heterocycles represent a valuable source of therapeutically active compounds and play a crucial role in the structural design of anticancer drugs.^28^ Indole is a crucial scaffold, and its derivatives have demonstrated significant effectiveness in inducing cell death across various cancer cell lines.^29,30^ This includes their ability to induce oxidative stress and cell death, along with the suppression of DNA repair, tumor blood vessel formation, normal cell cycle progression, and cell signaling. Indole has served as a highly valued framework for the development and enhancement of anticancer drugs owing to its adaptability.^31,32^ Herein, we synthesized a set of newer *N*-benzyl indole-derived hydrazones and tested them *in vitro* for their anti-TNBC activity. Furthermore, molecular modeling studies, such as molecular docking, and dynamics, were conducted to check the stable interaction of synthesized compounds towards the target involved in the progression of TNBC. A GA-MLR-based QSAR analysis also revealed the importance of key molecular descriptors for developing potent analogues against TNBC.

## 2. Results and discussion

### 2.1. Chemistry

**Scheme 1:**
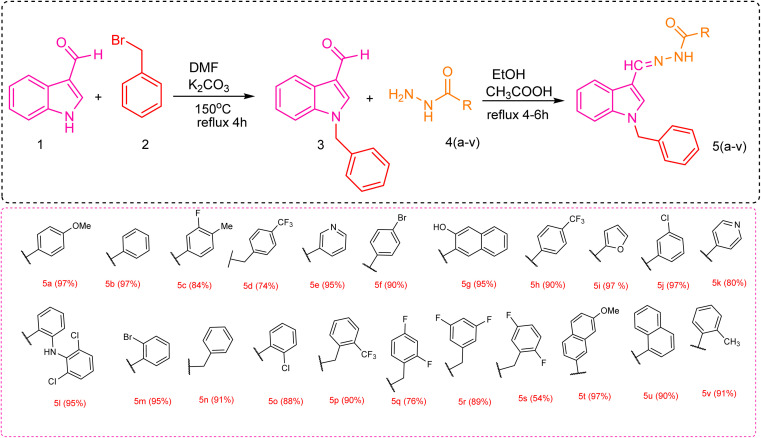
Synthetic route for the synthesis of *N*-benzyl indole-based hydrazones.

Scheme 1 describes the synthetic route for the synthesis of novel compounds 5a–v. Two primary procedures were followed in synthesizing hydrazones 5a–v depending on 1-benzyl-1*H*-indole-3-carboxaldehyde. First, 1-benzyl-1*H*-indole-3-carboxaldehyde (3) was synthesized from indole-3-carboxaldehyde. This was achieved by refluxing indole-3-carboxaldehyde (1) with benzyl bromide (2) in methanol (MeOH) using K_2_CO_3_ as a catalyst, which facilitated the *N*-benzylation of the compound. In the second step, a series of 1-benzyl-1*H*-indole-3-carboxaldehyde-based hydrazones 5a–v was synthesized. An equimolar ratio of benzyl-1*H*-indole-3-carboxaldehyde (3) and respective hydrazide 4a–v was refluxed in MeOH for the synthesis of desired hydrazones 5a–v, and acetic acid (CH_3_COOH) was utilized as a catalyst.

Various characterization techniques, such as FT-IR (Fourier Transform Infrared Spectroscopy), ^1^H-NMR, ^13^C-NMR spectroscopies and high-resolution mass spectrometry (HRMS), were employed to confirm the structures of the new compounds 5a–v. In FTIR spectroscopy, the absorption of –NH bands in the hydrazide moiety was shown in a region of 3500–3300 cm^−1^. The aromatic absorption was observed in 600–900 cm^−1^ region.

In ^1^H-NMR, a singlet was observed in the 10.18–11.86 ppm region, which was assigned to the proton of the hydrazide's NH group. An additional singlet was observed in compounds 5l–v in the 11.28–11.74 ppm region, which can be assigned to the iminol group, indicating the presence of tautomerism phenomena in these synthesized compounds. The protons of –CH_2_ of the benzyl group appeared in the 5.43–5.56 ppm region. The aromatic protons all appeared in the 7–8 ppm range.

In the ^13^C-NMR spectroscopy, for compounds 5l–v, peak data demonstrated an increase in carbon atoms in comparison to the targeted synthesized compound. Two peaks (at 151 MHz) appeared in the carbonyl region at 160–210 ppm, indicating the presence of tautomerism.

In the HRMS spectra, molecular ion peaks denoted as [M + H]^+^ are precisely aligned with the molecular weight of the synthesized compounds.

### 2.2. Biological activity

All synthesized compounds (5a–5v) were tested using MTT assay at various concentrations (6.5 μM, 12.5 μM, 25 μM, and 50 μM) on the human breast cancer cell line MDA-MB-231. The cytotoxicity of the synthesized analogues was also assessed using human normal breast epithelial cell lines MCF-10A. Table 1 denotes IC_50_ values, percent inhibition, and cell viability for compounds 5a–v on MDA-MB-231. The dose–response analysis and calculations of IC_50_ values were performed using IBM SPSS statistics 26. The MTT assay on MDA-MB-231 confirmed the anticancer potential of all compounds. In particular, with an IC_50_ value of 17.2 ± 0.4 μM, compound 5b demonstrated the maximum activity. We then followed the same protocol as the cancer cells for the non-tumorigenic MCF-10A cells but with different concentrations (6.5 μM, 12.5 μM, 25 μM, and 50 μM). This was done to determine whether the toxic effects of the chemicals were selective for cancer cells compared to non-cancerous cells. From Table 1, it was clear that all compounds exhibited higher IC_50_ values (>50) against MCF-10A cells, indicating their lesser toxicity on normal cells.

**Table 1:** Percentage inhibitions and IC_50_ values for newly synthesized compounds (5a–5v) on the TNBC cell line (MDA-MB-231) and MCF-10A

Comp. ID.	Breast cancer cell line (MDA-MB-231)		Normal cell line (MCF-10A)	
	Viability (%)	IC_50_ values (μM)	Viability (%)	IC_50_ values (μM)
5a	32.45	25.6 ± 0.4	82.65	>50
5b	18.56	17.2 ± 0.4	77.09	>50
5c	37.40	27.1 ± 0.4	85.69	>50
5d	27.94	22.6 ± 0.8	80.19	>50
5e	31.47	28.2 ± 0.5	85.74	>50
5f	25.05	21.8 ± 0.2	82.43	>50
5g	29.76	24.4 ± 0.6	81.83	>50
5h	33.07	30.8 ± 0.4	78.08	>50
5i	23.53	19.2 ± 0.4	84.63	>50
5j	21.87	22.6 ± 0.1	80.79	>50
5k	23.44	21.4 ± 0.2	80.07	>50
5l	37.21	28.1 ± 0.4	79.88	>50
5m	29.14	26.4 ± 0.2	87.24	>50
5n	44.16	43.4 ± 0.2	79.77	>50
5o	20.37	19.6 ± 0.5	82.33	>50
5p	25.67	24.6 ± 0.1	83.09	>50
5q	41.09	37.6 ± 0.2	79.53	>50
5r	36.18	31.6 ± 0.1	84.07	>50
5s	38.14	29.4 ± 0.8	86.28	>50
5t	24.47	20.2 ± 0.4	80.87	>50
5u	30.59	26.6 ± 0.1	83.59	>50
5v	28.11	23.1 ± 0.6	81.04	>50

#### 2.2.1. Structure–activity relationship

**Fig. 2:**
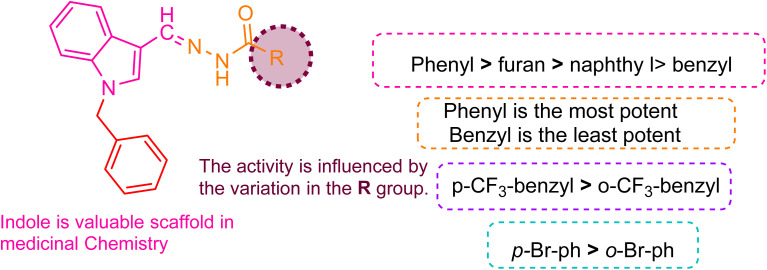
Pictorial representation of the structure–activity relationship.

The SAR is mainly affected by the R group linked with the hydrazide moiety. In this study, we used various aromatic rings, including pyridyl, naphthyl, phenyl, and benzyl, positioned differently and combined with various substituents (Fig. 2). Compound 5b with phenyl substitution exhibited good effectiveness with an IC_50_ value of 17.2 ± 0.4 μM. In contrast, compound 5n with the benzyl group and hydrazide moiety showed a steep decline in inhibitory potential and was found to be the least potent molecule with an IC_50_ of 43.4 ± 0.2 μM.

The ranking order of bicyclic and heterocyclic substituents is 5i (furan, IC_50_ = 17.2 ± 0.4 μM) > 5t (2-methoxy naphthoic, IC_50_ = 20.2 ± 0.4 μM) > 5k (iso-nicotinic, IC_50_ = 21.4 ± 0.2 μM) > 5g (3-hydroxy-2-naphthoic, IC_50_ = 24.4 ± 0.6 μM) > 5u (naphthyl, IC_50_ = 26.6 ± 0.1 μM) > 5e (nicotinic, IC_50_ = 28.2 ± 0.5 μM). Comparing the potency of different rings attached to the hydrazide moiety, compound 5k with the pyridyl group attached at the para position with the hydrazide moiety showed notable activity with an IC_50_ of 21.4 ± 0.2 μM compared to compound 5e with the pyridyl group attached at the meta position (an IC_50_ = 28.2 ± 0.5 μM). This indicates that para linking was more favorable than meta linking. Compound 5i with furan substitution attached at 2 positions with hydrazide moiety showed good potency with an IC_50_ of 17.2 ± 0.4 μM and was recorded as the second most potent compound from the series. Compound 5t with 2-mathoxynaphthoic substitution (IC_50_ = 20.2 ± 0.4 μM) showed outstanding potential, and compound 5g with a 3-hydroxy-2-naphthoic group (IC_50_ = 24.4 ± 0.6 μM) also exhibited good inhibitory potential compared to compound 5u with naphthyl substitution (IC_50_ = 26.6 ± 0.1 μM). This showed that linking the electron donating group with the naphthyl ring increased its potency. As the methoxy group attached to naphthyl is more electron-donating (+I) than the hydroxy group, we found that it had a direct impact on biological activity.

The trend of mono-substitution was 5o (2-chlorophenyl, IC_50_ = 19.6 ± 0.5 μM) > 5f (4-bromophenyl, IC_50_ = 21.8 ± 0.2 μM) > 5d (4-(trifluoromethyl)benzyl, IC_50_ = 22.6 ± 0.8 μM) > 5j (3-chlorophenyl, IC_50_ = 22.6 ± 0.1 μM) > 5v (2-methylphenyl, IC_50_ = 23.1 ± 0.6 μM) > 5p (2-(trifluoromethyl)benzyl, IC_50_ = 24.6 ± 0.1 μM) > 5a (4-methoxyphenyl, IC_50_ = 25.6 ± 0.4 μM) > 5m (2-bromophenyl, IC_50_ = 26.4 ± 0.2 μM) > 5h (4-(trifluoromethyl)phenyl, IC_50_ = 30.8 ± 0.4 μM). When comparing derivatives with mono-substitutions, compound 5f with 4-bromophenyl substitution had an IC_50_ value of 21.8 ± 0.2 μM, denoting promising inhibitory potential compared to compound 5m (with 2-bromophenyl group; IC_50_ of 26.4 ± 0.1 μM). Compound 5j with the 4-(trifluoromethyl)benzyl group demonstrated better inhibitions than compound 5p with the 2-(trifluoromethyl)benzyl group. Compound 5a with the 4-methoxyphenyl group displayed more inhibition than compound 5h with the trifluoromethyl group at the same position. This is due to the +I of the methoxy group, which is more favorable in inhibiting cancer cells than the −I of the trifluoromethyl group. Compound 5j with 3-chlorophenyl substituent linked with hydrazide moiety exhibited less potential with an IC_50_ of 22.6 ± 0.1 μM than compound 5o with 2-chlorophenyl substitution (IC_50_ = 19.6 ± 0.5 μM). This demonstrated that ortho linking was more significant for designing such derivatives. Compound 5v with 2-methylphenyl substitution exhibited reasonable effectiveness with an IC_50_ of 29.4 ± 0.8 μM.

The order of compounds with di-substitution was 5c (3-flouro-4-methylphenyl, IC_50_ = 27.1 ± 0.4 μM) > 5l (2-(2,6-dichloro phenyl)amino)benzyl, IC_50_ = 22.6 ± 0.8 μM) > 5s (2,5-diflourobenzyl, IC_50_ = 29.4 ± 0.8 μM) > 5r (3,5-diflourobenzyl, IC_50_ = 31.6 ± 0.1 μM) > 5q (2,4-diflourophenyl, IC_50_ = 37.6 ± 0.2 μM). Compound 5l with the 2,6-dichlorophenyl group displayed significant potency compared to compounds 5s, 5r, and 5q with fluoro substitutions at the (2,5), (3,5), and (2,4) positions of the benzyl ring, respectively. This indicated that chloro substitution being more electron rich was more favorable than fluoro substitution.

### 2.3. Molecular docking analysis

**Fig. 3:**
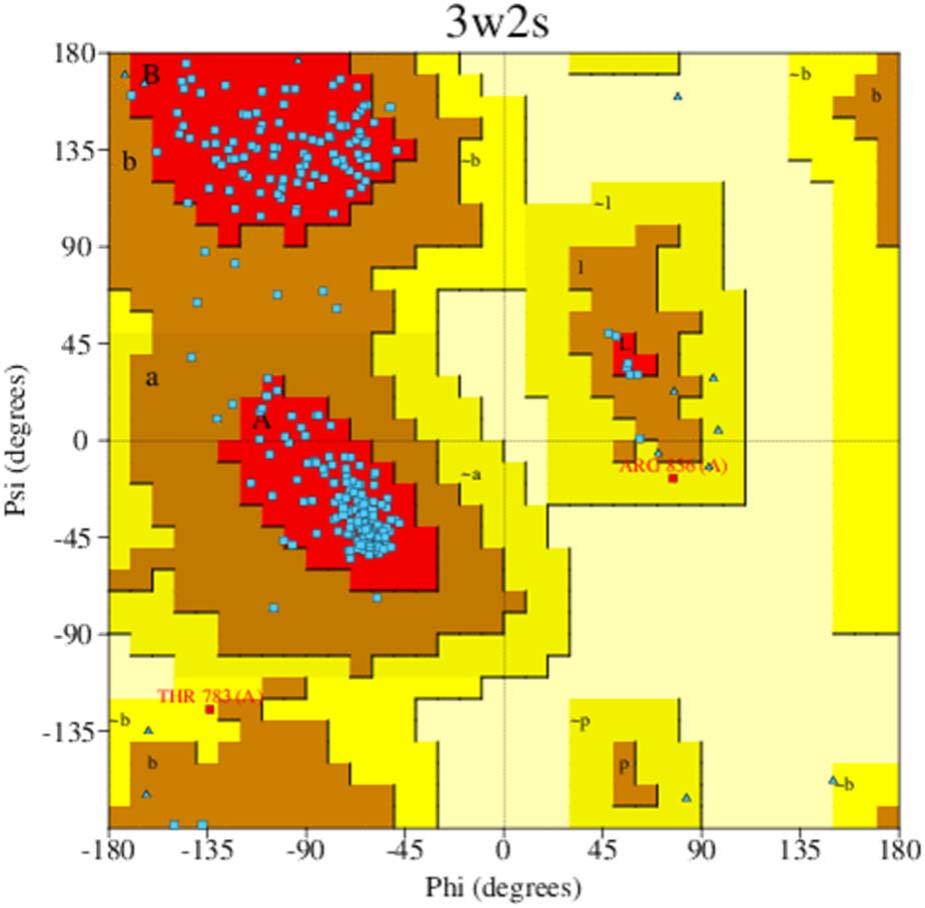
Ramachandran plot for 3W2S obtained from the PROCHECK server.

To study the possible binding mechanisms of the synthesized compounds, we used a molecular docking simulation. The docking simulation was carried out against the selected target, protein EGFR (PDB ID 3W2S), a non-mutated form. The Ramachandran plot analysis *via* the ‘PROCHECK’ server showed 93.8% of residues in favored and allowed regions, underscoring the protein's structural integrity for current studies (Fig. 3).

Multiple essential amino acids within the active site, such as VAL726A, ALA743A, LYS745A, MET766A, LEU788A, THR790A, GLN791A, MET793A, ARG841A, ASN842A, LEU844A, THR854A, ASP855A, PHE856A, GLY857A, and LEU862A, were found to play specific roles in the substrate binding and catalytic activity of the enzyme.

**Fig. 4:**
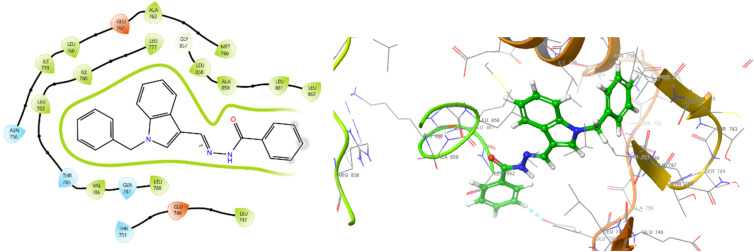
2D and 3D interaction diagrams for 5b with protein 3W2S.

**Fig. 5:**
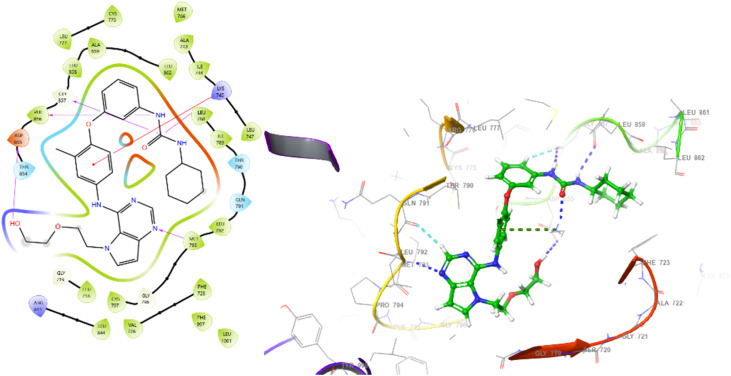
2D and 3D interaction diagrams for the compound W2R (native co-crystal ligand) with protein 3W2S.

The effective binding of ligands to target protein 3W2S can be understood through the functional group characteristics of ligands, such as electron donation or withdrawal capabilities, which determine how they can strongly interact with amino acids located within the active site (Fig. 4 and 5). Aromatic ring electrons become denser when exposed to EDGs, such as –OH and –NH_2_ groups, which enable stronger π-stacking with tyrosine (TYR) and phenylalanine (PHE) present in the binding domain. Amino acid residue PHE723A received stronger π-π interactions from ligand groups that contained these functional groups, thus improving binding affinity. EDGs function through two mechanisms: they enhance amino acid hydrogen bonds with lysine (LYS) and aspartate (ASP) by increasing polarity and donating electrons, which build hydrogen bonds. The presence of electron-withdrawing groups, such as –NO_2_ and –CF_3_, creates positive charges in aromatic systems owing to the removal of electron density, thus creating improved p-cation interactions with cationic residues. Such chemical groups adjust ligand amino acid interactions by raising the ligand's positive character to establish electrostatic bonds with positively charged amino acid residues, including LYS745A. In ligand design, these functional groups must be strategically placed to optimize interactions with specific residues found in the protein active site, thereby acting as better inhibitors. From the overall observation, it has been concluded that compound 5b showed exceptional potential as the best therapeutic agent because its docking results revealed stable interactions comparatively at lesser distances and through its −10.523 kcal mol^−1^ binding energy (lowest IC_50_ value of 17.2 ± 0.4 μM).

### 2.4. Binding free energy analysis

For the calculation of binding free energies of protein–ligand complexes, we used ‘Molecular Mechanics combined with the Generalized Born Surface Area (MMGBSA) method (computed using the Python script thermalmmgbsa.py using the last 50 frames of simulation trajectories with each step sampling size). The MMGBSA (kcal mol^−1^) analysis was estimated by adding various energy modules, such as covalent, coulombic, Vander Waal, and lipophilic solvation, which were collectively considered.

The following equation is used to calculate Δ*G*_bind_:Δ*G*_bind_ = Δ*G*_MM_ + Δ*G*_solv_ − Δ*G*_SA_,where Δ*G*_bind_ refers to the binding free energy, Δ*G*_MM_ represents the difference between the free energies of ligand–protein complexes and the total energies of the isolated protein and ligand,

Δ*G*_Solv_ indicates the difference in the GSA solvation energies of the ligand–receptor complex compared to the sum of the solvation energies of the receptor and ligand when they are unbound, and Δ*G*_SA_ represents the difference in the surface area energies for both the protein and the ligand.

### 2.5. Molecular dynamics simulation

**Fig. 6:**
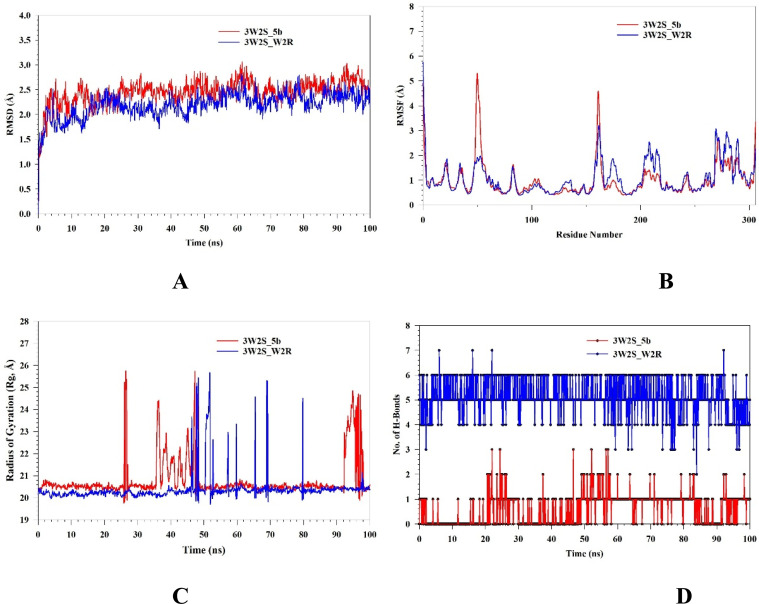
MD simulation analysis of 100 ns trajectories of (A) RMSD of Cα backbone of 3W2S_5b (red line) and 3W2S_W2R (blue line). (B) RMSF of Cα backbone of 3W2S_5b (red line) and 3W2S_W2R (blue line). (C) Radius of gyration (*R*_g_) of Cα backbone of 3W2S_5b (red line) and 3W2S_W2R (blue line), and (D) formation of hydrogen bonds in 3W2S_5b (red line) and 3W2S_W2R (blue line).

**Fig. 7:**
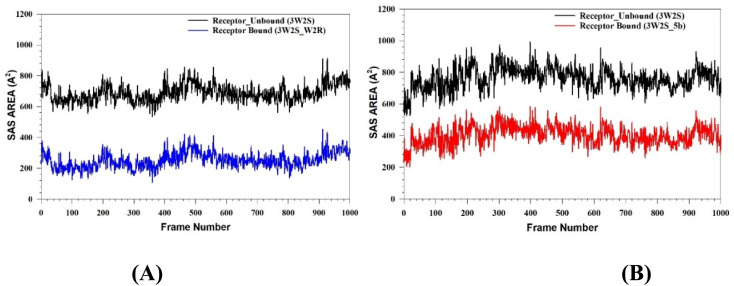
MD simulation analysis of 1000 frame works of (A) solvent accessible surface area of 3W2S_5b and (B) solvent accessible surface area of 3W2S_W2R.

Molecular dynamics (MD) studies were conducted to determine the stability and convergence of complexes [3W2S with 5b and W2R represented] as 3W2S_5b and 3W2S_W2R, respectively. Fig. 6A illustrates a Root Mean Square Deviation (RMSD) analysis providing insight into the structural stability of two systems (3W2S_5b and 3W2S_W2R) over a simulation time of 100 ns. Both systems exhibited an initial rise in RMSD before stabilization. The 3W2S_5b demonstrated slightly higher fluctuations with RMSD averaging up to 2.45 Å compared to 3W2S_W2R. With an average RMSD of 2.20 Å, this suggests that the 3W2S_W2R maintained a relatively more stable conformation. Fig. 6B depicts the Root Mean Square Fluctuation (RMSF) analysis over a simulation period of 100 ns. Both structures exhibited similar fluctuation patterns, with notable peaks around specific residues: 0, 49–52, 161–162, and 306. These peaks correspond to highly flexible loop regions. The higher fluctuations in certain residues of 3W2S_5b, with an RMSF value of 1 Å, compared to the average RMSF of 1.06 Å in 3W2S_W2R, suggested that the 3W2S_5b exhibited lower flexibility. Fig. 6C depicts the radius of gyration (*R*_g_) analyses. It was used to investigate the compactness and structural stability of the proteins. The *R*_g_ values for both complexes remained relatively stable. However, occasional spikes, particularly in 3W2S_W2R, suggest transient conformational expansion. Complex 3W2S_5b recorded an Rg value between 20.23 Å and 25.37 Å, while for complex 3W2S_W2R, it was between 19.95 Å and 25.44 Å. The increased fluctuations in *R*_g_, particularly after 40 ns, may indicate partial unfolding or increased flexibility in certain regions of the protein structure. However, both systems largely maintain compactness. Fig. 6D depicts the number of hydrogen bonds (H-bonds) formed in complexes, which play an important role in maintaining stability and structural integrity. Complex 3W2S_W2R (blue line) consistently develops a greater number of hydrogen bonds during the simulation, ranging from 2 to 7, which suggests strong and stable intermolecular or intramolecular interactions. In contrast, complex 3W2S_5b (red line) exhibits a lower and more variable number of hydrogen bonds, fluctuating between 0 and 3, which indicates weaker and less stable interactions. Fig. 7 shows the Solvent Accessible Surface Area (SASA) analysis, which reveals the conformational changes and stability of 5b and W2R when bound to 3W2S. A lower SASA in the receptor-bound systems indicates that ligand binding reduces the solvent-exposed surface area. The difference in SASA between the unbound and bound systems suggests that ligand binding causes structural rearrangements and decreases solvent accessibility. Notably, W2R binding results in a greater reduction in SASA compared to 5b, suggesting that W2R induces more significant structural changes and compaction upon interaction with the protein.

Protein–ligand interactions between the complexes were monitored by simulation studies for 100 ns summarised by stacked coloured bars, normalized for trajectories that are categorized as ionic interactions, H-bonds, water bridges, and hydrophobic interactions. If the protein–ligand complex has multiple contacts for a particular type of interaction, then the value remains ≥1.

**Fig. 8:**
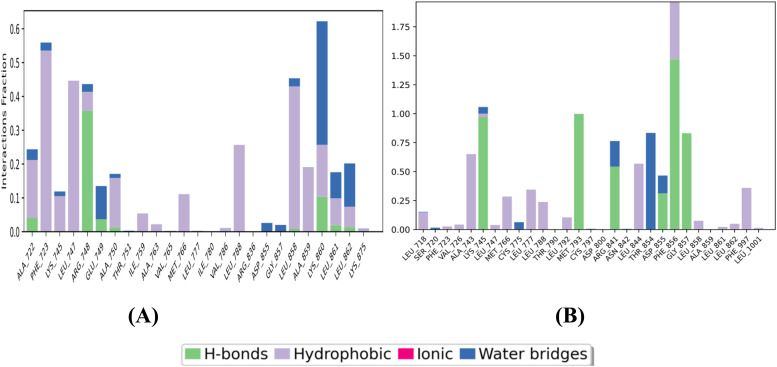
Bar graph of protein–ligand interactions of (A) 3W2S_5b and (B) 3W2S_W2R.

Fig. 8A and B illustrate various bar graphs of various types of interaction fractions against residues present for 100 ns. This reveals that high interaction fractions for protein–ligand complexes 3W2S_5b and 3W2S_W2R were made by hydrogen bonding, ionic interaction and water bridges.

**Fig. 9:**
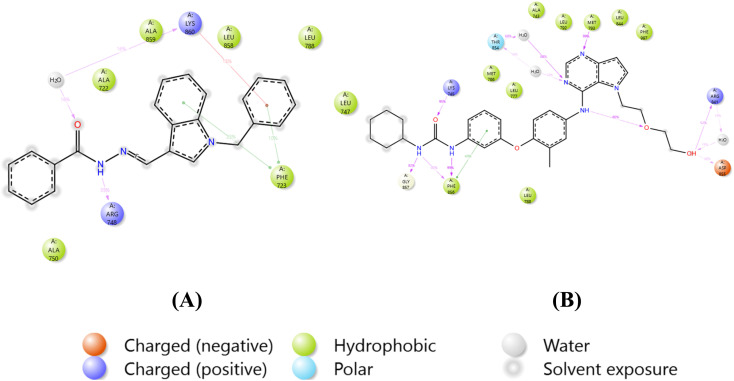
Protein–ligand percent contacts of (A) 3W2S_5b and (B) 3W2S_W2R.

Fig. 9A illustrates the percentage of protein–ligand interactions for the 3W2S_5b complex. LYS860 interacts with 32% of the complex through water bridges and 13% *via* ionic interactions. PHE723 interacts with 33% of the complex through hydrophobic interactions. ARG748 forms 35% of the interactions through hydrogen bonding. Fig. 9A shows the protein–ligand interaction percentages for the 3W2S_W2R complex. Amino acid residues THR854 (16%) and ARG841 (38%) interacted by forming water bridges. PHE856 interacted with 49% *via* ionic interactions and 46% *via* hydrogen bonding. Amino acid residues GLY857 (82%), LYS745 (95%), MET793 (99%), ASP855 (30%), and ARG841 (52%) interacted through hydrogen bonding.

### 2.6. Molecular mechanics generalized Born surface area (MM-GBSA) calculations

**Fig. 10:**
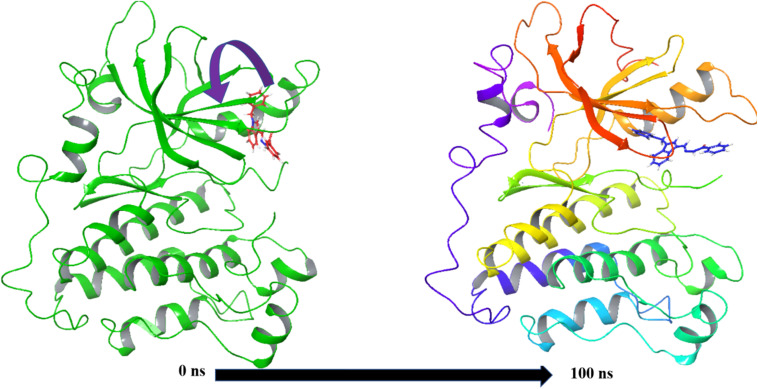
MMGBSA trajectory (0 ns (before simulation) and 100 ns (after simulation)), exhibiting conformational changes of 5b bound to 3W2S protein at the binding site cavity.

MD simulation data were used to calculate different energy values in the form of MM-GBSA for complexes, *i.e.*, for 3W2S_5b and 3W2S_W2R (Fig. 10). The results in Table 2 indicate the binding free energies for complexes 3W2S_5b and 3W2S_W2R. The binding free energy Δ*G*_bind_ of 3W2S_5b was found to be −52.56 kcal mol^−1^. Complex 3W2S_W2R exhibited a significantly more favorable binding energy (Δ*G*_bind_ of −100.07 kcal mol^−1^). The electrostatic contribution to binding (Δ*G*_bindCoulomb_) was notably stronger in complex 3W2S_W2R (−32.77 kcal mol^−1^) than in 3W2S_5b (−16.25 kcal mol^−1^). This suggests that ligand W2R establishes stronger interactions with protein, potentially through salt bridges and polar contacts. A significant difference in van der Waals force contributions with 3W2S_W2R was annotated (stronger vdW stabilization than with 3W2S_5b). Hydrogen bonding and lipophilic contributions were found to play a moderate role in the binding of both complexes, with these interactions being more favorable in the 3W2S_W2R complex than in the 3W2S_5b complex. The positive covalent binding and solvation energy values suggest that both complexes experience unfavorable covalent interactions and solvation effects, respectively. Additionally, the packing contributions were slightly negative in both systems, indicating that both ligands fit well within the binding pocket. Overall, the 3W2S_W2R complex demonstrated a significantly stronger binding affinity than the 3W2S_5b complex primarily due to enhanced van der Waals, coulombic, and lipophilic interactions.

**Table 2:** Binding free energy components for 3W2S_5b and 3W2S_W2R calculated using MM-GBSA

Energies (kcal mol^−1^)	3W2S_5b	3W2S_W2R
Δ*G*_bind_	−52.56 ± 6.57	−100.07 ± 4.89
Δ*G*_bindCoulomb_	−16.25 ± 2.09	−32.77 ± 2.50
Δ*G*_bindCovalent_	3.70 ± 1.76	1.80 ± 0.73
Δ*G*_bindH_bond__	−0.54 ± 0.00	−2.77 ± 0.42
Δ*G*_bindLipo_	−16.16 ± 1.28	−29.37 ± 1.25
Δ*G*_bindPacking_	−0.94 ± 0.58	−0.56 ± 0.23
Δ*G*_bindSolvGB_	21.77 ± 1.77	44.32 ± 2.67
Δ*G*_bindvdW_	−44.14 ± 2.98	−80.72 ± 00202.77

Prior to the simulation (0 ns), 5b was subjected to various angular reorientation and flips to fit within the protein structure. After 100 ns, 5b is fully accommodated within the binding pocket, leading to enhanced interactions with the surrounding residues.

### 2.7. QSAR analysis

“pIC_50_ = 8.615 + −0.233 * com_accminus_2A + −0.06 * fringNlipo6A + −0.005 * sp^3^Cplus_AbSA”, QSAR model 1

com_accminus_2A: the occurrence of negatively charged acceptor atoms with 2 angstrom units from the center of mass of the molecule.

fringNlipo6A: frequency of occurrence of lipophilic atoms within 6 angstrom units from the ring nitrogen atoms.

sp^3^Cplus_AbSA: absolute surface area of the positively charged sp^3^ hybridized carbon atoms.

#### 2.7.1. QSAR model description and validation

The QSAR model outlines the correlation between the pIC_50_ value of a compound and three specific molecular descriptors: com_accminus_2A, fringNlipo6A, and sp^3^Cplus_AbSA. The equation pIC_50_ = 8.615–0.233 * com_accminus_2A − 0.06 * fringNlipo6A – 0.005 * sp^3^Cplus_AbSA indicates that the specified descriptors negatively affect the biological activity of the compound. The com_accminus_2A descriptor quantifies the presence of negatively charged acceptor atoms located within a 2 angstrom radius from the molecular center of mass. An increase in these acceptor atoms correlates with a reduction in pIC_50_, which is likely attributed to unfavorable interactions with the target site. The fringNlipo6A descriptor quantifies the prevalence of lipophilic atoms located within a 6 angstrom radius of ring nitrogen atoms. The negative coefficient associated with this descriptor indicates that increased lipophilicity in proximity to nitrogen rings correlates with reduced activity, which may be attributed to changes in solubility or adverse hydrophobic interactions. Finally, sp^3^Cplus_AbSA quantifies the absolute surface area of positively charged sp^3^ hybridized carbon atoms. Its minor negative coefficient indicates that an increase in these regions slightly reduces activity likely owing to steric or electronic effects that interfere with binding affinity. The intercept value of 8.615 signifies the baseline pIC_50_ when all descriptors are set to zero, reflecting the intrinsic activity of a molecule prior to any structural modifications. This model elucidates the influence of molecular characteristics on bioactivity, facilitating informed drug design. This indicates that reducing the presence of negatively charged acceptor atoms near the molecular centre, minimizing lipophilic regions adjacent to ring nitrogen atoms, and constraining the surface area of positively charged sp^3^ carbons may improve inhibitory potency. Furthermore, the coefficients are relatively small, suggesting that the descriptors have a moderate contribution to activity. This implies that minor structural modifications may result in considerable changes in bioactivity. Full details of the QSAR model (Table 3) are included in the ESI.† The QSAR model illustrates the relationship between charge distribution, lipophilicity, and molecular topology in influencing the potency of chemical compounds.

**Table 3:** QSAR validation parameters obtained for model-1

*R* ^2^_tr	0.8191
Adj-*R*^2^	0.7773
*F*(3−13)	19.6176
RSS_tr	0.0273
MSE_tr	0.0016
RMSE_tr	0.0401
MAE_tr	0.0314
*S*	0.0458
AIC	−51.1214
BIC	−46.9553
CCC_tr	0.9005
*Q* ^2^_cv	0.7214
RMSE_cv	0.0497
MSE_cv	0.0025
PRESS_cv	0.0421
MAE_cv	0.0406
*R* ^2^_Yscr	0.1766
MSE_ex	0.0183
RMSE_ex	0.1353
PRESS_ex	0.0915
*Q* ^2^F1	−1.2346
*Q* ^2^F2	−1.239
*Q* ^2^F3	−1.0593
MAE_ex	0.112
*K*	1.0099
*K*_prime	0.99
R2ext	2.00 × 10^−4^
CCC_ex	−0.0085
*r* ^2^ *m*_ExPy	−1.00 × 10^−4^
*r* ^2^ *m*_EyPx	0
*R* ^2^o	−0.5674
*R* ^2^o_dash	−1.8574
Clos_dash	11342.36
Clos	3465.795
*r* ^2^ *m*_avg	0
*r* ^2^ *m*_delta	1.00 × 10^−4^

## 3. Conclusion

In conclusion, the synthesis, *in vitro*, and *in silico* studies of hydrazones based on *N*-benzyl indole-3-carboxaldehyde 5a–v demonstrated promising potential as anti-TNBC agents. The synthesized compounds exhibited excellent to moderate potencies compared to the TNBC cell line (MDA-MB-231) with IC_50_ ranging from 17.2 ± 0.4 to 43.4 ± 0.2 nM. Compound 5b, which contains a phenyl substitution, demonstrated significant inhibitory activity with an IC_50_ value of 17.2 ± 0.4 μM. Based on these promising results, our current research suggests the potential for further anticancer activity screening particularly by introducing different substituents at the N position of the indole ring in future studies. This approach is especially relevant, as the recently explored hydrazone derivatives may emerge as promising therapeutic candidates for breast cancer treatment.

## 4. Experimental

### 4.1. General

All the chemicals, including indole-3-carboxaldehyde, used to synthesize hydrazones 5a–v were acquired from Sigma Aldrich. EtOH, ether, CH_3_COOC_2_H_5_, glacial CH_3_COOH, MeOH and petroleum ether were bought from Merck and used as received without further purification. MDA-MB-231 cell lines were acquired from the American Type Culture Collection (ATCC) (Cat # CRM-HTB-26), and MCF-10A was purchased from the Iranian Biological Resource Center (IBRC), Tehran, Iran. The reaction's development and conclusion were tracked using silica gel plates supported with aluminum. Using a Bruker Ascend 600 MHz NMR spectrometer (600 MHz for ^1^H and 151 MHz for ^13^C), ^1^H NMR and ^13^C-NMR spectra were recorded at 25 °C in deuterated solvents, such as DMSO-d_6_. To illustrate signal multiplicity, the NMR results are presented as chemical shifts (ppm) and coupling constants (*J*) in hertz.

### 4.2. General procedure for the synthesis of 1-benzyl-1*H*-indole-3-carboxaldehyde (3)

*N*-Benzyl indole-3-carboxaldehyde was synthesized by refluxing a mixture of indole-3-carboxaldehyde (10 mmol, 1.45 g), benzyl bromide (10.85 mmol, 1.29 mL), anhydrous K2CO3 (1.4 g), and dimethylformamide (10 mL) for 6 hours with continuous stirring. Once the reaction was complete, as confirmed by the TLC, the mixture was cooled and poured into water. The resulting solid was filtered, washed, and allowed to dry.

### 4.3. General procedure for the synthesis of 1-benzyl-1*H*-indole-3-carboxaldehyde-based hydrazones 5a–v

Ten milliliters of ethanol and 0.4 mmol (0.1 g) of one-benzyl-1*H*-indole-3-carboxaldehyde were placed in a round-bottom flask and refluxed until the aldehyde completely dissolved, resulting in a clear solution. Next, hydrazide 5a–v (0.4 mmol) was added to the refluxing mixture, along with a catalytic amount of acetic acid (CH_3_COOH). This resulted in the formation of precipitates. The progress of the reaction was tracked using TLC plates with a solvent system of ethyl acetate and petroleum ether in a 1 : 1 ratio. After the reaction was complete, the mixture was allowed to cool, and the precipitates were filtered out. After washing the precipitates with ethanol, they were dried and weighed, and the yield was calculated.

#### 4.3.1 (*E*)-*N*′-[(1-benzyl-1*H*-indol-3-ylmethylene]-4-methoxybenzohydrazide (5a)

Yield = 97%, mp = 226–228 °C, white solid, IR (KBr) cm^−1^; 3390, 2349, 2048, 1630, 1050, 903; *δ*_H_ (600 MHz, DMSO-d_6_) 11.45 (1H, s), 8.61 (1H, s), 8.32 (1H, d, *J* = 7.7 Hz), 8.01 (1H, s), 7.97–7.85 (2H, m), 7.52 (1H, d, *J* = 8.1 Hz), 7.33 (2H, t, *J* = 7.5 Hz), 7.27 (3H, d, *J* = 7.4 Hz), 7.23 (1H, t, *J* = 7.2 Hz), 7.19 (1H, t, *J* = 7.4 Hz), 7.14–7.01 (2H, m), 5.47 (2H, s), 3.84 (3H, s); ^13^C NMR (151 MHz, DMSO) *δ* 162.48, 162.20, 144.21, 138.06, 137.33, 133.54, 129.80, 129.11, 128.03, 127.62, 126.49, 125.53, 123.30, 122.73, 121.21, 114.13, 111.95, 111.12, 55.87, 49.81. QTOF HRMS (*m*/*z*): [M + H]^+^, calcd: 384.1712, found: 384.1746.

#### 4.3.2 (*E*)-*N*′-[(1-benzyl-1*H*-indo1-3-yl)methylene]benzohydrazide (5b)

Yield = 97%, mp = 187–189 °C, light brown solid, IR (KBr) cm^−1^; 3452, 3187, 2837, 1622, 1385, 1162, 938; *δ*_H_ (600 MHz, DMSO-d_6_) 11.57 (1H, s), 8.63 (1H, s), 8.33 (1H, d, *J* = 7.7 Hz), 8.03 (1H, s), 7.98–7.88 (2H, m), 7.64–7.49 (4H, m), 7.38–7.18 (7H, m), 5.48 (2H, s); ^13^C NMR (151 MHz, DMSO) *δ* 163.04, 144.79, 138.04, 137.34, 134.46, 133.78, 131.85, 129.12, 128.90, 128.04, 127.95, 127.63, 125.52, 123.34, 122.73, 121.27, 111.85, 111.16, 49.82. QTOF HRMS (*m*/*z*): [M + H]^+^, calcd: 354.1606, found: 354.1619.

#### 4.3.3 (*E*)-*N*′-((l-benzyl-1*H*-indo1-3-yl)methylenel-3-fluoro-4-methylbenzohydrazide (5c)

Yield = 84%, mp = 203–205 °C, white solid, IR (KBr) cm^−1^; 3428, 3221,1609, 1385, 1274, 995, 834, 729; *δ*_H_ (600 MHz, DMSO-d_6_) 11.57 (1H, s), 8.62 (1H, s), 8.37–8.26 (1H, m), 8.05 (1H, s), 7.75–7.66 (2H, m), 7.53 (1H, d, *J* = 8.1 Hz), 7.46 (1H, t, *J* = 7.9 Hz), 7.34 (2H, dd, *J* = 8.3, 6.7 Hz), 7.30–7.17 (5H, m), 5.48 (2H, s), 2.32 (3H, s); ^13^C NMR (151 MHz, DMSO) *δ* 161.53, 160.01, 145.00, 138.05, 137.36, 134.03, 133.91, 132.17, 132.14, 129.11, 128.54, 128.42, 128.04, 127.63, 125.51, 123.88, 123.85, 123.34, 122.71, 121.30, 114.43, 114.27, 111.80, 111.17, 49.83, 14.72. QTOF HRMS (*m*/*z*): [M + H]^+^, calcd: 386.1668, found: 386.1688.

#### 4.3.4 (*E*)-*N*′-[(1-benzyl-1*H*-indo1-3-yl)methylene]-2-(4(trifluoromethyl)phenyl)acetohydrazide (5d)

Yield = 74%, mp = 231–233 °C, light yellow solid, IR (KBr) cm^−1^; 3438, 3019, 1609, 1357, 1120, 841, 716; *δ*_H_ (600 MHz, DMSO-d_6_) 11.77 (1H, s), 8.64 (1H, s), 8.39–8.31 (1H, m), 8.14 (2H, d, *J* = 8.1 Hz), 8.07 (1H, s), 7.93 (3H, d, *J* = 8.1 Hz), 7.58–7.52 (1H, m), 7.38–7.17 (7H, m), 5.49 (2H, s); ^13^C NMR (151 MHz, DMSO) *δ* 161.79, 145.56, 138.31, 138.02, 137.39, 134.16, 131.77, 131.56, 130.07, 129.12, 129.07, 128.90, 128.05, 127.64, 127.54, 125.92, 125.89, 125.87, 125.50, 125.34, 123.54, 123.39, 122.71, 121.37, 111.71, 111.21, 49.85. QTOF HRMS (*m*/*z*): [M + H]^+^, calcd: 436.1636, found: 436.1651.

#### 4.3.5 (*E*)-*N*′-[(1-benzyl-1*H*-indo1-3-yl)methylene]nicotinohydrazide (5e)

Yield = 95%, mp = 134–136 °C, yellow solid, IR (KBr) cm^−1^; 3445, 3186, 3019, 1622, 1363, 1154, 1049, 930, 720; *δ*_H_ (600 MHz, DMSO-d_6_) 11.74 (1H, s), 9.09 (1H, s), 8.76 (1H, s), 8.62 (1H, s), 8.07 (1H, s), 7.62–7.51 (2H, m), 7.38–7.19 (8H, m), 5.49 (2H, s); ^13^C NMR (151 MHz, DMSO) *δ* 161.48, 152.42, 148.96, 145.38, 138.02, 137.38, 135.78, 134.13, 129.12, 129.09, 128.05, 127.64, 127.57, 125.50, 124.06, 123.38, 122.70, 121.37, 111.69, 111.21, 49.85. QTOF HRMS (*m*/*z*): [M + H]^+^, calcd: 355.1558, found: 355.1573.

#### 4.3.6 (*E*)-*N*′-[(1-benzyl-1*H*-indo1-3-yl)methylene]-4-bromobenzohydrazide (5f)

Yield = 90%, mp = 240–242 °C, off white solid, IR(KBr) cm^−1^; 3459, 3214, 3061, 2830, 1922, 1615, 1370, 1168, 1056, 714; *δ*_H_ (600 MHz, DMSO-d_6_) 11.64 (1H, s), 8.62 (1H, s), 8.35–8.29 (1H, m), 8.05 (1H, s), 7.93–7.86 (2H, m), 7.80–7.71 (2H, m), 7.53 (1H, d, *J* = 8.0 Hz), 7.33 (2H, td, *J* = 7.1, 1.7 Hz), 7.31–7.26 (3H, m), 7.25–7.17 (2H, m), 5.48 (2H, s); ^13^C NMR (151 MHz, DMSO) *δ* 162.00, 145.11, 138.03, 137.37, 133.96, 133.54, 131.91, 130.08, 129.12, 128.04, 127.64, 125.55, 125.51, 123.35, 122.72, 121.31, 111.79, 111.18, 49.84. QTOF HRMS (*m*/*z*): [M + H]^+^, calcd: 432.0711, found: 432.0717.

#### 4.3.7 (*E*)-*N*′-[(1-benzyl-1*H*-indo1-3-yl)methylene]-3-hydroxy-2-naphthohydrazide (5g)

Yield = 95%, mp = 239–241 °C, off white solid, IR (KBr) cm^−1^; 3452, 3033, 1609, 1364, 1183, 1050, 951, 721; *δ*_H_ (600 MHz, DMSO-d_6_) 11.86 (1H, s), 11.63 (1H, s), 8.67 (1H, s), 8.52 (1H, s), 8.38–8.33 (1H, m), 8.08 (1H, s), 7.93 (1H, d, *J* = 8.1 Hz), 7.78 (1H, d, *J* = 8.3 Hz), 7.58–7.50 (2H, m), 7.38 (1H, ddd, *J* = 8.1, 6.8, 1.2 Hz), 7.36–7.32 (3H, m), 7.30–7.20 (6H, m), 5.50 (2H, s); ^13^C NMR (151 MHz, DMSO) *δ* 164.10, 155.25, 145.78, 138.00, 137.41, 136.32, 134.29, 130.08, 129.15, 129.13, 129.12, 128.65, 128.07, 127.66, 127.64, 127.19, 126.34, 125.52, 124.23, 123.44, 122.73, 121.44, 120.17, 111.68, 111.25, 111.12, 49.87. QTOF HRMS (*m*/*z*): [M + H]^+^, calcd: 420.1712, found: 420.1734.

#### 4.3.8 (*E*)-*N*′-[(1-benzyl-1*H*-indo1-3-yl)methylene]-4-(trifluoromethyl)benzohydrazide (5h)

Yield = 90%, mp = 234–236 °C, off white solid, IR (KBr) cm^−1^; 3445, 3180, 3026, 2860, 2350, 1609, 1350, 1141, 862; *δ*_H_ (600 MHz, DMSO-d_6_) 11.77 (1H, s), 8.64 (1H, s), 8.37–8.30 (1H, m), 8.17–8.10 (2H, m), 8.07 (1H, s), 7.93 (2H, d, *J* = 8.1 Hz), 7.54 (1H, dd, *J* = 7.8, 1.2 Hz), 7.37–7.30 (2H, m), 7.30–7.19 (5H, m), 5.49 (2H, s); ^13^C NMR (151 MHz, DMSO) *δ* 161.79, 145.56, 138.31, 138.02, 137.39, 134.16, 129.12, 129.07, 128.90, 128.05, 127.64, 125.92, 125.89, 125.87, 125.49, 123.39, 122.71, 121.37, 111.71, 111.21, 49.85. QTOF HRMS (*m*/*z*): [M + H]^+^, calcd: 422.1480, found: 422.1503.

#### 4.3.9 (*E*)-*N*′-[(1-benzyl-1*H*-indo1-3-yl)methylene]furan-2-carbohydrazide (5i)

Yield = 97%, mp = 204–206 °C, off white solid, IR (KBr) cm^−1^; 3438, 3026, 2341, 1929, 1636, 1370, 1168, 1000, 839; *δ*_H_ (600 MHz, DMSO-d_6_) 11.56 (1H, s), 8.63 (1H, s), 8.28 (1H, d, *J* = 7.7 Hz), 8.02 (1H, s), 7.93 (1H, d, *J* = 1.7 Hz), 7.53 (1H, d, *J* = 8.1 Hz), 7.35–7.31 (2H, m), 7.30–7.25 (4H, m), 7.21 (2H, dddd, *J* = 21.9, 8.0, 7.1, 1.3 Hz), 6.70 (1H, dd, *J* = 3.5, 1.7 Hz), 5.48 (2H, s); ^13^C NMR (151 MHz, DMSO) *δ* 154.24, 147.63, 145.84, 144.88, 138.04, 137.33, 133.80, 129.11, 128.03, 127.64, 125.49, 123.33, 122.67, 121.29, 114.60, 112.44, 111.80, 111.17, 49.82. QTOF HRMS (*m*/*z*): [M + H]^+^, calcd: 344.1399, found: 344.1414.

#### 4.3.10 (*E*)-*N*′-[(1-benzyl-1*H*-indo1-3-yl)methylene]-3-chlorobenzohydrazide (5j)

Yield = 97%, mp = 215–217 °C, white solid, IR (KBr) cm^−1^; 3431, 3012, 1755, 1622, 1475, 1300, 1174, 986; *δ*_H_ (600 MHz, DMSO-d_6_) 11.66 (1H, s), 8.62 (1H, s), 8.31 (1H, d, *J* = 7.7 Hz), 8.05 (1H, s), 8.00–7.94 (1H, m), 7.89 (1H, d, *J* = 7.7 Hz), 7.69–7.63 (1H, m), 7.58 (1H, t, *J* = 7.9 Hz), 7.53 (1H, d, *J* = 8.1 Hz), 7.33 (2H, q, *J* = 7.1, 6.3 Hz), 7.28 (3H, d, *J* = 7.6 Hz), 7.22 (2H, dt, *J* = 21.8, 7.1 Hz), 5.48 (2H, s); ^13^C NMR (151 MHz, DMSO) *δ* 161.54, 145.31, 138.01, 137.36, 136.45, 134.05, 133.69, 131.69, 130.95, 129.12, 128.05, 127.65, 127.63, 126.80, 125.49, 123.38, 122.70, 121.35, 111.72, 111.20, 49.84. QTOF HRMS (*m*/*z*): [M + H]^+^, calcd: 388.1216, found: 388.1284.

#### 4.3.11 (*E*)-*N*′-[(1-benzyl-1*H*-indo1-3-yl)methylene]isonicotinohydrazide (5k)

Yield = 80% mp = 241–243 °C, yellow solid, IR (KBr) cm^−1^; 3431, 3193, 3005, 1615, 1350, 1175, 924, 707; *δ*_H_ (600 MHz, DMSO-d_6_) 11.79 (1H, s), 8.81–8.76 (2H, m), 8.64 (1H, s), 8.31 (1H, dt, *J* = 7.6, 1.0 Hz), 8.08 (1H, s), 7.87–7.81 (2H, m), 7.56–7.52 (1H, m), 7.37–7.32 (2H, m), 7.31–7.26 (3H, m), 7.22 (2H, dddd, *J* = 20.2, 8.1, 7.1, 1.2 Hz), 5.49 (2H, s); ^13^C NMR (151 MHz, DMSO) *δ* 161.35, 150.74, 145.95, 141.50, 138.00, 137.40, 134.34, 129.13, 128.06, 127.65, 127.55, 125.47, 123.41, 122.69, 121.97, 121.42, 111.62, 111.24, 49.86. QTOF HRMS (*m*/*z*): [M + H]^+^, calcd: 355.1558, found: 355.1569.

#### 4.3.12 (*E*)-*N*′-[(1-benzyl-1*H*-indo1-3-yl)methylene]-4-nitrobenzohydrazide (5l)

Yield = 95%, mp = 248–250 °C, off white solid, IR (KBr) cm^−1^; 3431, 1615, 1357, 1070, 812, 651; *δ*_H_ (600 MHz, DMSO-d_6_) 11.53 (1H, s), 11.36 (1H, s), 8.30–8.24 (1H, m), 8.24–8.17 (1H, m), 8.02 (1H, d, *J* = 4.4 Hz), 7.88 (1H, s), 7.57–7.48 (3H, m), 7.36–7.29 (3H, m), 7.27 (3H, tdd, *J* = 6.8, 4.7, 1.9 Hz), 7.24–7.12 (3H, m), 7.10–7.02 (1H, m), 6.89 (1H, td, *J* = 7.4, 1.2 Hz), 6.82 (1H, td, *J* = 7.4, 1.2 Hz), 6.32–6.27 (1H, m), 5.46 (2H, s), 4.19 (1H, s), 3.70 (1H, s); ^13^C NMR (151 MHz, DMSO) *δ* 172.57, 167.47, 144.58, 143.66, 143.47, 141.56, 137.97, 137.62, 137.49, 137.40, 137.32, 134.27, 134.02, 131.32, 130.89, 130.44, 130.03, 129.70, 129.61, 129.12, 129.10, 128.06, 128.04, 127.87, 127.77, 127.65, 127.61, 125.91, 125.69, 125.65, 125.42, 125.13, 125.01, 123.34, 122.48, 122.14, 121.49, 121.31, 121.07, 116.58, 116.10, 111.45, 111.30, 111.18, 49.82, 38.86, 35.98. QTOF HRMS (*m*/*z*): [M + H]^+^, calcd: 527.1405, found: 527.2863.

#### 4.3.13 (*E*)-*N*′-[(1-benzyl-1*H*-indo1-3-yl)methylene]-2-bromobenzohydrazide (5m)

Yield = 95%, mp = 226–228 °C, white solid, IR (KBr) cm^−1^; 3389, 3144, 2823, 1601, 1595, 1398, 1160, 908, 811; *δ*_H_ (600 MHz, DMSO-d_6_) 11.69 (1H, s), 11.59 (1H, s), 8.44 (1H, s), 8.30 (1H, d, *J* = 7.7 Hz), 8.01 (1H, s), 7.77–7.69 (1H, m), 7.59–7.48 (2H, m), 7.46–7.38 (2H, m), 7.36–7.17 (6H, m), 7.11 (1H, d, *J* = 8.0 Hz), 7.07 (1H, t, *J* = 7.9 Hz), 6.75 (1H, t, *J* = 7.5 Hz), 5.47 (1H, s), 5.39 (1H, s); ^13^C NMR (151 MHz, DMSO) *δ* 169.41, 163.20, 144.84, 140.66, 139.54, 138.39, 138.01, 137.98, 137.36, 137.17, 134.13, 133.57, 133.25, 132.40, 131.73, 130.57, 129.81, 129.12, 129.05, 128.82, 128.19, 128.06, 127.97, 127.87, 127.64, 127.51, 125.44, 125.09, 123.40, 123.10, 122.72, 122.35, 121.36, 120.78, 120.04, 119.37, 111.68, 111.60, 111.20, 110.91, 49.84, 49.71. QTOF HRMS (*m*/*z*): [M + 2]^+^, calcd: 432.0711, found: 434.0921.

#### 4.3.14 (E)-*N*′-(l-benzyl-1*H*-indo1-3-yl)methylenel-2-phenylacetohydrazide (5n)

Yield = 91%, mp = 213–215 °C, white solid, IR (KBr) cm^−1^; 3445, 3068, 2914, 1644, 1385, 1162, 953; *δ*_H_ (600 MHz, DMSO-d_6_) 11.36 (1H, s), 11.11 (1H, s), 11.32 (1H, s), 8.21 (1H, s), 8.20–8.15 (1H, m), 7.97 (1H, d, *J* = 2.6 Hz), 7.56–7.48 (1H, m), 5.45 (2H, s), 4.03 (1H, s), 3.52 (1H, s); ^13^C NMR (151 MHz, DMSO) *δ* 171.95, 166.24, 143.54, 140.38, 138.03, 138.02, 137.34, 137.30, 136.55, 136.53, 133.78, 133.62, 129.79, 129.49, 129.10, 128.76, 128.70, 128.03, 127.62, 126.98, 126.80, 125.43, 125.19, 123.29, 122.58, 122.24, 121.38, 121.18, 111.68, 111.61, 111.24, 111.11, 49.78, 41.79. QTOF HRMS (*m*/*z*): [M + H]^+^, calcd: 368.1762, found: 368.1779.

#### 4.3.15 (*E*)-*N*′-((l-benzyl-1*H*-indo1-3-yl)methylenel-2-chlorobenzohydrazide (5o)

Yield = 88%, mp = 201–203 °C, off white shiny solid, IR (KBr) cm^−1^; 3445, 3054, 1636, 1371, 1162, 925; *δ*_H_ (600 MHz, DMSO-d_6_) 11.71 (1H, s), 11.61 (1H, s), 8.44 (1H, s), 8.02 (1H, s), 7.60–7.55 (1H, m), 7.55–7.50 (1H, m), 7.49–7.44 (1H, m), 7.32 (1H, q, *J* = 8.1 Hz), 7.28 (2H, t, *J* = 7.2 Hz), 7.23 (1H, ddd, *J* = 14.3, 6.7, 1.3 Hz), 7.21–7.17 (1H, m), 6.80–6.72 (1H, m), 5.47 (1H, s), 5.40 (1H, s); ^13^C NMR (151 MHz, DMSO) *δ* 168.69, 162.30, 144.84, 140.74, 138.02, 137.99, 137.36, 137.17, 136.26, 134.15, 133.63, 131.61, 130.92, 130.51, 130.15, 130.11, 129.83, 129.32, 129.12, 129.05, 128.77, 128.05, 127.97, 127.73, 127.65, 127.51, 127.42, 125.44, 125.06, 123.38, 123.09, 122.70, 122.27, 121.35, 120.78, 111.63, 111.58, 111.20, 110.93, 49.83, 49.70. QTOF HRMS (*m*/*z*): [M + H]^+^, calcd: 388.1216, found: 388.1232.

#### 4.3.16 (*E*)-*N*′-[(1-benzyl-1*H*-indo1-3-yl)methylene]-2-(2-(trifluoromethyl)phenypacetohydrazide (5p)

Yield = 90%, mp = 212–214 °C, off white solid, IR (KBr) cm^−1^; 3431, 3200, 3005, 2858, 1622, 1357, 1168, 1049, 930, 713; *δ*_H_ (600 MHz, DMSO-d_6_) 11.35 (1H, s), 11.23 (1H, s), 8.23 (1H, s), 8.11 (1H, d, *J* = 7.9 Hz), 7.99 (1H, d, *J* = 2.1 Hz), 7.73 (1H, dd, *J* = 8.0, 3.9 Hz), 7.65 (1H, t, *J* = 7.6 Hz), 7.57–7.46 (3H, m), 7.36–7.30 (2H, m), 7.27 (3H, ddd, *J* = 8.8, 4.8, 2.4 Hz), 7.20 (1H, ddd, *J* = 8.3, 7.0, 1.4 Hz), 7.17–7.09 (1H, m), 5.46 (2H, s), 4.29 (2H, d, *J* = 2.1 Hz), 3.80 (1H, s); ^13^C NMR (151 MHz, DMSO) *δ* 170.93, 165.17, 143.39, 140.38, 138.06, 138.04, 137.37, 134.89, 133.95, 133.86, 133.65, 133.61, 132.70, 132.61, 129.11, 129.09, 128.01, 127.70, 127.63, 127.59, 126.03, 125.96, 125.44, 125.21, 124.15, 123.29, 122.19, 121.29, 121.16, 111.69, 111.61, 111.24, 49.78, 38.08, 36.56. QTOF HRMS (*m*/*z*): [M + H]^+^, calcd: 436.1636, found: 436.1663.

#### 4.3.17 (*E*)-*N*′-[(1-benzyl-1*H*-indo1-3-yl)methylene]-2-(2,4-difluorophenypacetohydrazide (5q)

Yield = 76%, mp = 208–210 °C, white solid, IR (KBr) cm^−1^; 3445, 3082, 2914, 1644, 1399, 1133,9 58, 746; *δ*_H_ (600 MHz, DMSO-d_6_) 11.22 (1H, s), 8.24–8.19 (1H, m), 8.15 (1H, dt, *J* = 7.8, 1.0 Hz), 7.99 (1H, d, *J* = 1.2 Hz), 7.52 (1H, dq, *J* = 8.2, 1.8, 1.4 Hz), 7.49–7.42 (1H, m), 7.36–7.30 (2H, m), 7.26 (3H, td, *J* = 7.1, 3.7 Hz), 7.21 (2H, dddd, *J* = 10.8, 8.4, 3.2, 1.2 Hz), 7.18–7.13 (1H, m), 7.09–7.03 (1H, m), 5.46 (2H, s), 4.08 (1H, s), 3.60 (1H, s); ^13^C NMR (151 MHz, DMSO) *δ* 170.65, 165.10, 162.56, 162.47, 162.06, 161.98, 160.93, 160.85, 160.43, 160.34, 143.55, 140.55, 138.03, 137.37, 137.32, 133.87, 133.69, 133.54, 133.50, 133.48, 133.44, 133.30, 129.11, 129.09, 128.02, 127.63, 127.60, 125.44, 125.21, 123.29, 122.58, 122.32, 121.39, 121.19, 120.07, 120.04, 119.96, 119.93, 111.72, 111.66, 111.60, 111.58, 111.56, 111.45, 111.42, 111.21, 111.12, 104.19, 104.08, 104.02, 103.91, 103.85, 103.74, 49.78, 34.12, 32.50. QTOF HRMS (*m*/*z*): [M + H]^+^, calcd: 404.1574, found: 404.1592.

#### 4.3.18 (*E*)-*N*′-[(1-benzyl-1*H*-indo1-3-yl)methylene]-2-(3,5-difluorophenyl)acetohydrazide (5r)

Yield = 89%, mp = 224–226 °C, white solid, IR (KBr) cm^−1^; 3459, 3068, 1643, 1364, 1141, 938, 819, 714; *δ*_H_ (600 MHz, DMSO-d_6_) 11.34 (1H, s), 11.22 (1H, s), 8.22 (1H, d, *J* = 4.5 Hz), 8.18–8.12 (1H, m), 7.99 (1H, d, *J* = 1.7 Hz), 7.52 (1H, dd, *J* = 8.3, 4.9 Hz), 7.36–7.29 (2H, m), 7.26 (3H, td, *J* = 6.2, 1.6 Hz), 7.24–7.10 (3H, m), 7.08 (2H, td, *J* = 8.8, 8.4, 3.3 Hz), 5.46 (2H, d, *J* = 2.4 Hz), 4.11 (1H, s), 3.60 (1H, s); ^13^C NMR (151 MHz, DMSO) *δ* 170.91, 165.17, 163.50, 163.45, 163.36, 161.88, 161.82, 161.79, 161.73, 143.87, 141.05, 140.98, 140.91, 140.84, 140.71, 138.03, 138.01, 137.37, 137.33, 133.95, 133.79, 129.10, 128.03, 127.63, 127.62, 125.43, 125.17, 123.31, 123.29, 122.58, 122.29, 121.40, 121.22, 113.35, 113.32, 113.22, 113.19, 112.91, 112.88, 112.78, 112.75, 111.62, 111.53, 111.24, 111.13, 102.71, 102.54, 102.48, 102.31, 102.14, 49.79, 41.09, 39.06. QTOF HRMS (*m*/*z*): [M + H]^+^, calcd: 404.1574, found: 404.1598.

#### 4.3.19 (*E*)-*N*′-[(1-benzyl-1*H*-indo1-3-yl)methylene]-2-(2,5-difluorophenyl)acetohydrazide (5s)

Yield = 54%, mp = 169–171 °C, off white solid, IR (KBr) cm^−1^; 3445, 3180, 2349, 1630, 1399, 1176, 917, 729; *δ*_H_ (600 MHz, DMSO-d_6_) 10.18 (1H, s), 9.95 (1H, s), 8.49 (1H, s), 8.16–8.08 (1H, m), 7.63–7.57 (1H, m), 7.39–7.21 (8H, m), 5.56 (2H, s); ^13^C NMR (151 MHz, DMSO) *δ* 185.19, 168.09, 141.48, 137.44, 137.25, 129.22, 128.28, 127.83, 125.28, 124.11, 123.04, 121.56, 117.88, 111.89, 50.28, 33.50. QTOF HRMS (*m*/*z*): [M + H]^+^, calcd: 404.1574, found: 404.1596.

#### 4.3.20 (*E*)-*N*′-[(1-benzyl-1*H*-indo1-3-yl)methylene]-2-(7-methoxynaphthalen-2-yl)propanehydrazide (5t)

Yield = 97%, mp = 195–197 °C, white solid, IR (KBr) cm^−1^; 3431, 3130,1636, 1391, 1174, 1049, 832, 748; *δ*_H_ (600 MHz, DMSO-d_6_) 11.28 (1H, s), 11.04 (1H, s), 8.37 (1H, s), 8.23–8.17 (2H, m), 8.14 (1H, s), 7.95 (1H, s), 7.91 (1H, s), 7.84–7.77 (4H, m), 7.74 (2H, dd, *J* = 8.7, 5.7 Hz), 7.55–7.47 (4H, m), 7.33–7.28 (5H, m), 7.24 (10H, ddt, *J* = 12.1, 6.1, 3.9 Hz), 7.17–7.09 (3H, m), 5.43 (5H, d, *J* = 10.5 Hz), 4.84 (1H, d, *J* = 7.1 Hz), 3.86 (3H, s), 3.83 (4H, s), 1.50 (7H, dd, *J* = 7.1, 1.2 Hz); ^13^C NMR (151 MHz, DMSO) *δ* 174.62, 169.50, 157.50, 157.42, 143.54, 140.23, 138.05, 138.01, 137.88, 137.53, 137.30, 137.28, 133.74, 133.69, 133.53, 133.51, 129.60, 129.47, 129.09, 129.07, 128.92, 128.86, 128.01, 127.98, 127.60, 127.56, 127.20, 127.12, 126.88, 126.01, 125.84, 125.43, 125.13, 123.26, 122.58, 122.36, 121.29, 121.14, 119.13, 119.06, 111.72, 111.58, 111.17, 111.09, 106.16, 106.14, 55.61, 55.59, 49.76, 49.74, 44.37, 19.36, 19.07. QTOF HRMS (*m*/*z*): [M + H]^+^, calcd: 462.2181, found: 462.2205.

#### 4.3.21 (*E*)-*N*′-[(1-benzyl-1*H*-indo1-3-yl)methylene]-1-naphthohydrazide (5u)

Yield = 90%, mp = 234–236 °C, off white solid, IR (KBr) cm^−1^; 3452, 3166, 1601, 1343, 1175, 1028, 924, 757; *δ*_H_ (600 MHz, DMSO-d_6_) 11.74 (1H, s), 11.72 (1H, s), 8.52 (1H, s), 8.41–8.36 (1H, m), 8.29–8.24 (1H, m), 8.09 (1H, dt, *J* = 8.3, 1.1 Hz), 8.03 (2H, d, *J* = 7.2 Hz), 7.76 (1H, dd, *J* = 7.0, 1.2 Hz), 7.65–7.58 (4H, m), 7.58–7.50 (2H, m), 7.37–7.30 (2H, m), 7.30–7.20 (6H, m), 7.18–7.13 (1H, m), 6.54–6.45 (1H, m), 5.48 (2H, s), 5.35 (1H, s); ^13^C NMR (151 MHz, DMSO) *δ* 170.78, 164.54, 144.62, 140.28, 138.04, 138.01, 137.38, 137.03, 135.27, 134.00, 133.95, 133.65, 133.39, 133.31, 130.59, 130.55, 129.95, 129.25, 129.12, 129.01, 128.78, 128.56, 128.05, 127.93, 127.66, 127.46, 127.41, 127.03, 126.85, 126.42, 126.11, 125.96, 125.77, 125.52, 125.42, 124.85, 123.36, 122.90, 122.78, 122.18, 121.32, 120.49, 111.77, 111.63, 111.19, 110.73, 49.83, 49.64. QTOF HRMS (*m*/*z*): [M + H]^+^, calcd: 404.1762, found: 404.1782.

#### 4.3.22 (*E*)-*N*′-[(1-benzyl-1*H*-indo1-3-yl)methylene]-2-methylbenzohydrazide (5v)

Yield = 91%, mp = 205–207 °C, white shiny solid, IR (KBr) cm^−1^; 3445, 3207, 2335, 3428, 1622, 1378, 1287, 1168, 923, 734; *δ*_H_ (600 MHz, DMSO-d_6_) 11.55 (1H, s), 11.44 (1H, s), 8.47 (1H, s), 8.32 (1H, ddd, *J* = 7.7, 1.4, 0.7 Hz), 7.99 (1H, s), 7.53 (1H, dt, *J* = 8.2, 1.0 Hz), 7.45 (1H, dd, *J* = 7.5, 1.4 Hz), 7.43–7.36 (2H, m), 7.36–7.17 (13H, m), 5.47 (2H, s), 5.40 (1H, s), 2.41 (3H, s), 2.28 (1H, s); ^13^C NMR (151 MHz, DMSO) *δ* 171.50, 165.06, 144.22, 140.32, 138.05, 137.50, 137.34, 137.17, 136.42, 136.21, 134.53, 133.79, 133.43, 130.99, 130.07, 129.96, 129.11, 129.05, 128.97, 128.03, 127.97, 127.88, 127.64, 127.53, 127.27, 126.09, 125.63, 125.48, 125.06, 123.32, 123.08, 122.74, 122.32, 121.25, 120.80, 111.78, 111.74, 111.15, 110.91, 49.81, 49.70, 19.81. QTOF HRMS (*m*/*z*): [M + H]^+^, calcd: 368.1762, found: 386.1785.

### 4.4. *In vitro* cytotoxicity assay of synthetic derivatives

The *in vitro* cytotoxicity of the synthetic derivatives was evaluated using an MTT assay against a breast cancer cell line (MDA-MB-231).^33^ The normal human breast cell line (MCF-10A) was maintained as a control in this study.^34,35^ The cells were grown in DMEM with 10% FBS and 1% antibiotics (100 U mL^−1^ penicillin). They were plated in a 96-well plate at a density of 1.0 × 10^4^ cells per well and incubated for 24 hours at 37 °C in a 5% CO_2_ atmosphere. After discarding the medium, both cell lines were treated with various concentrations (6.5 μM, 12.5 μM, 25 μM, and 50 μM) of synthetic triazole derivatives.^36^ After 48 h of incubation, ^37^ 20 μL of MTT solution (5 mg mL^−1^) was added to each well and incubated for an additional 4 hours. After incubation, the medium was removed, and the formazan precipitate was dissolved in DMSO. The absorbance of the mixture was then measured at 570 nm using a microplate reader. All experiments were performed in triplicate, and the cytotoxicity was expressed as a percentage of cell viability compared to untreated control cells as per an earlier reported protocol by Sakhi *et al.*:^36^1
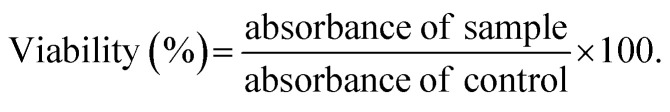


### 4.5. Computational methods

#### 4.5.1 Molecular docking simulations

The 2D structures of the synthesized compounds were drawn using ChemDraw 20.1.1 and optimized using MarvinSketch (ChemAxon, Version 22.13). Hydrogens were added, and all 2D structures were then converted to 3D. The target EGFR crystal structure was retrieved from the RCSB Protein Data Bank^38^ (pyrrolo[3,2-*d*]pyrimidine-based inhibitor bound with EGFR T790M/L858R mutant through PDB ID: 3W2S). We validated the same by examining protein resolution and wwPDB scores and observing missing residues in binding sites on PDBsum.^39^ To find the grid pocket, AutoDockTools 1.5.6,^40^ Chimera,^41^ and Maestro^42^ were employed, where W2R was positioned as the co-crystalized ligand at size 20 × 20 × 20 Å, pointing in the *x*, *y*, and *z* directions, respectively, with a grid point spacing of 0.375 Å and the dimensions at the center (*x* = 5.07, *y* = 1.11, and *z* = 10.35, respectively). Docking simulations were performed using AutoDock Vina^43,44^ Version 1.2.5 through a Windows operating system in triplicate on preset conditions of CPU speed, grid size, search intensity, mode quantity, and energy limits using different grid sizes to verify reliability and repeatability. A post-docking analysis was performed through in-house Python scripts created by AutoDockTools to process the docking results, which enabled us to split and form protein and ligand connections and corresponding protein–ligand complexes subjected to Discovery Studio,^45^ PLIP,^46^ and MAESTRO visualization programs. Prime-MMGBSA analysis was performed using the ‘Prime Module’ (Prime, Schrodinger, LLC, NY, 2023).

#### 4.5.2 GA-MLR QSAR study

The dataset was utilized to develop GA-MLR (genetic algorithm multiple linear regression) models using the widely used software QSARINS version 2.2.2, and these models were validated both internally and externally. This methodological approach is similar to that described in an earlier publication.^47–49^

#### 4.5.3 Molecular dynamics simulation (MDS)

Desmond 2020.1 from Schrödinger, LLC was used to run MD simulations on dock complex for 3W2S, with 5b and W2R represented as 3W2S_5b and 3W2S_W2R, respectively. The OPLS-2005 force field along with an explicit solvent model using TIP3P water molecules was employed in this system, which was set up in a periodic boundary box measuring 10 Å × 10 Å × 10 Å. To neutralize the charge, Na^+^ ions were introduced at a concentration of 0.15 M, and NaCl solutions were incorporated to mimic a physiological environment. The system was initially equilibrated using an *NVT* ensemble for 10 ns to stabilize the protein–ligand complexes.^50–52^ After the previous step, a brief equilibration and minimization phase was performed using an *NPT* ensemble for 12 ns. The *NPT* ensemble was configured using the Nose–Hoover chain coupling method, incorporating varying temperatures, a relaxation time of 1.0 ps, and a consistent pressure of 1 bar throughout all the simulations.^50–52^ A time step of 2 fs was implemented. For pressure control, the Martyna–Tuckerman–Klein chain coupling scheme barostat method was utilized, featuring a relaxation time of 2 ps. The particle mesh Ewald method was employed to calculate long-range electrostatic interactions, with the radius for the Coulomb interactions set at 9 Å. The final production run lasted for 100 ns. To assess the stability of the MD simulations, we calculated the root mean square deviation (RMSD), the radius of gyration (*R*_g_), root mean square fluctuation (RMSF), as well as the number of hydrogen bonds (H-bonds), salt bridges, and solvent-accessible surface area (SASA).^50–52^

## Data availability

The data used for the manuscript entitled “*In vitro* and *in silico* analysis of synthesized *N*-benzyl indole-derived hydrazones as potential anti-triple negative breast cancer agents” are included in the ESI file,† which is available online on the RSC Advances website.

## Author contributions

Conceptualization: Z. S. and A. A.-H. Investigation: U. F., U. G., J. H. Formal Analysis: F. K., A. K., W. U. I., methodology, software: S. N. M., S. Y. C., R. D. J. funding acquisition, Resources: H. A. A.-S., A. M. E. Writing original draft: U. G., Z. S.

## Conflicts of interest

The authors have declared no conflict of interest.

## Supplementary Material

RA-015-D5RA02194D-s001
