# Supplementary material for: In vitro and in silico analysis of synthesized N-benzyl indole-derived hydrazones as potential anti-triple negative breast cancer agents
Source: RSC Adv. 2025 Apr 25;15(17):13284–99. doi: 10.1039/d5ra02194d (PMC12022751; doi:10.1039/d5ra02194d)

Supplementary Information (SI) for RSC Advances.
This journal is © The Royal Society of Chemistry 2025

1HNMR of 5a

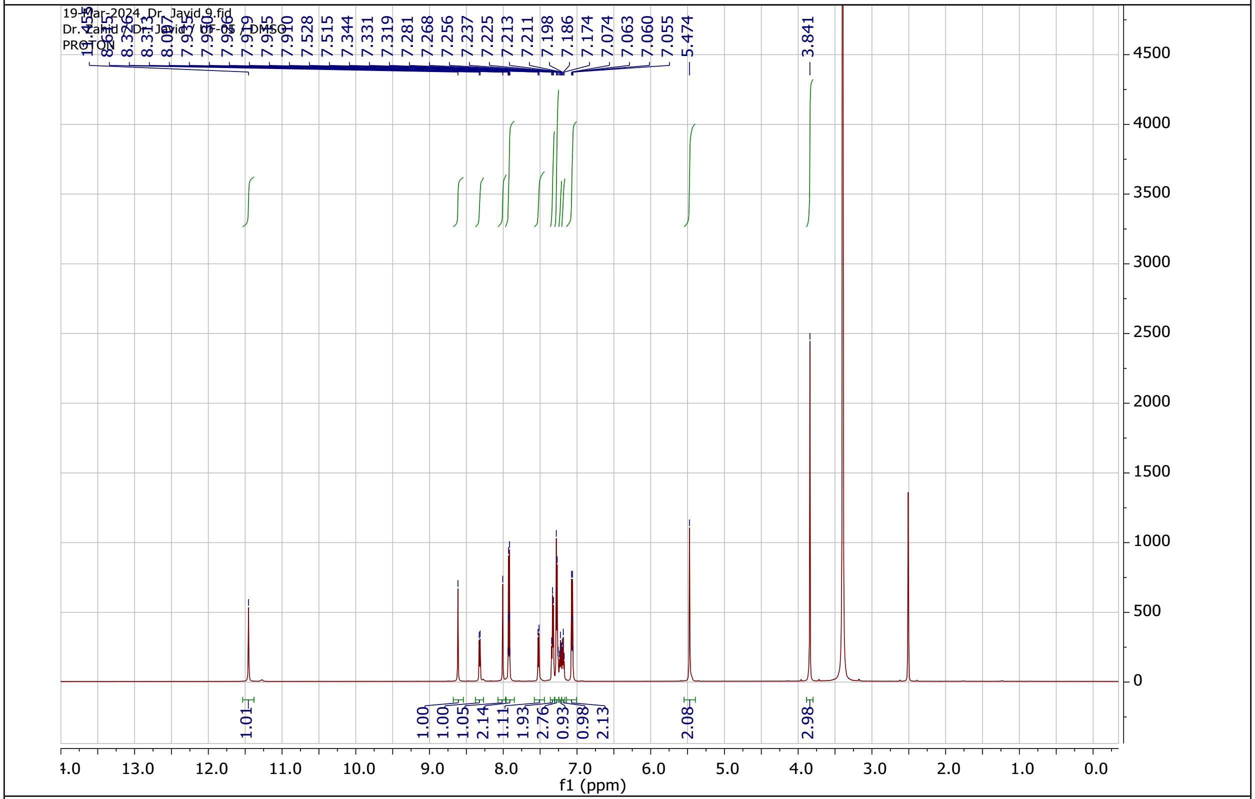

13CNMR of 5a

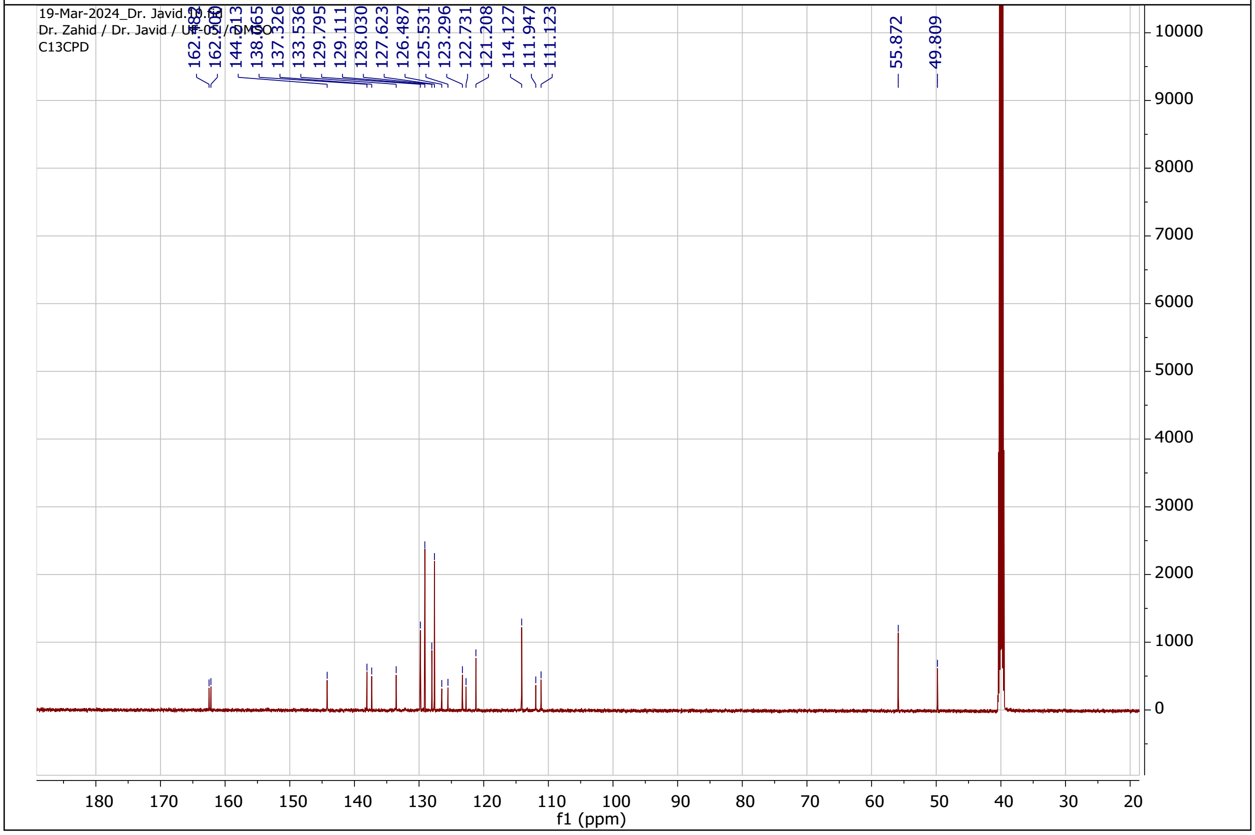

1HNMR of 5b

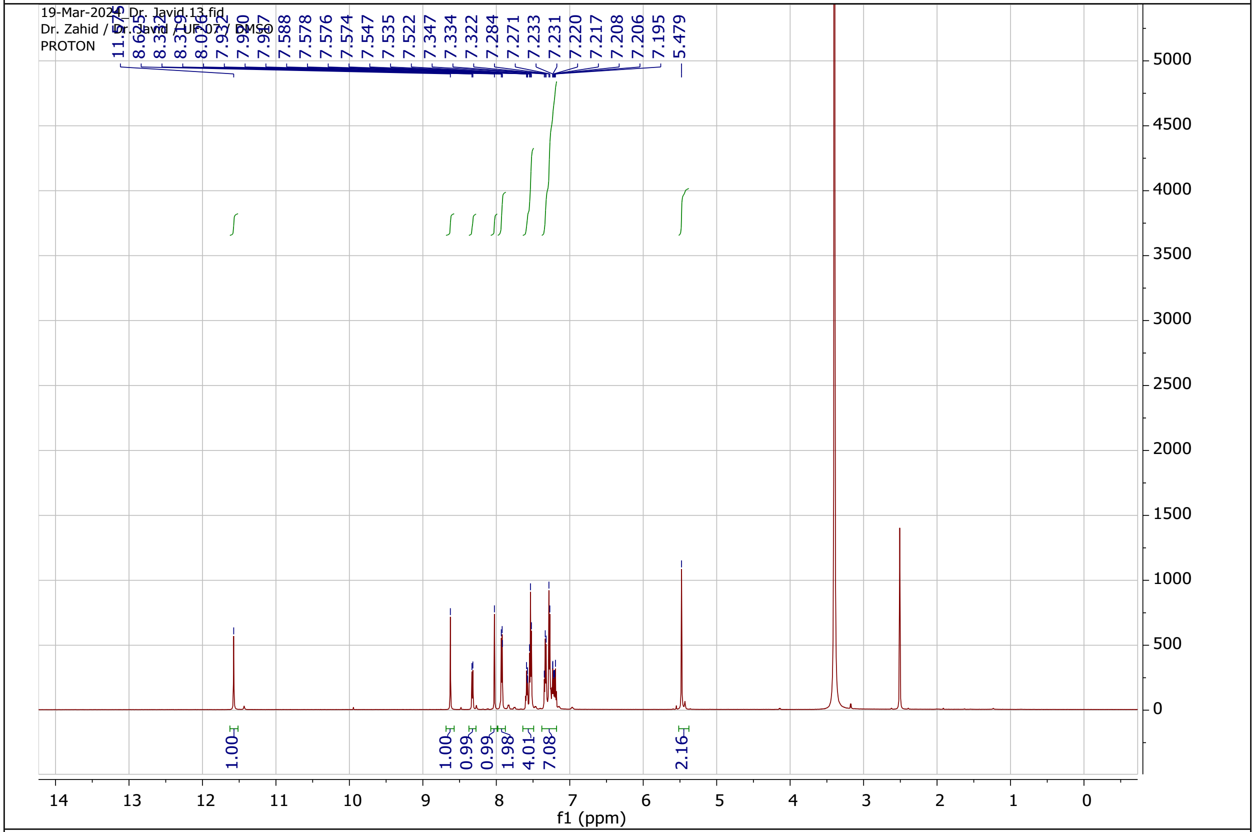

13CNMR of 5b

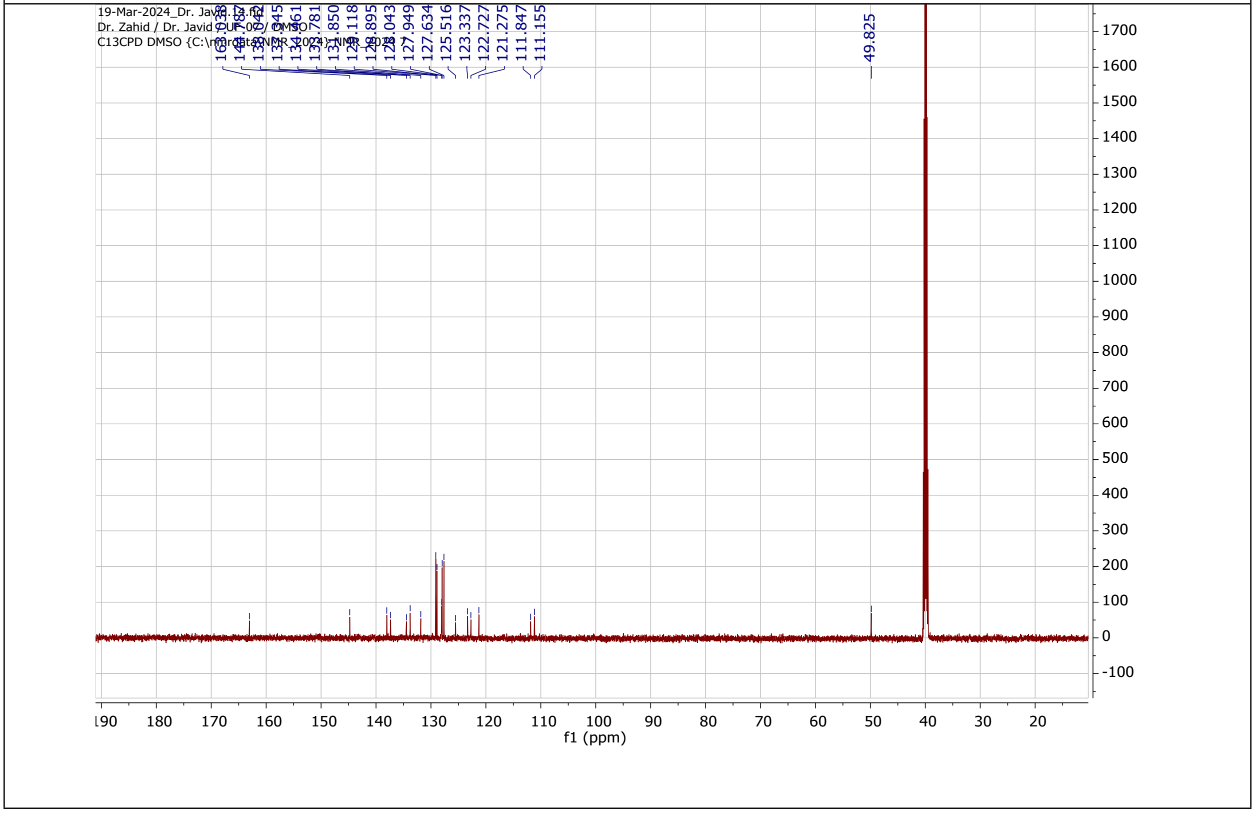

1HNMR of 5c

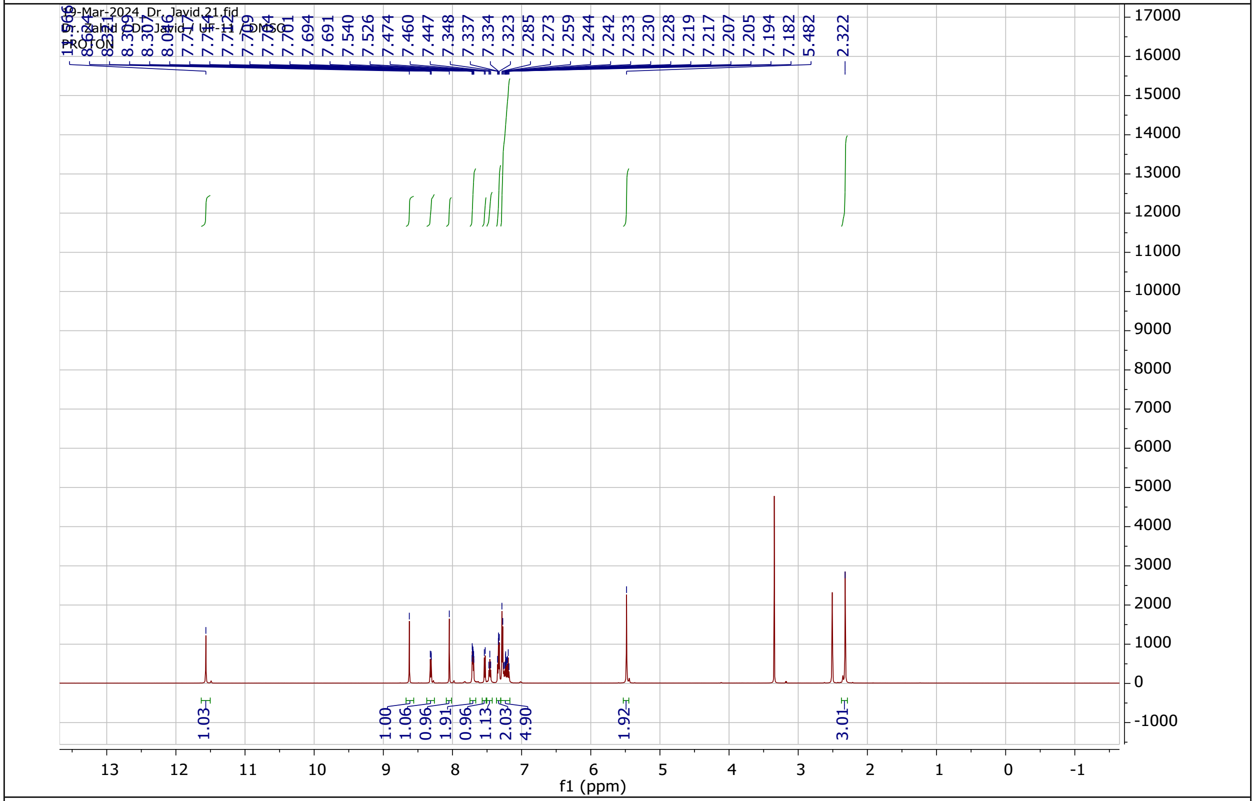

13CNMR of 5c

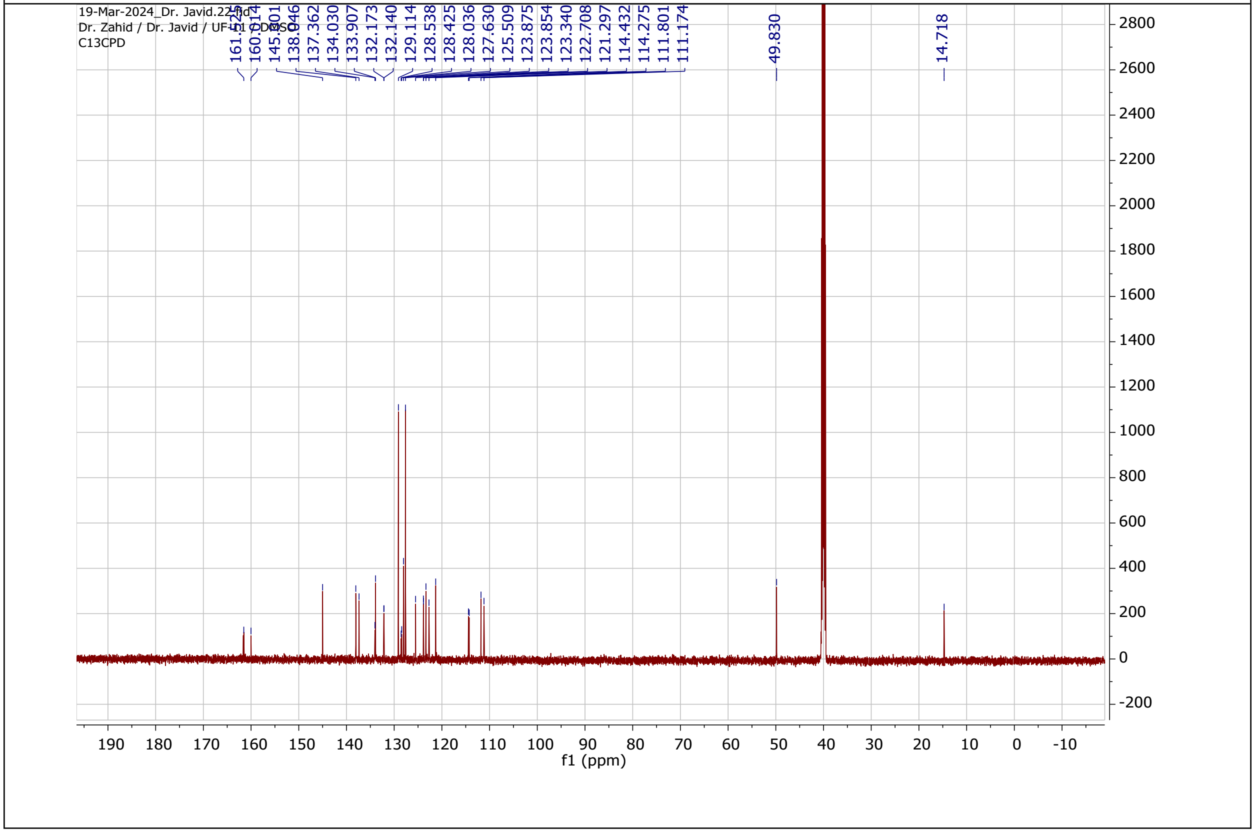

1HNMR of 5d

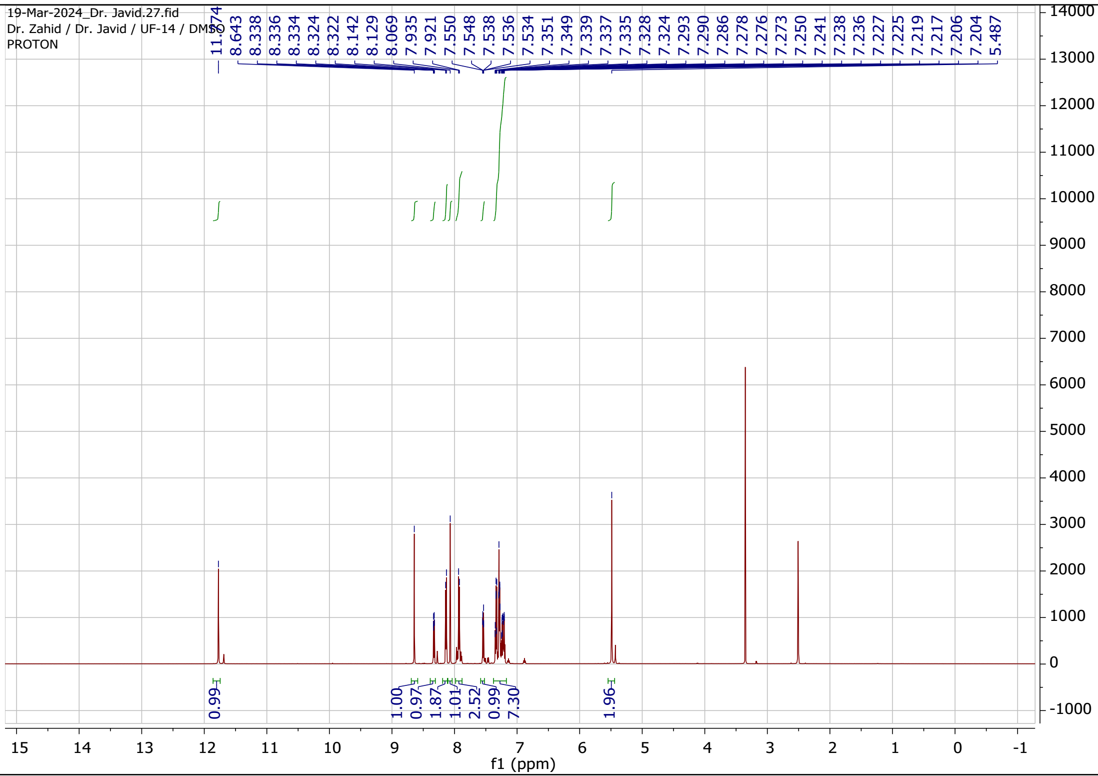

13CNMR of 5d

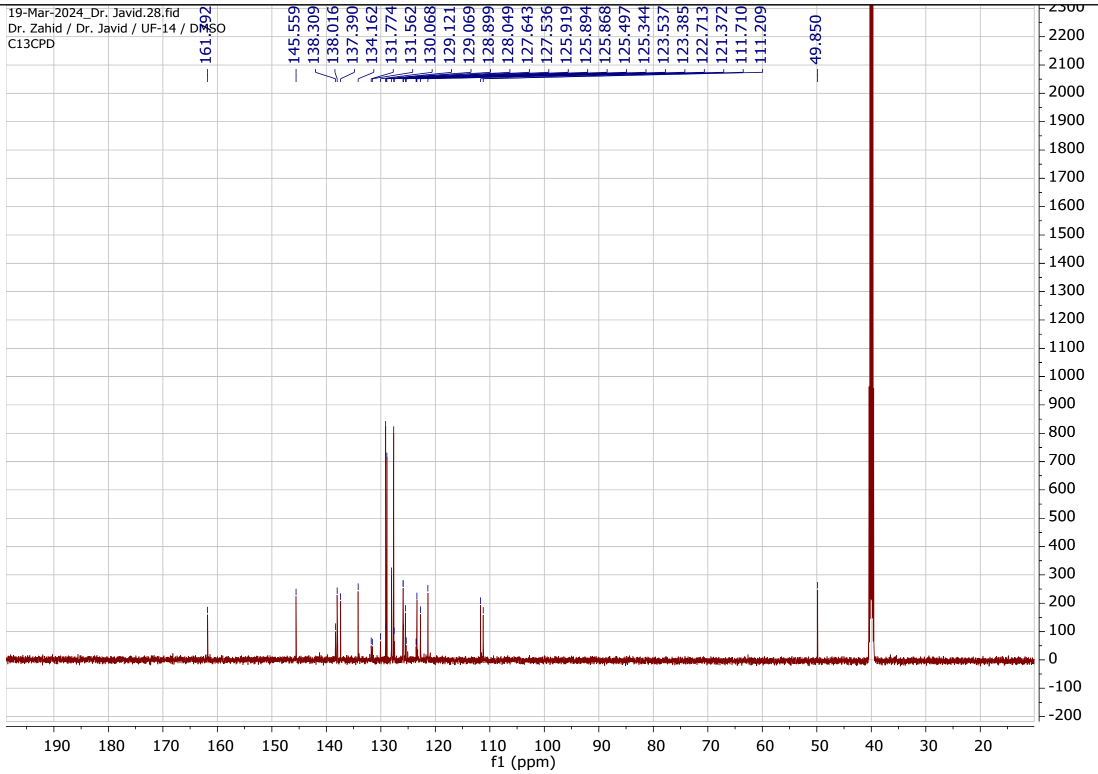

1HNMR of 5e

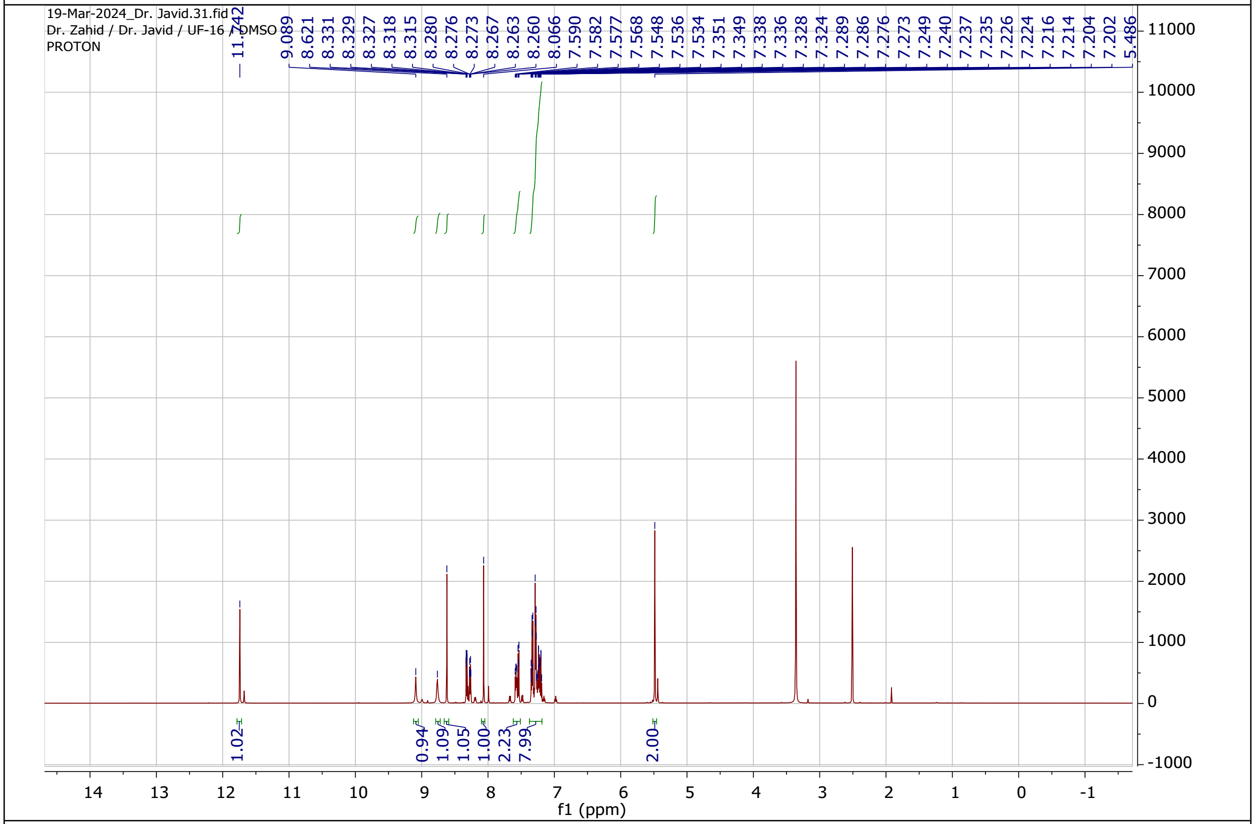

13CNMR of 5e

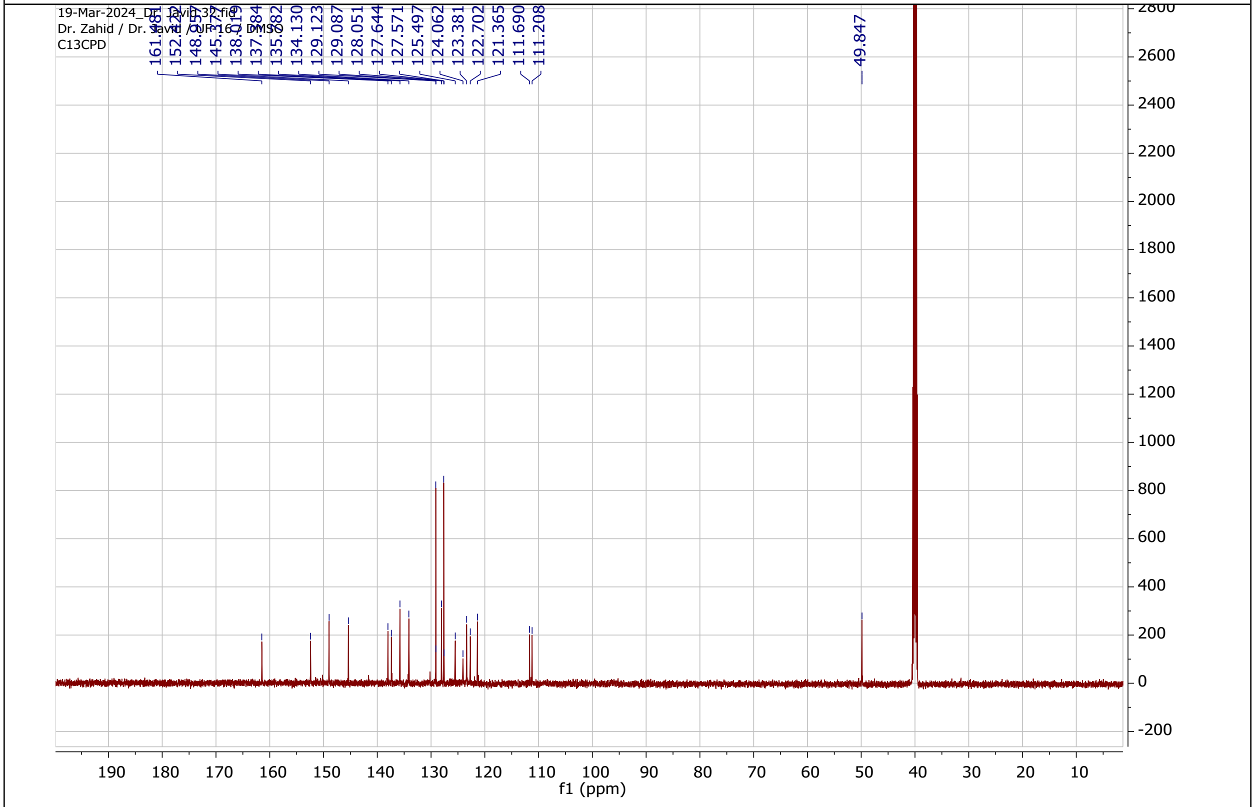

1HNMR of 5f

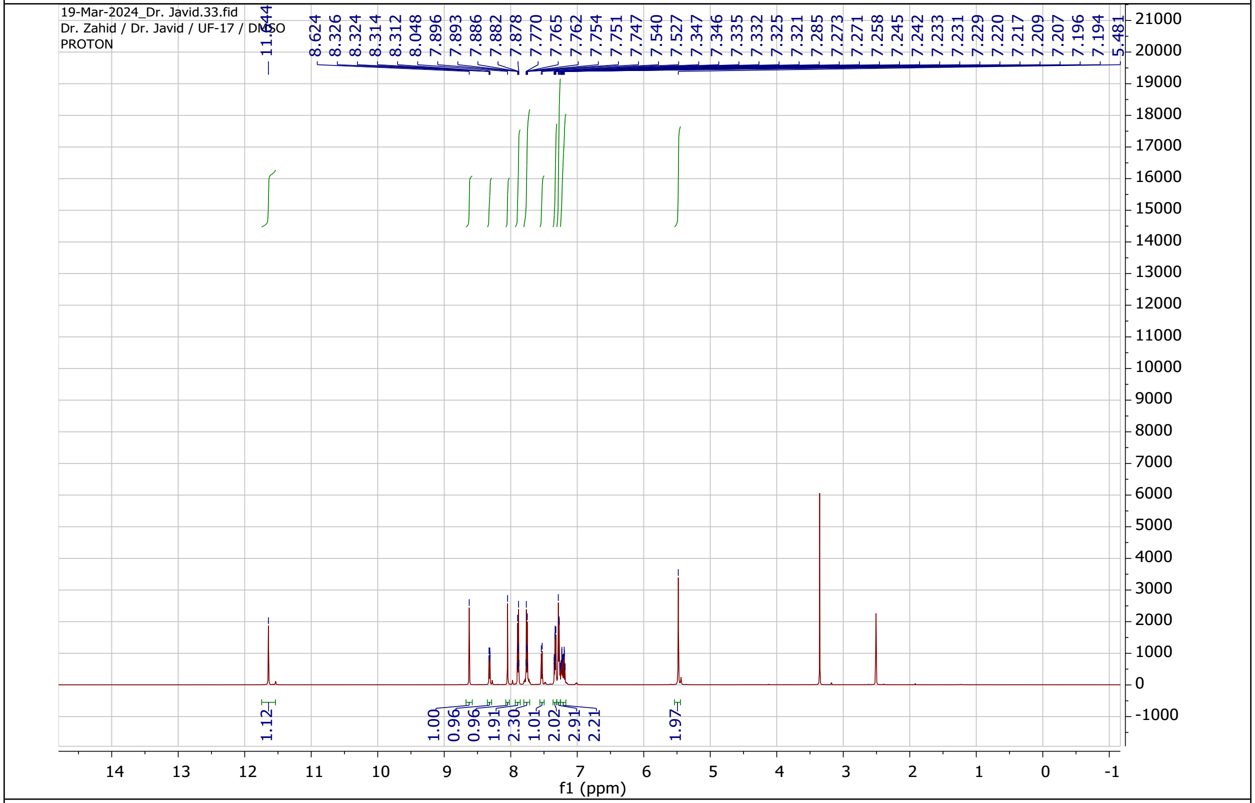

13CNMR of 5f

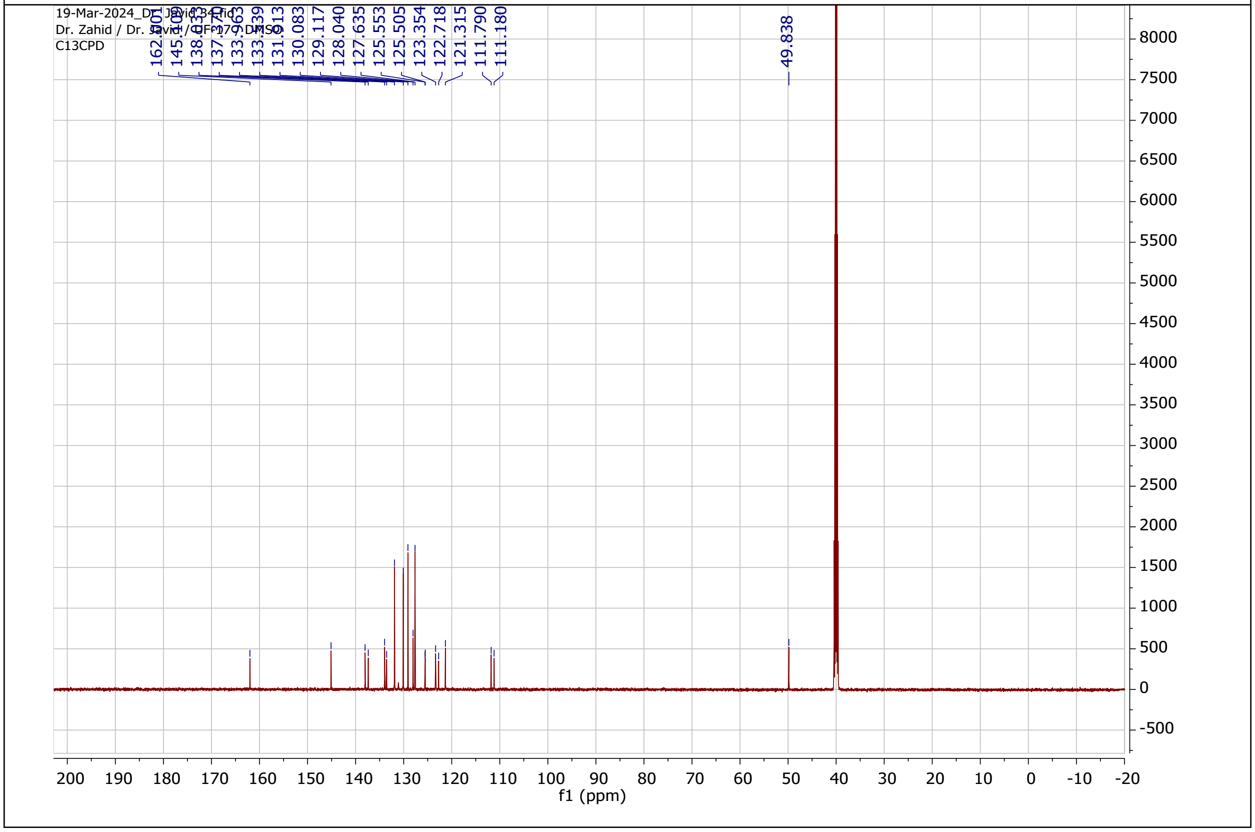

1HNMR of 5g

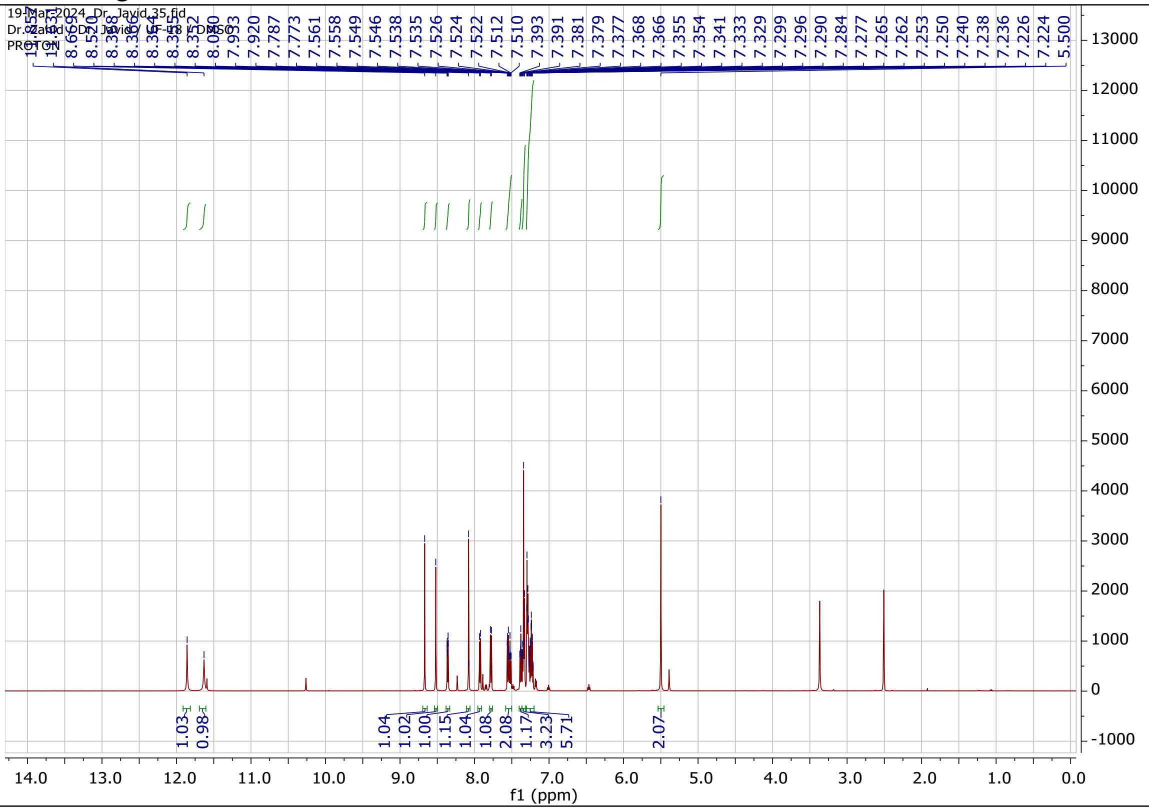

13CNMR of 5g

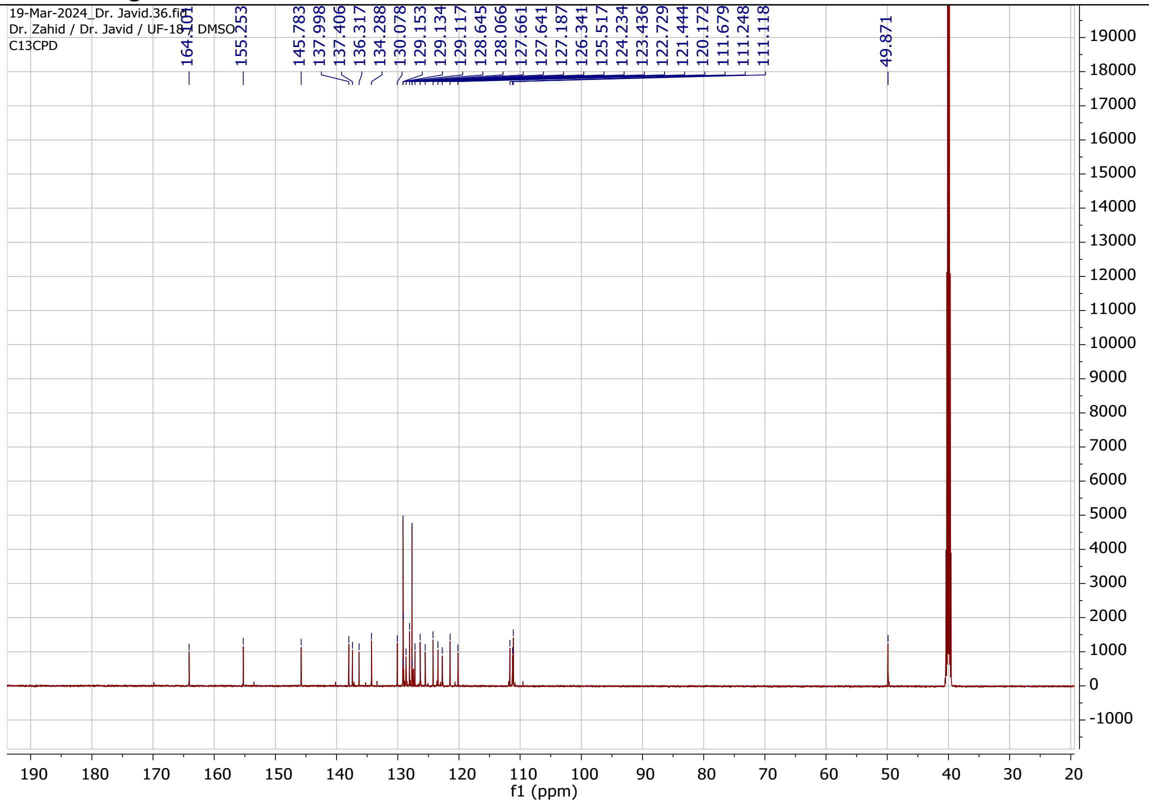

1HNMR of 5h

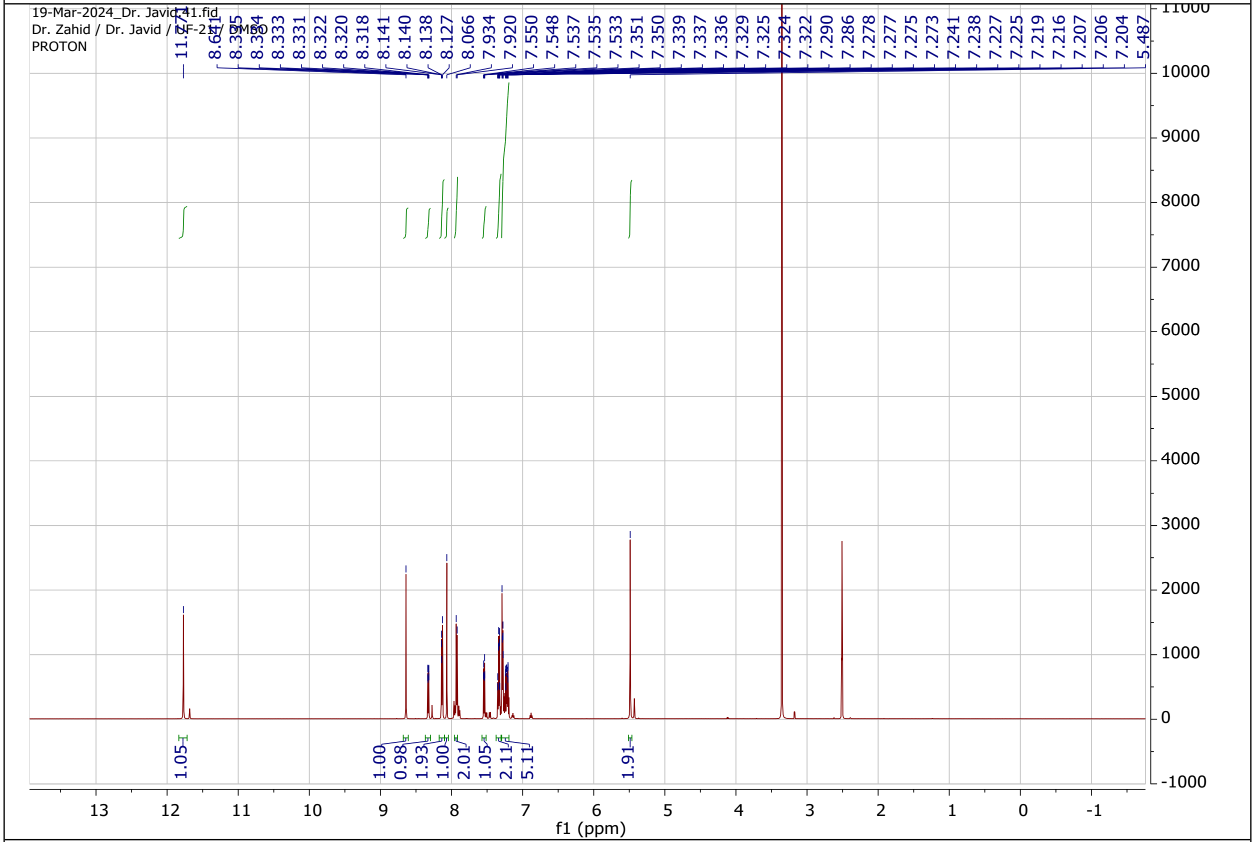

13CNMR of 5h

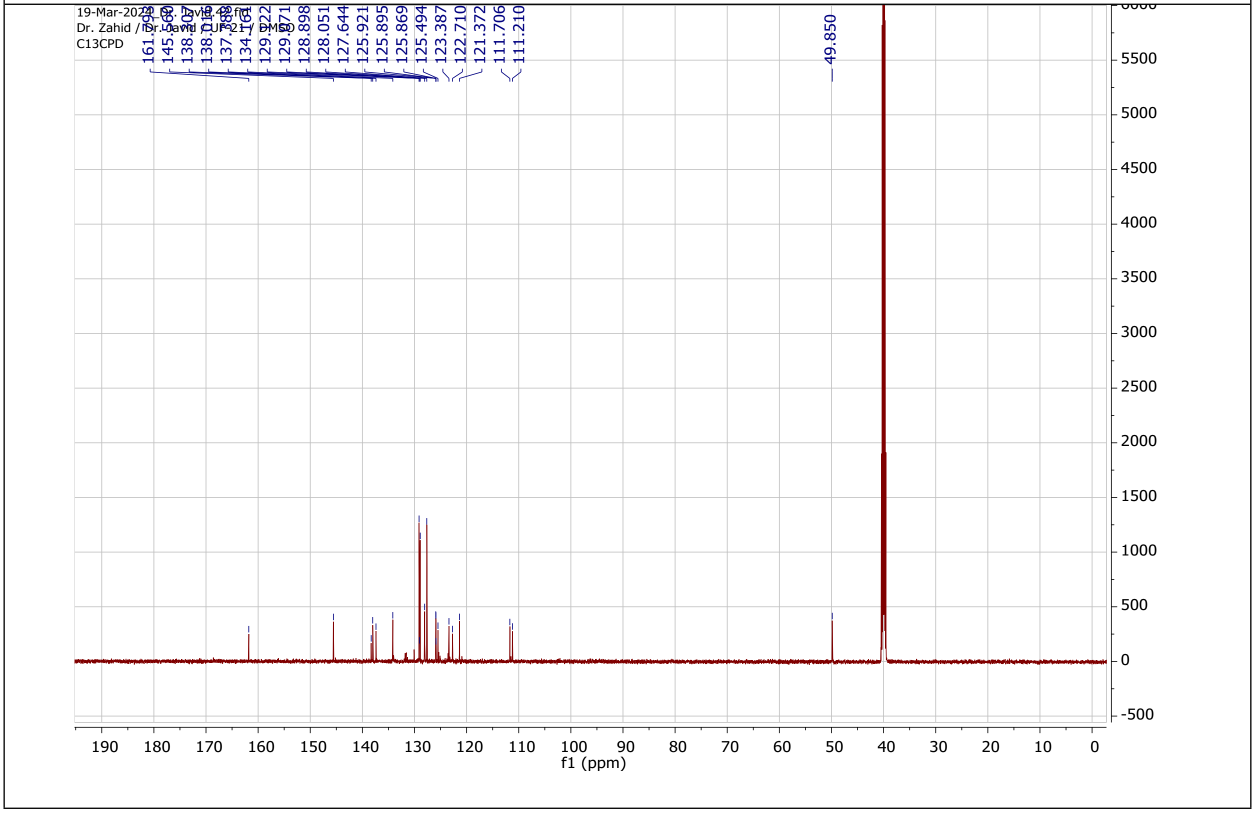

1HNMR of 5i

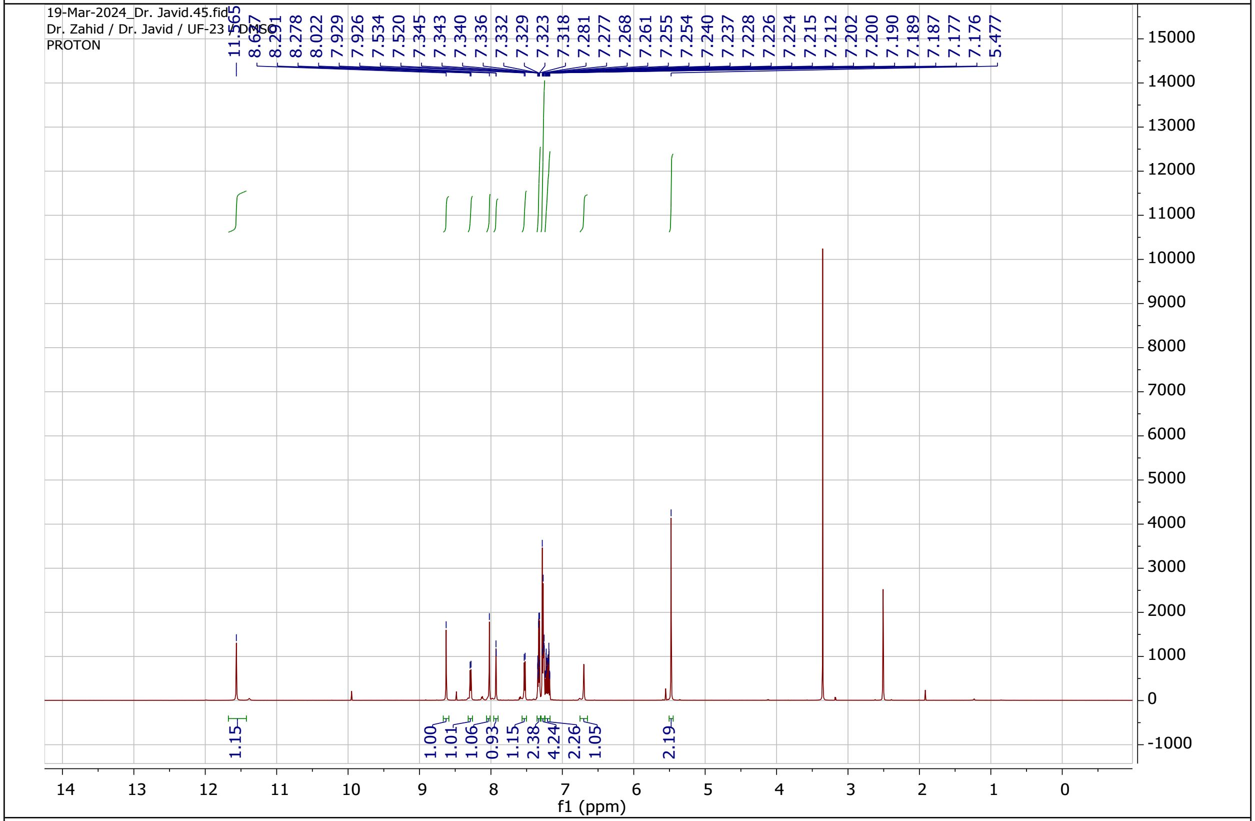

13CNMR of 5i

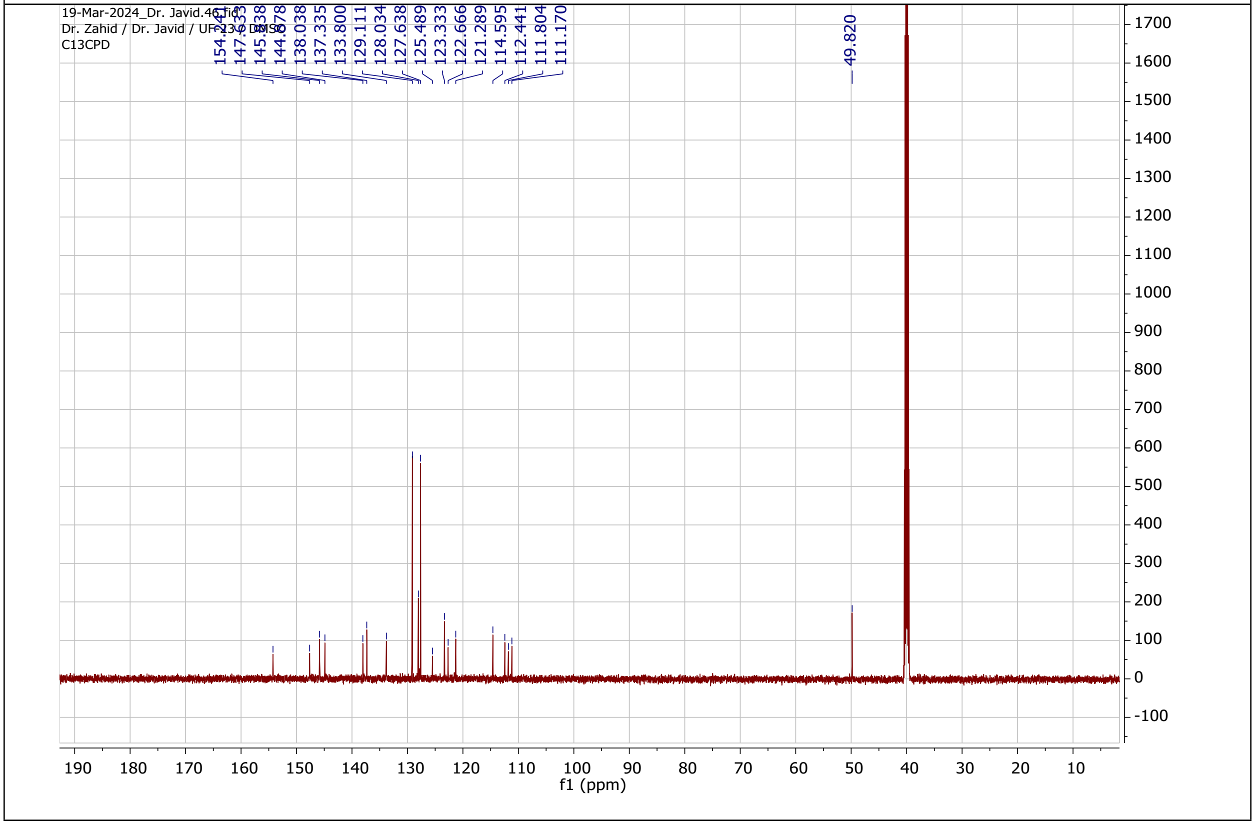

1HNMR of 5j

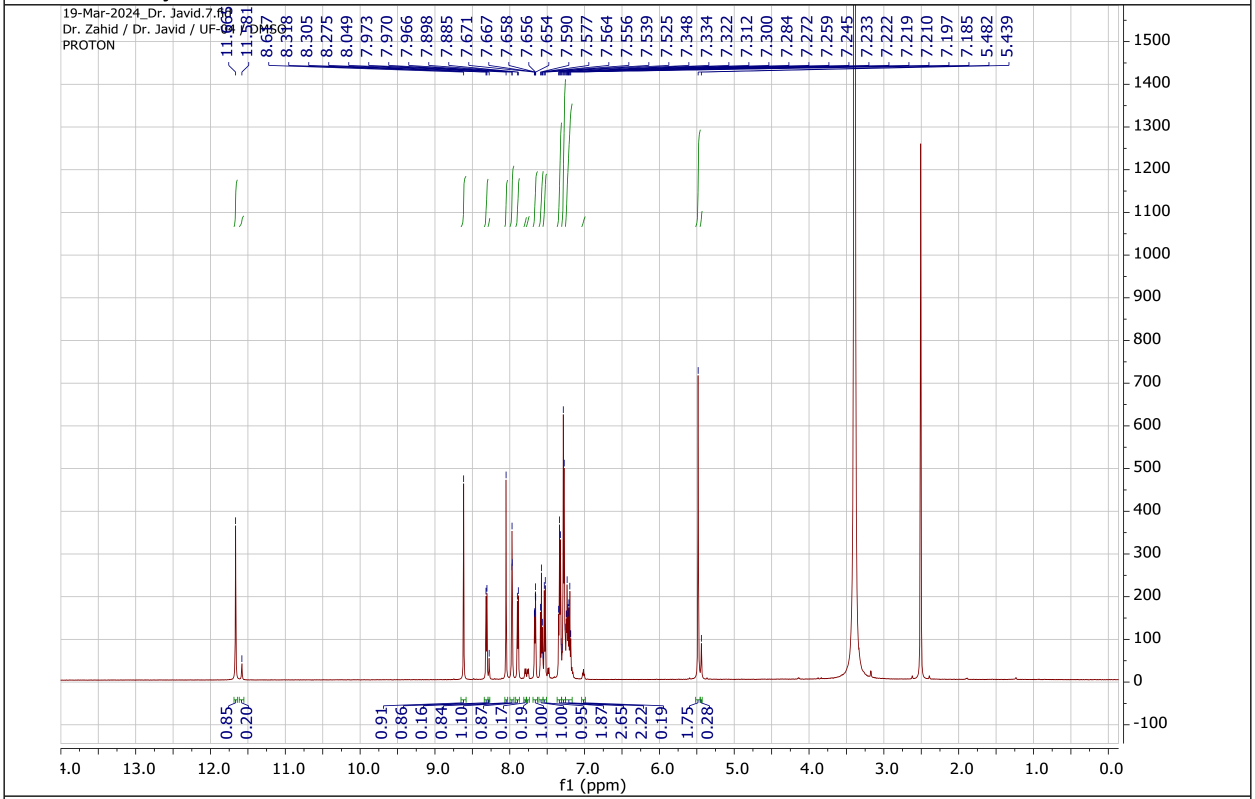

13CNMR of 5j

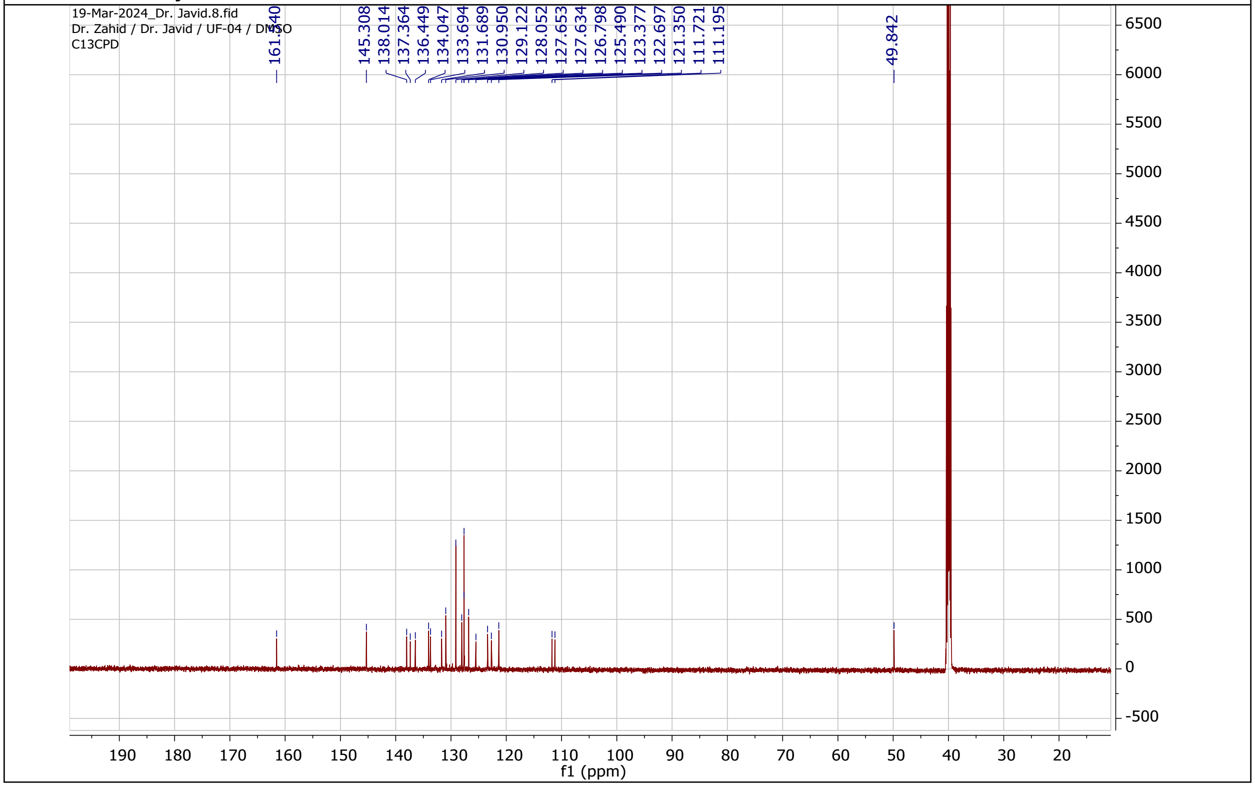

1HNMR of 5k

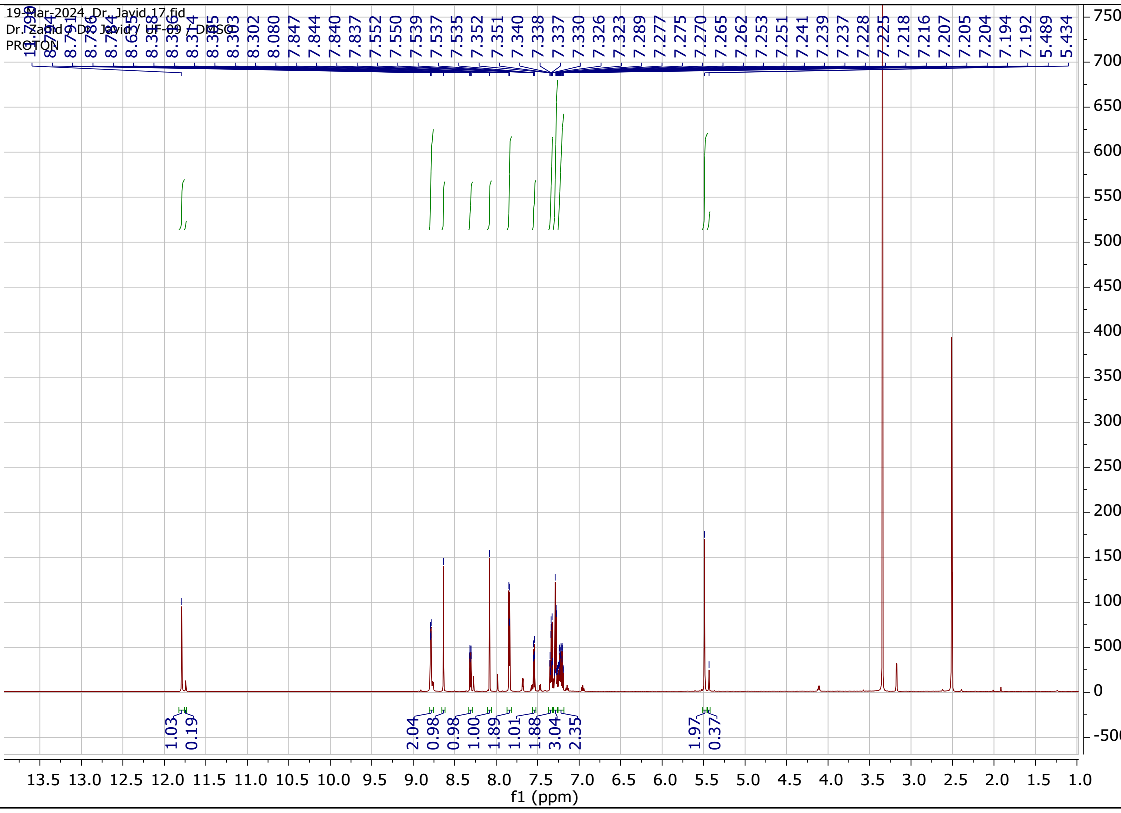

13CNMR of 5k

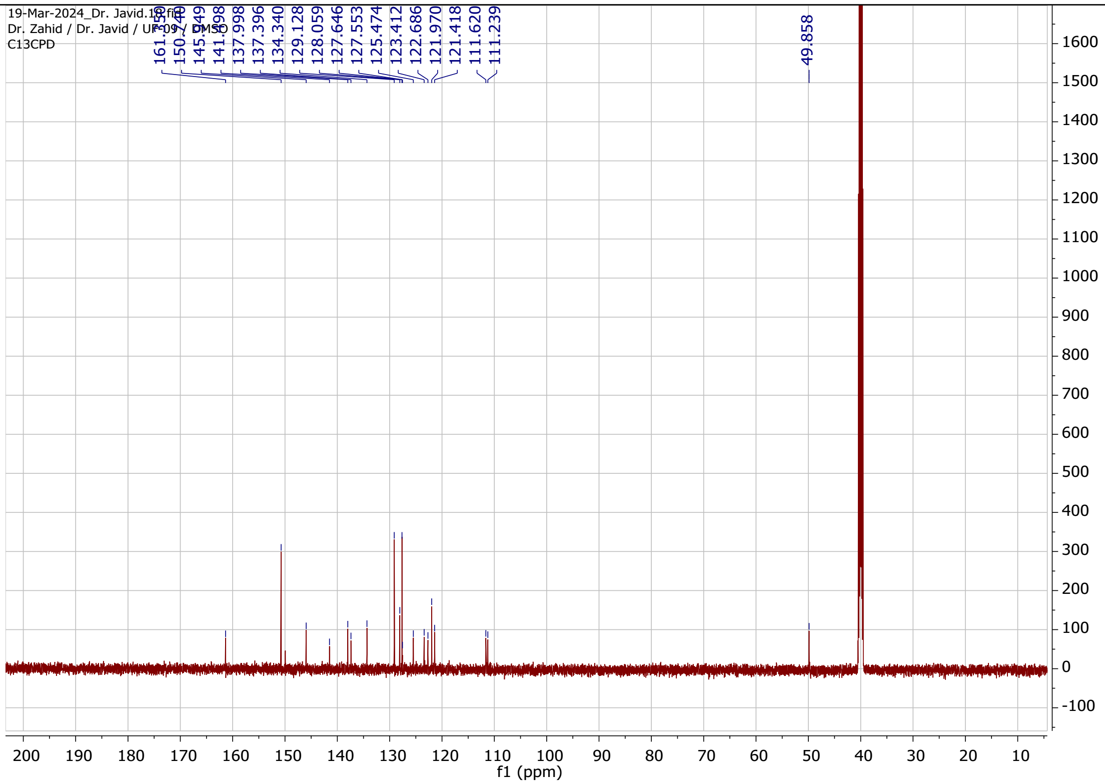

1HNMR of 5l

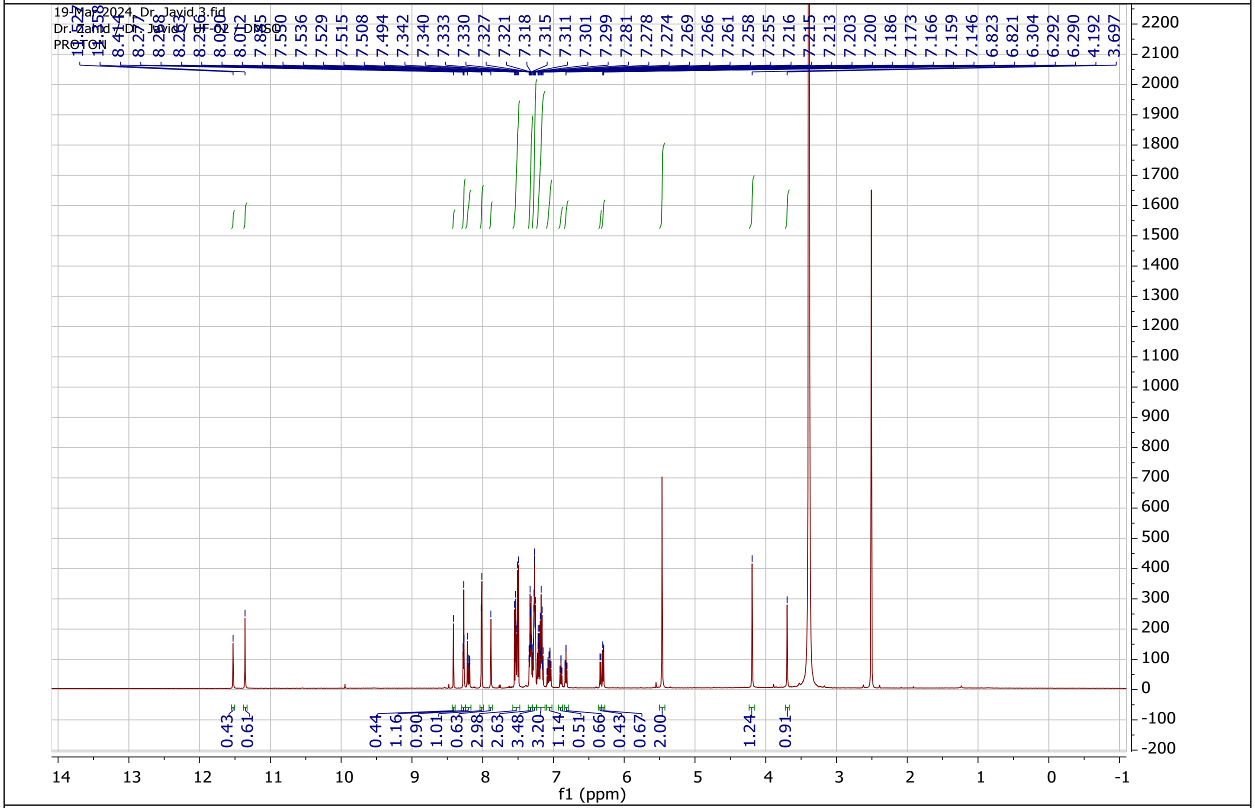

13CNMR of 5l

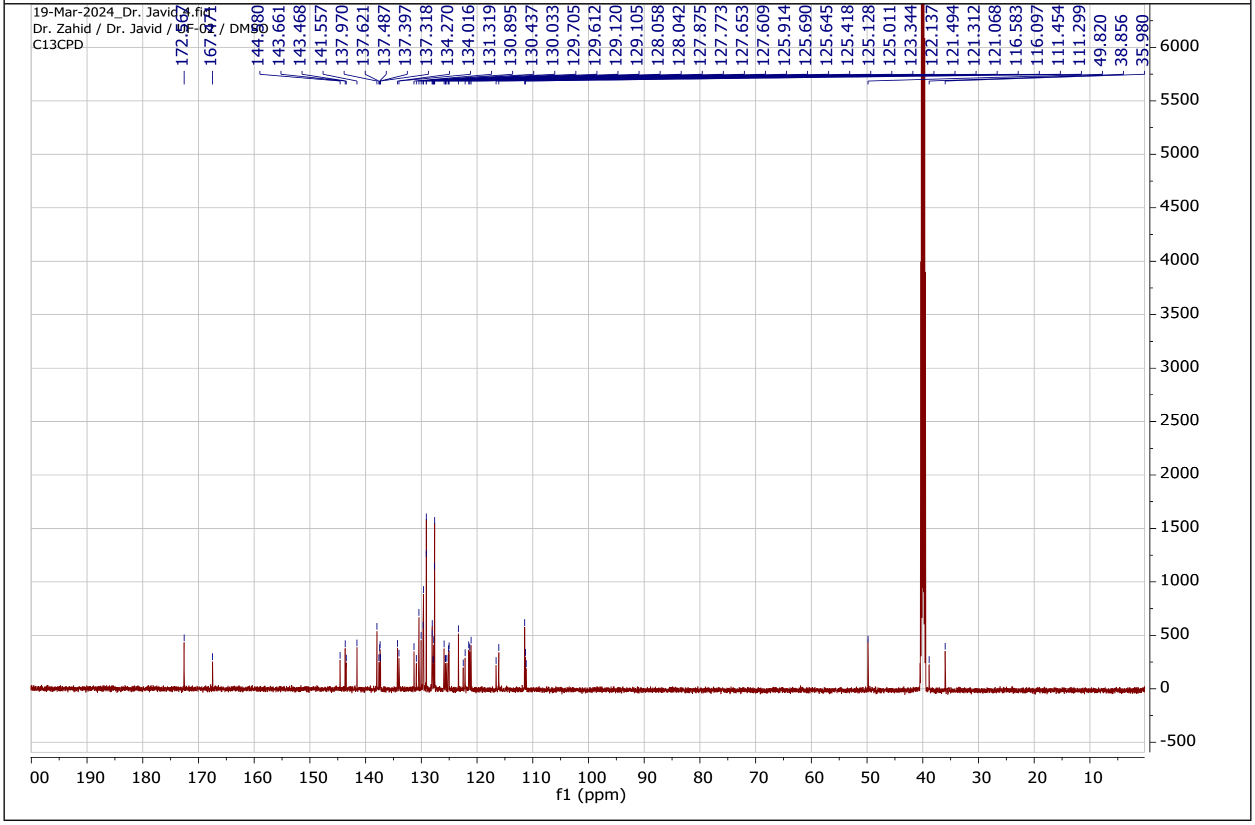

1HNMR of 5m

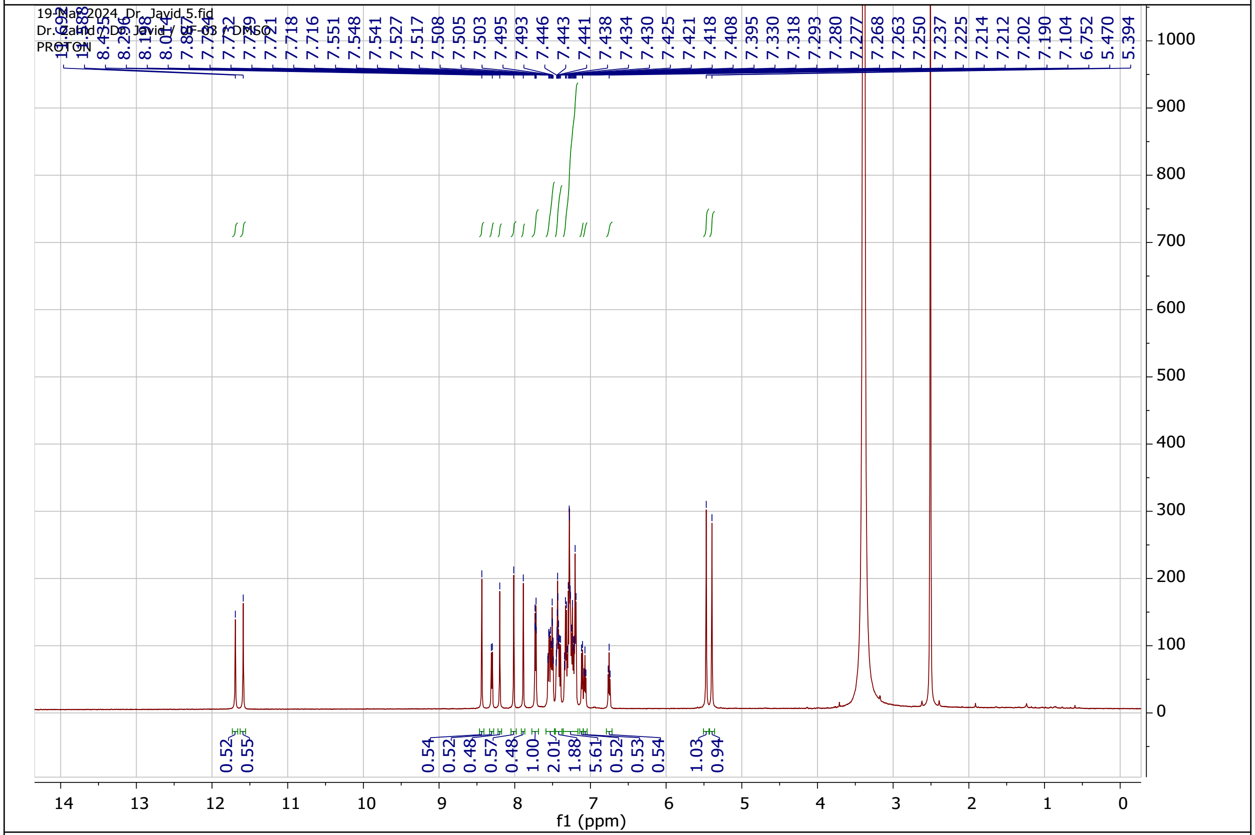

13CNMR of 5m

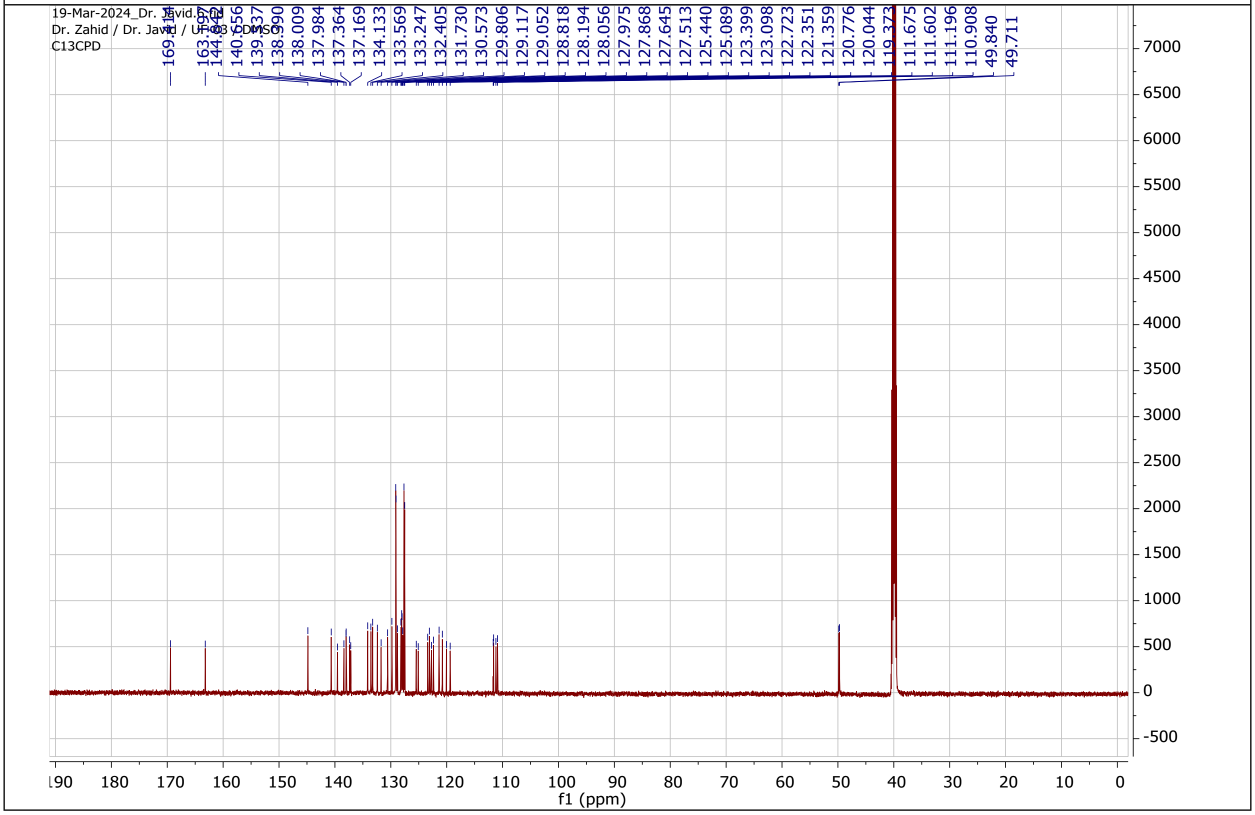

1HNMR of 5n

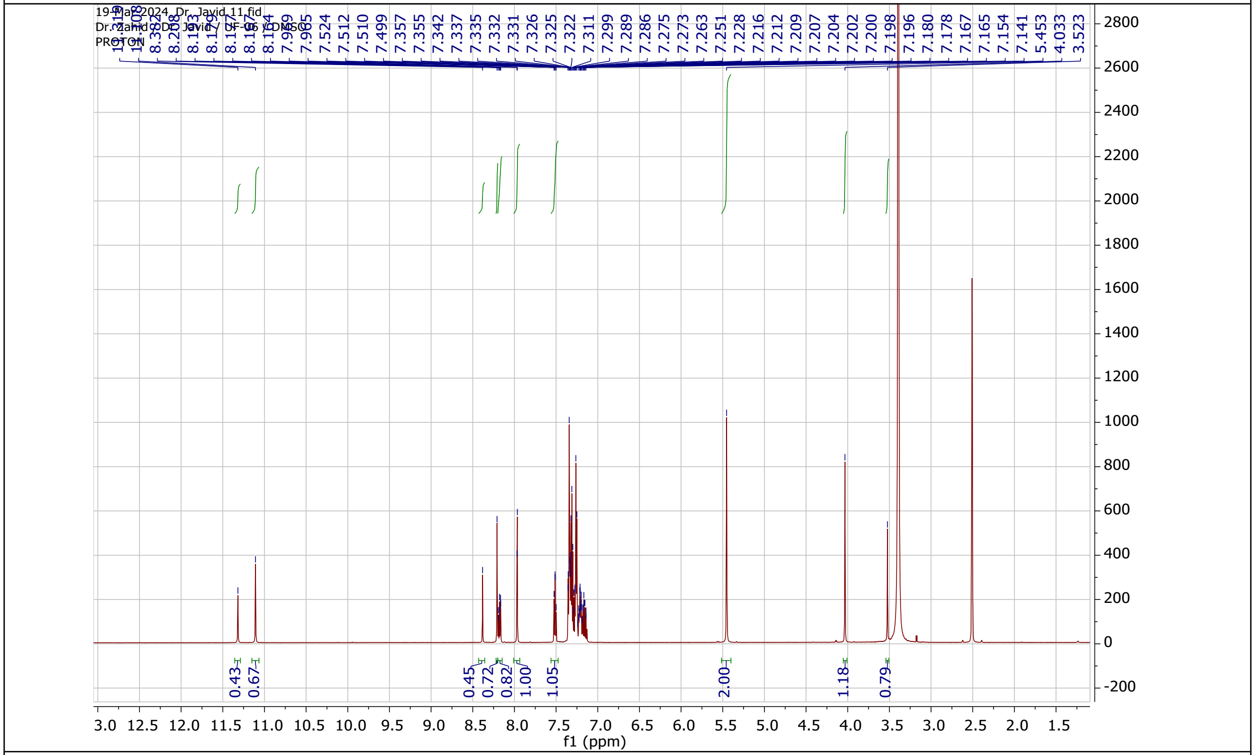

13CNMR of 5n

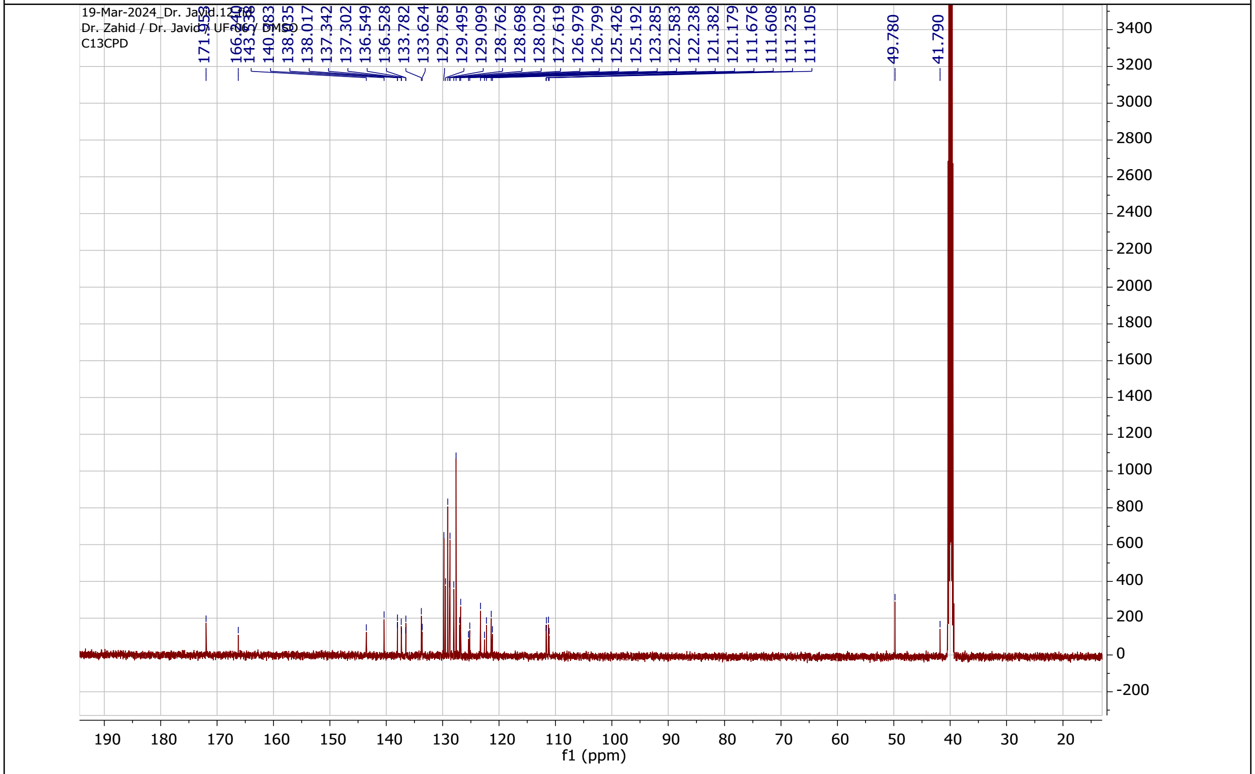

1HNMR of 5o

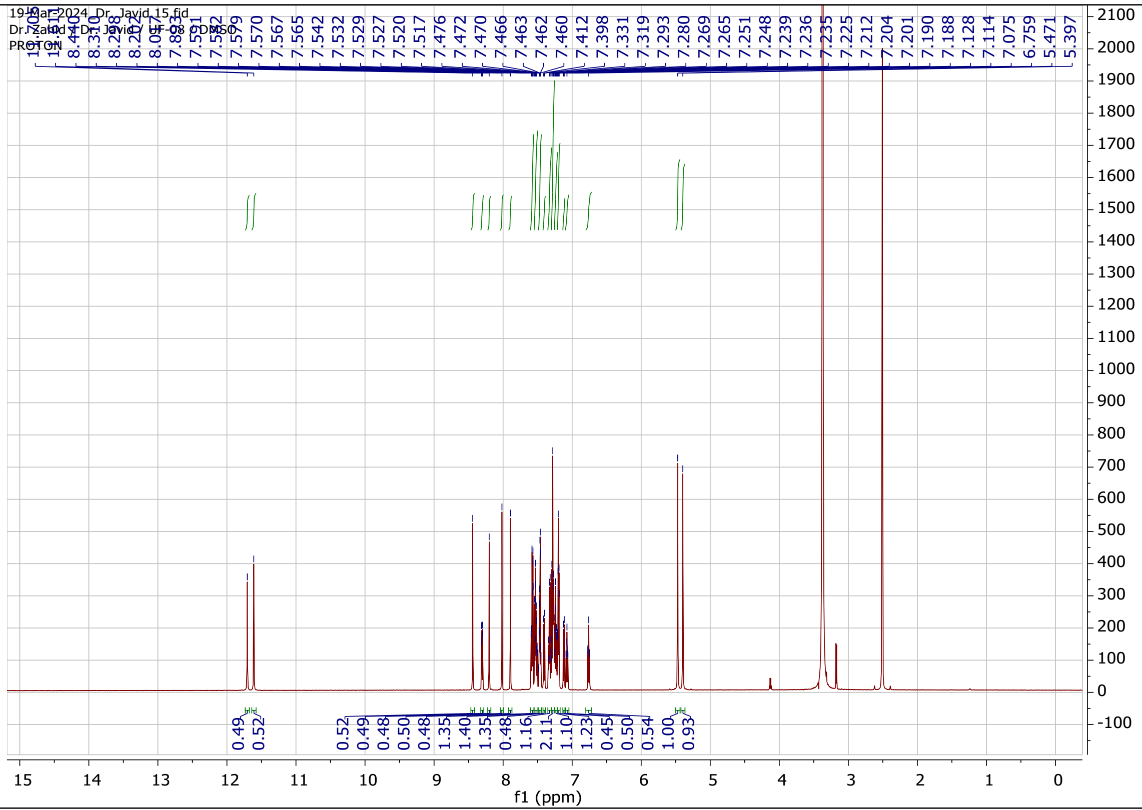

13CNMR of 5o

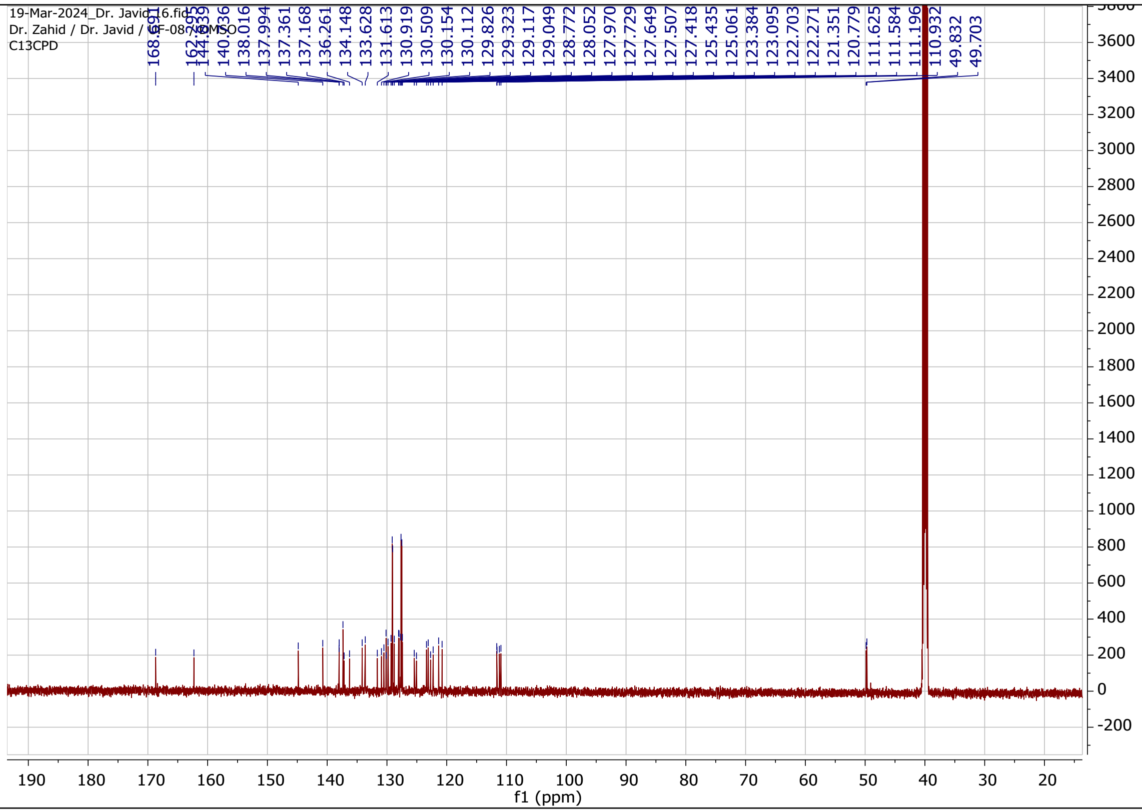

1HNMR of 5p

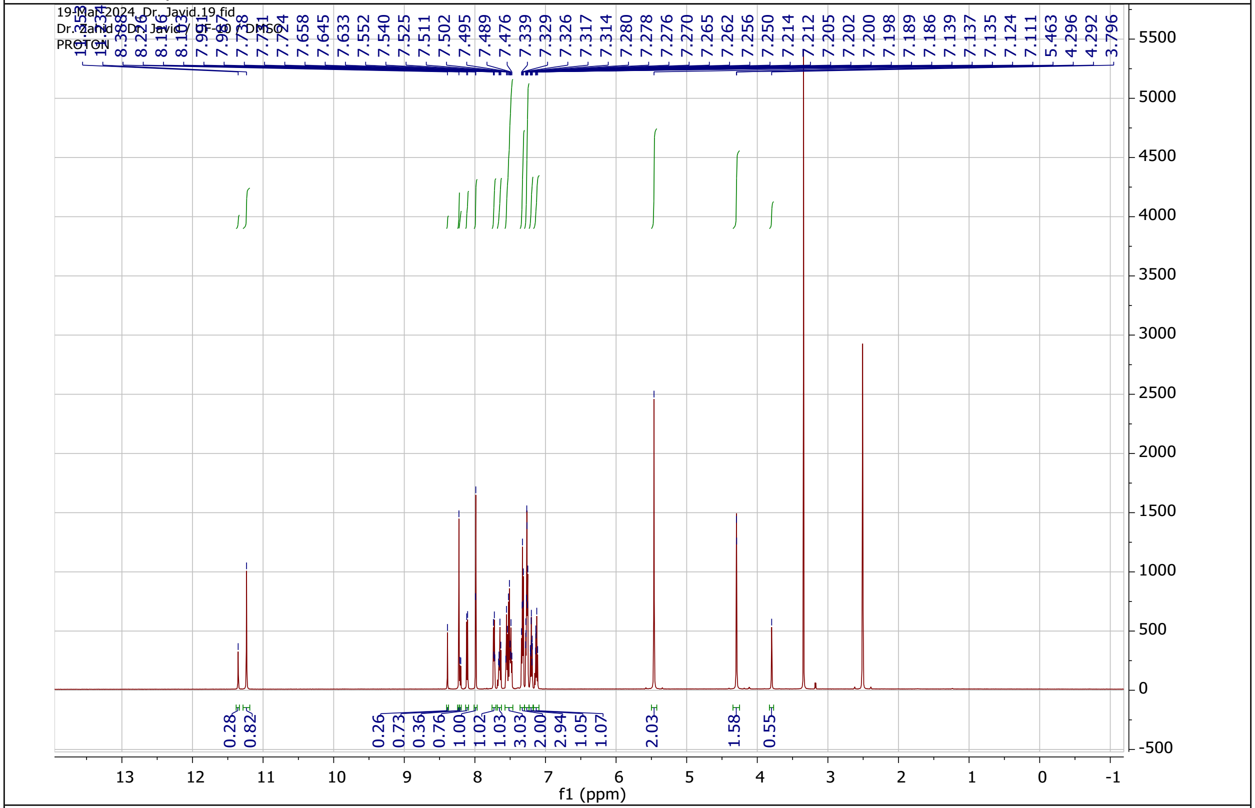

13CNMR of 5p

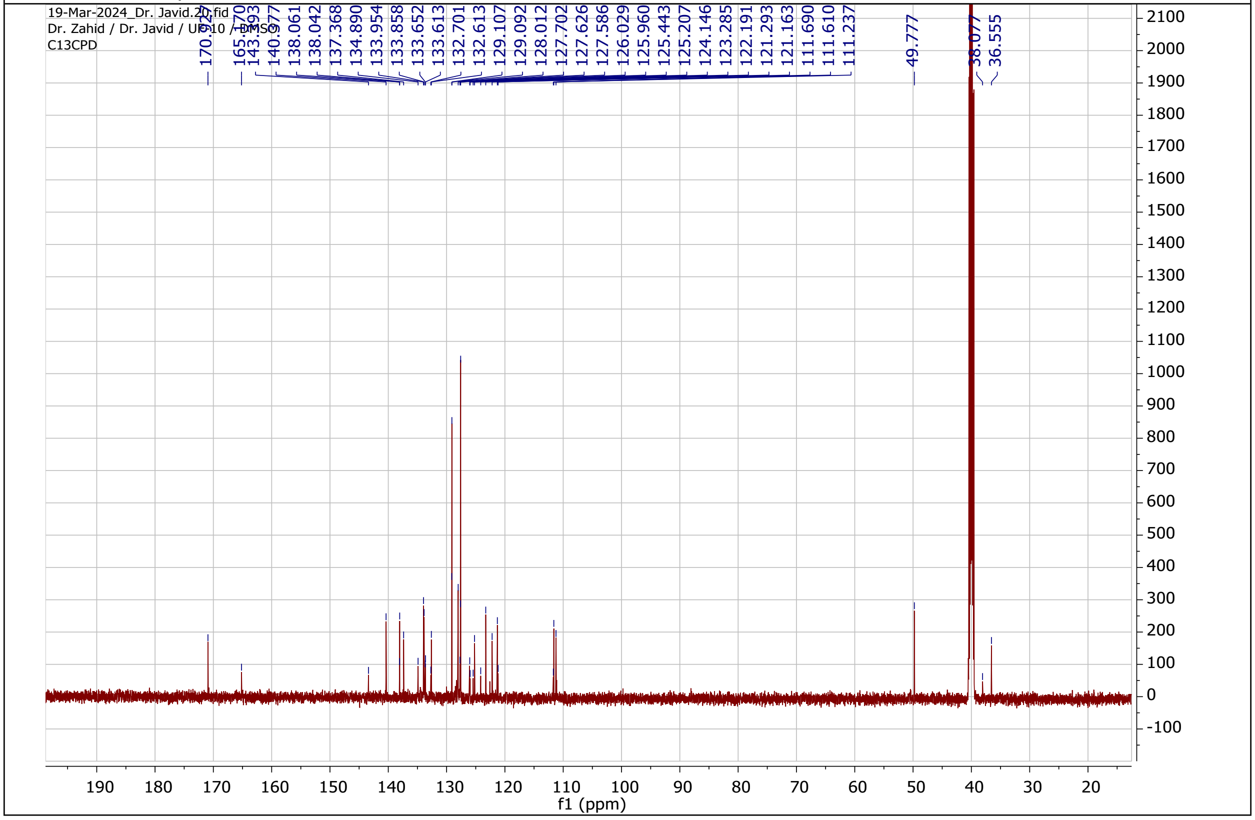

1HNMR of 5q

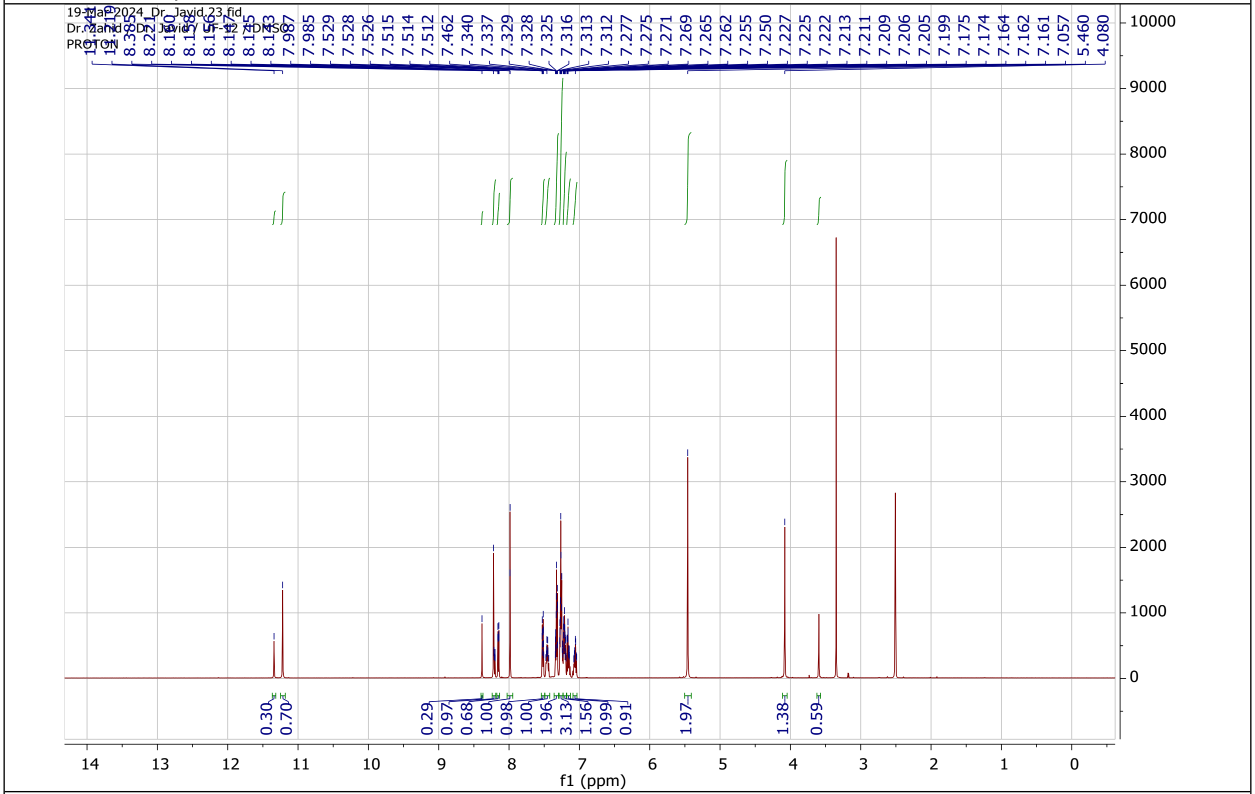

13CNMR of 5q

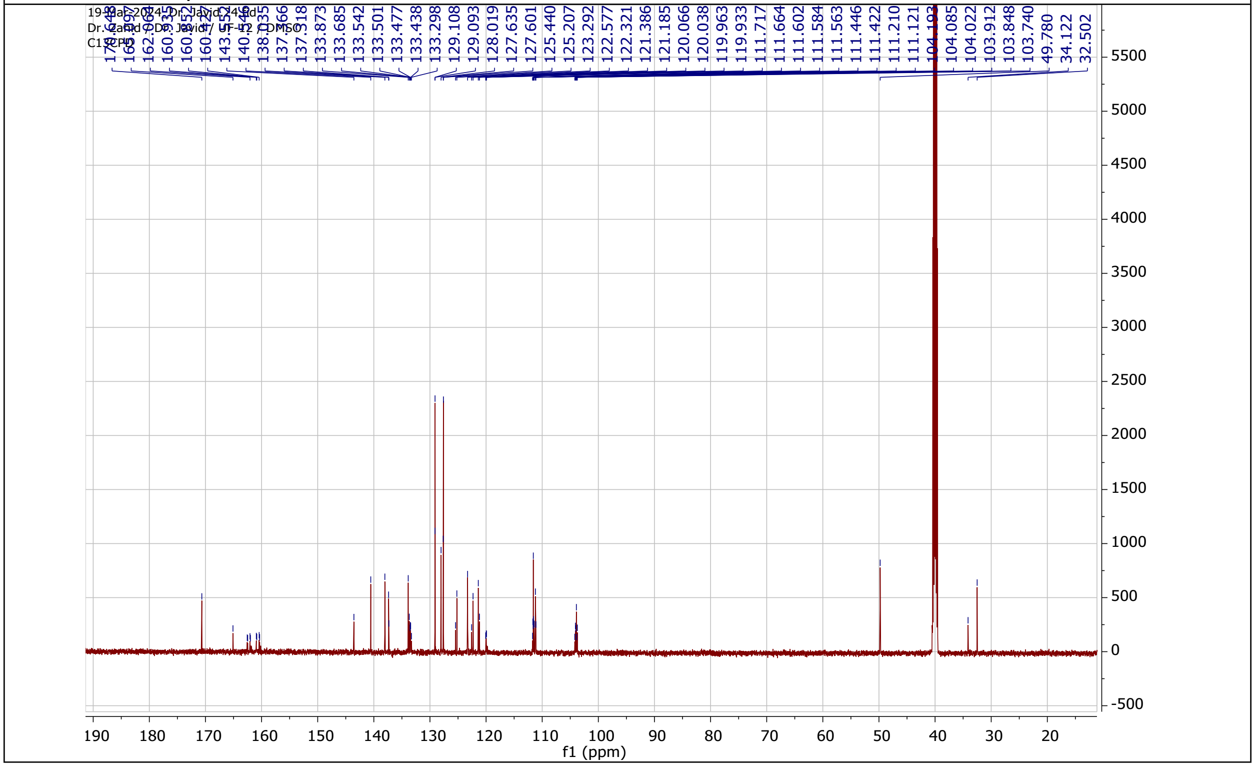

1HNMR of 5r

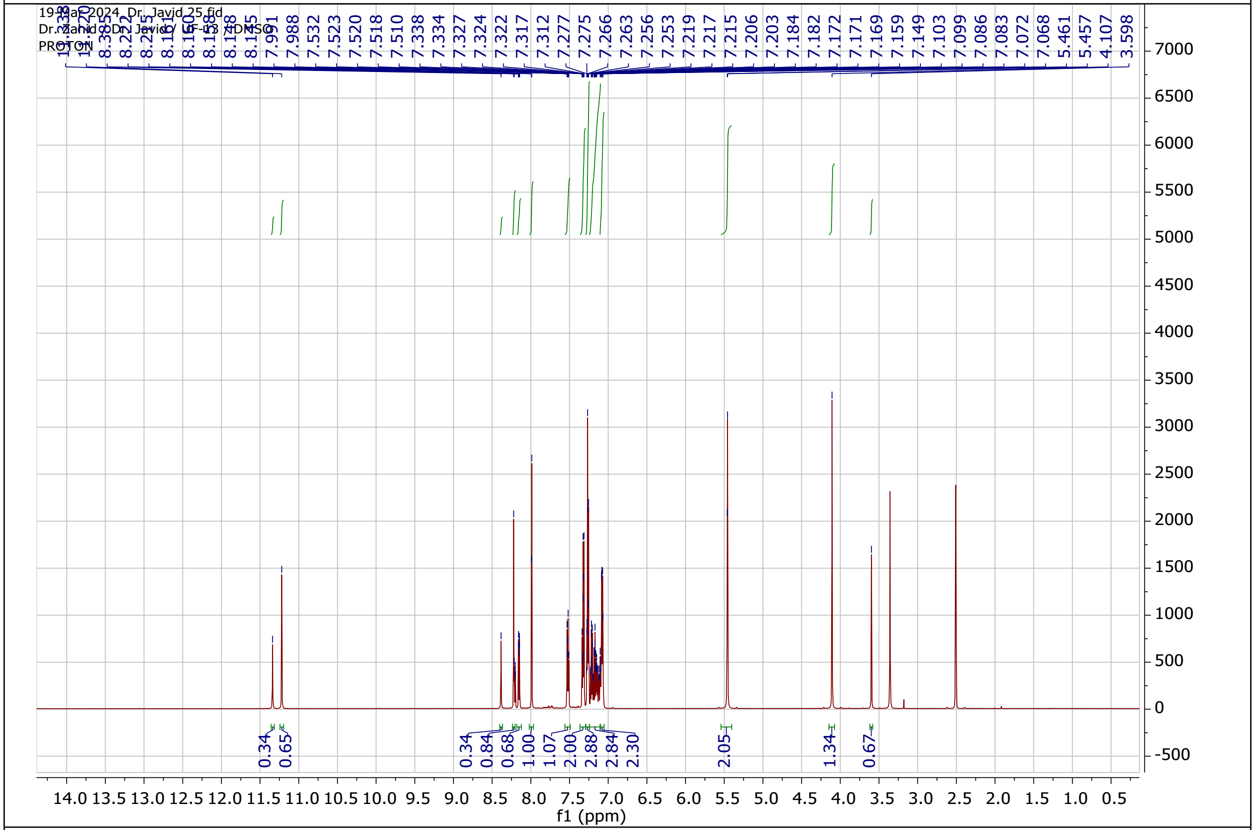

13CNMR of 5r

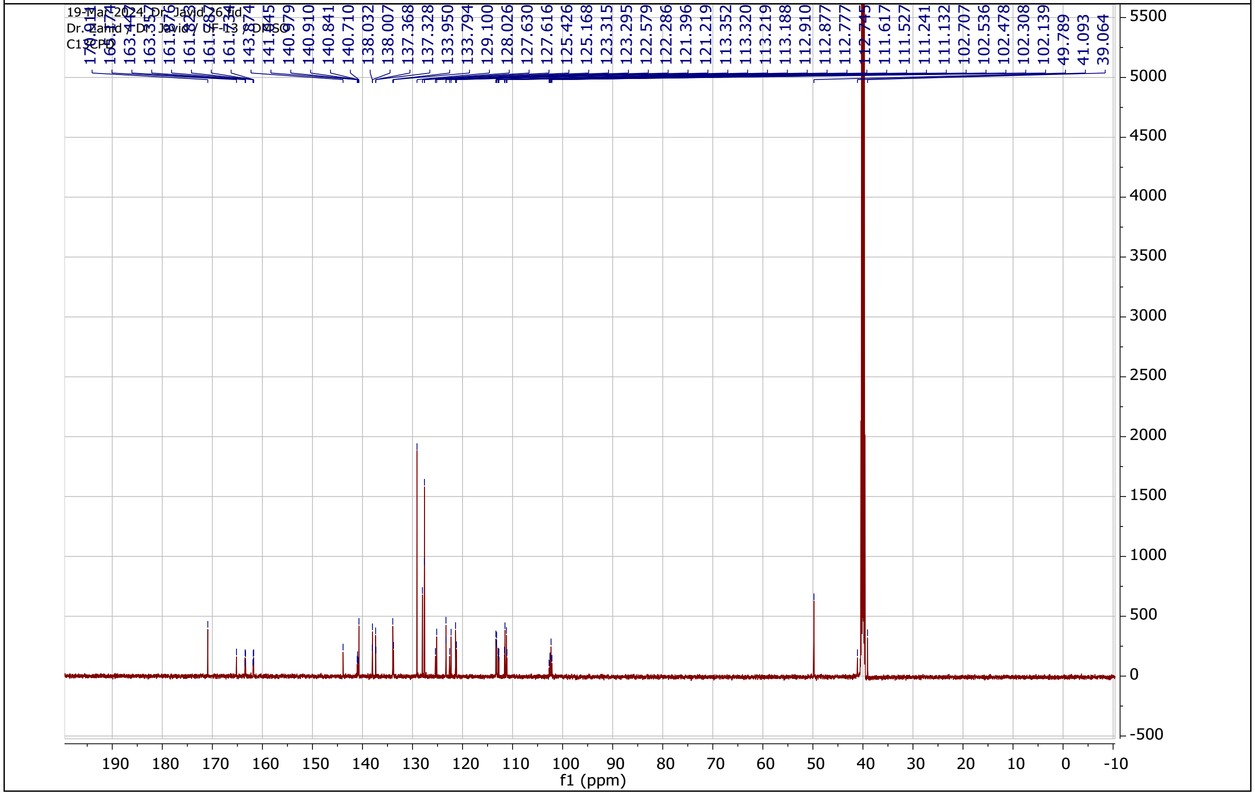

1HNMR of 5s

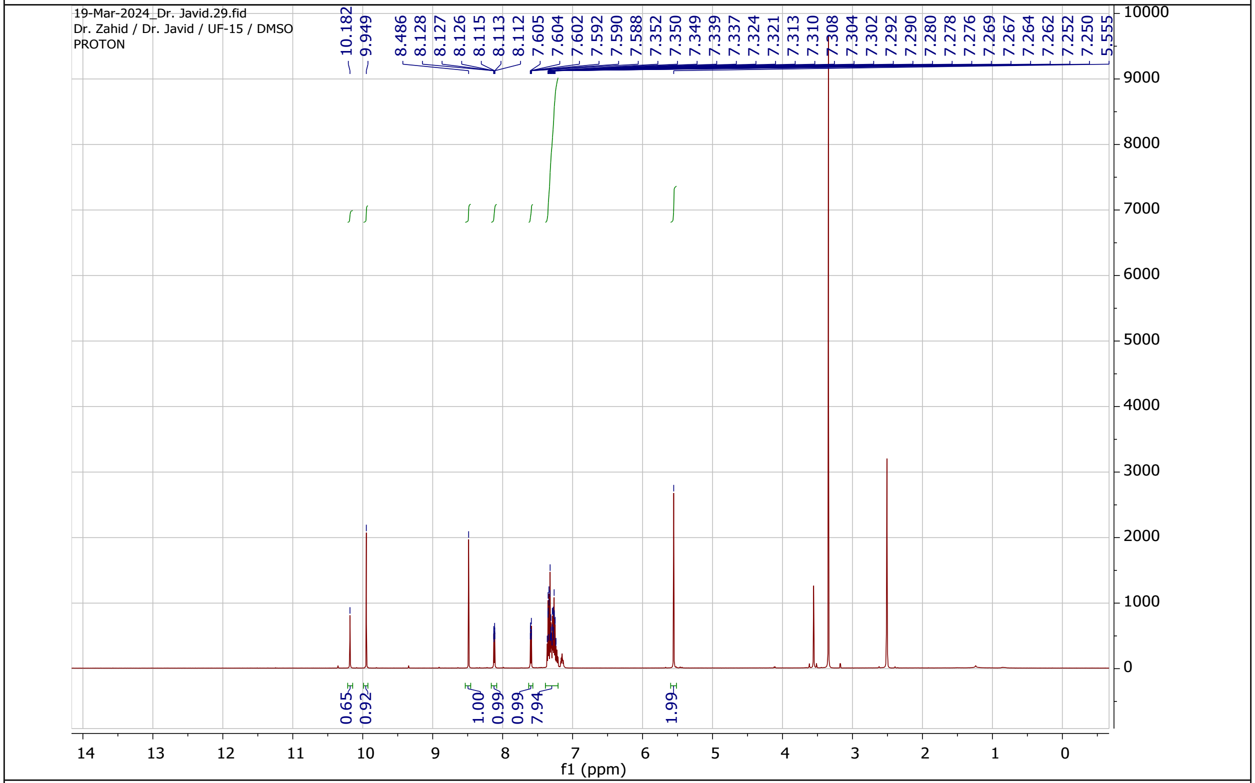

13CNMR of 5s

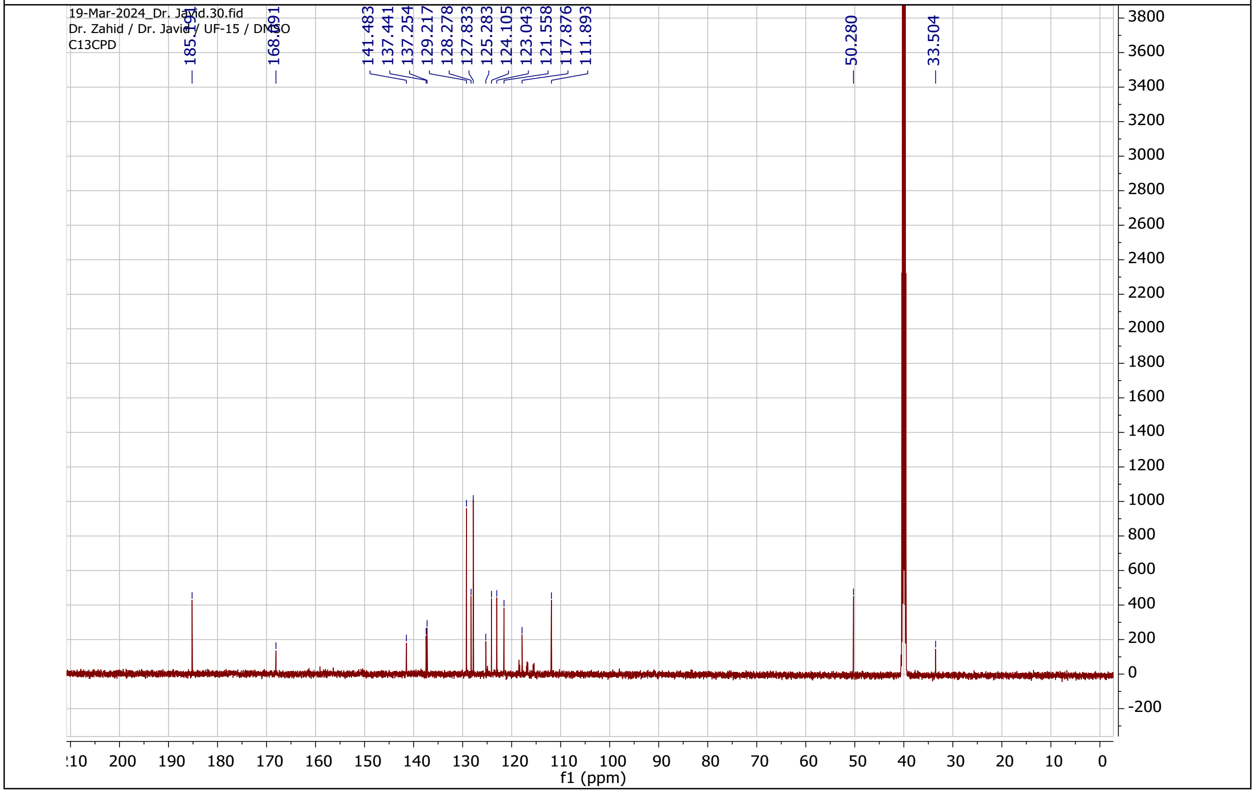

1HNMR of 5t

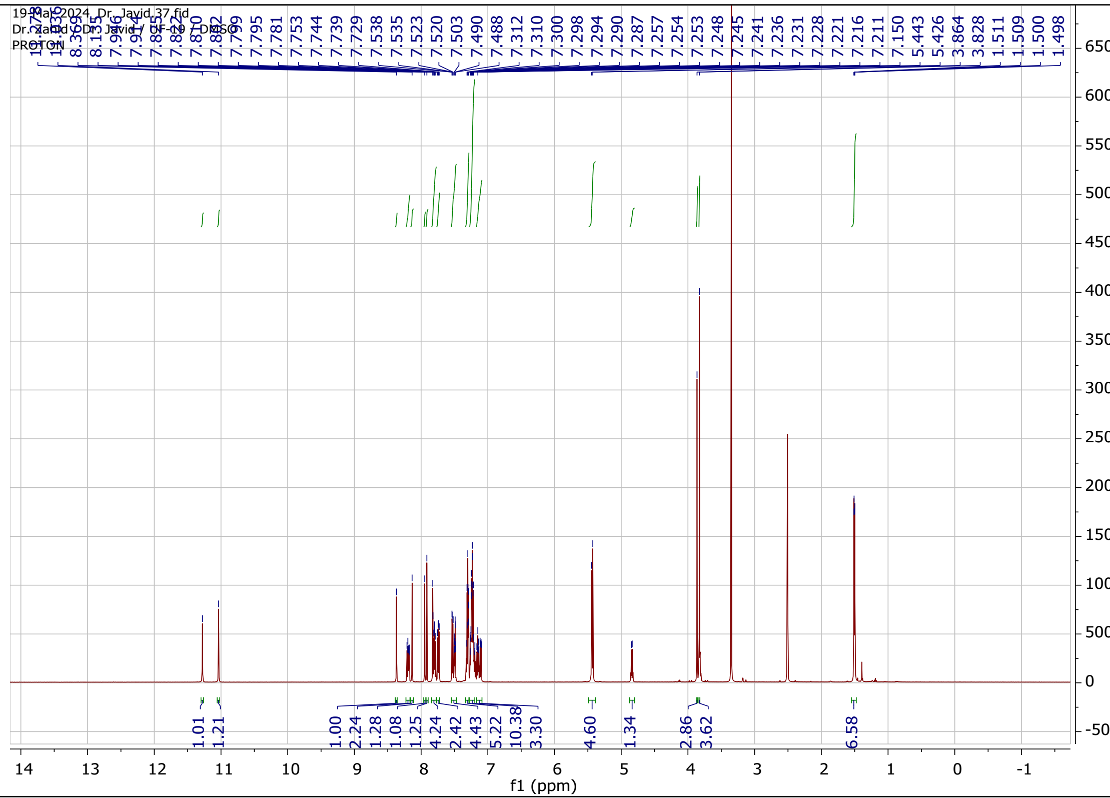

13CNMR of 5t

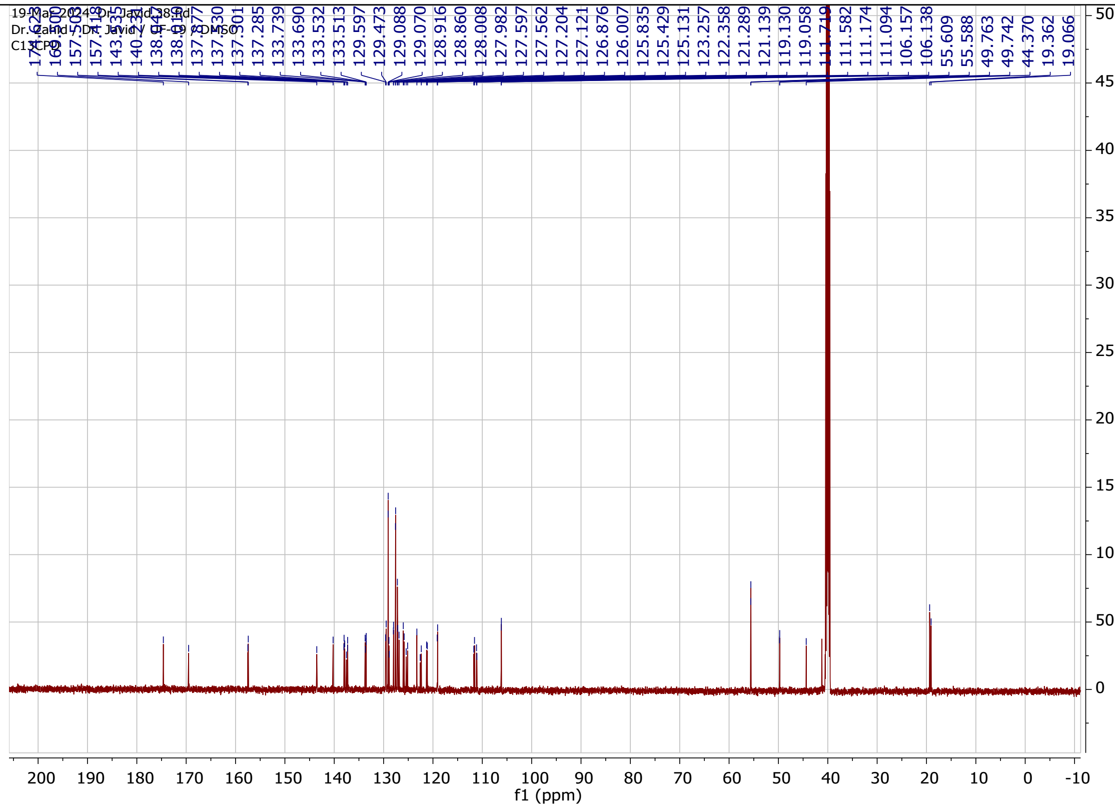

1HNMR of 5u

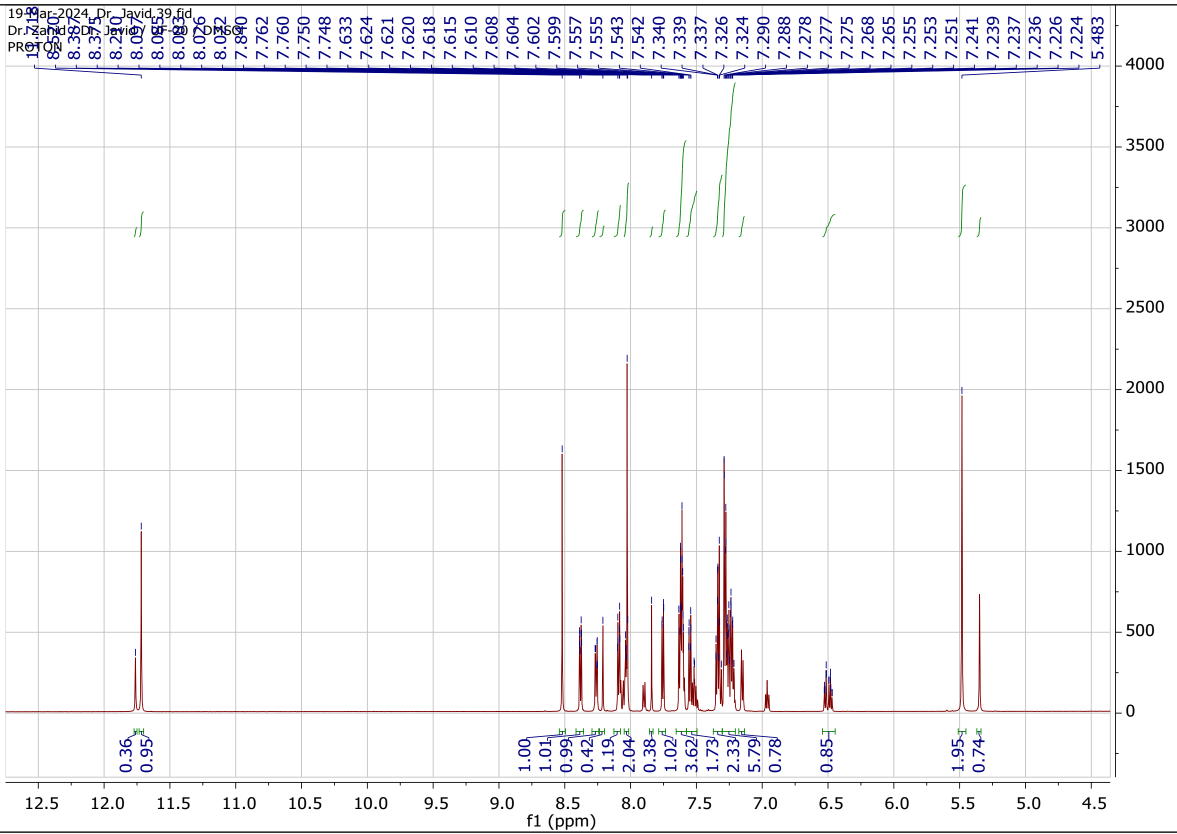

13CNMR of 5u

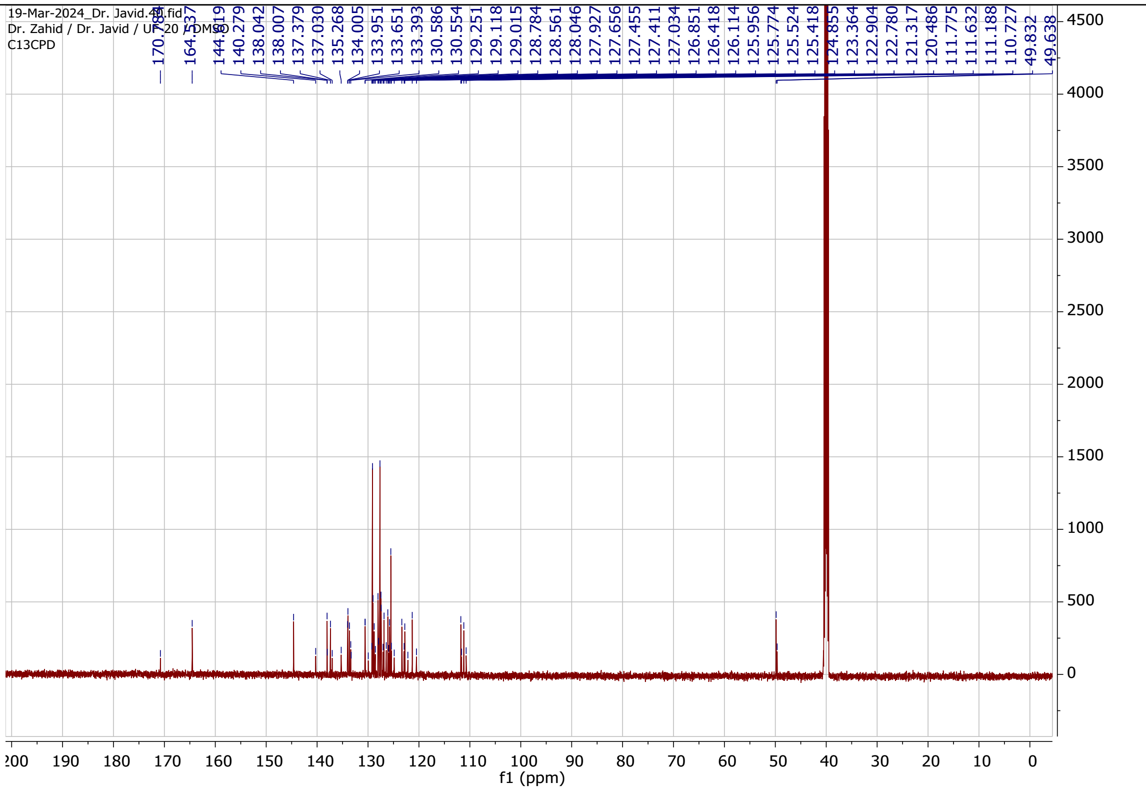

1HNMR of 5v

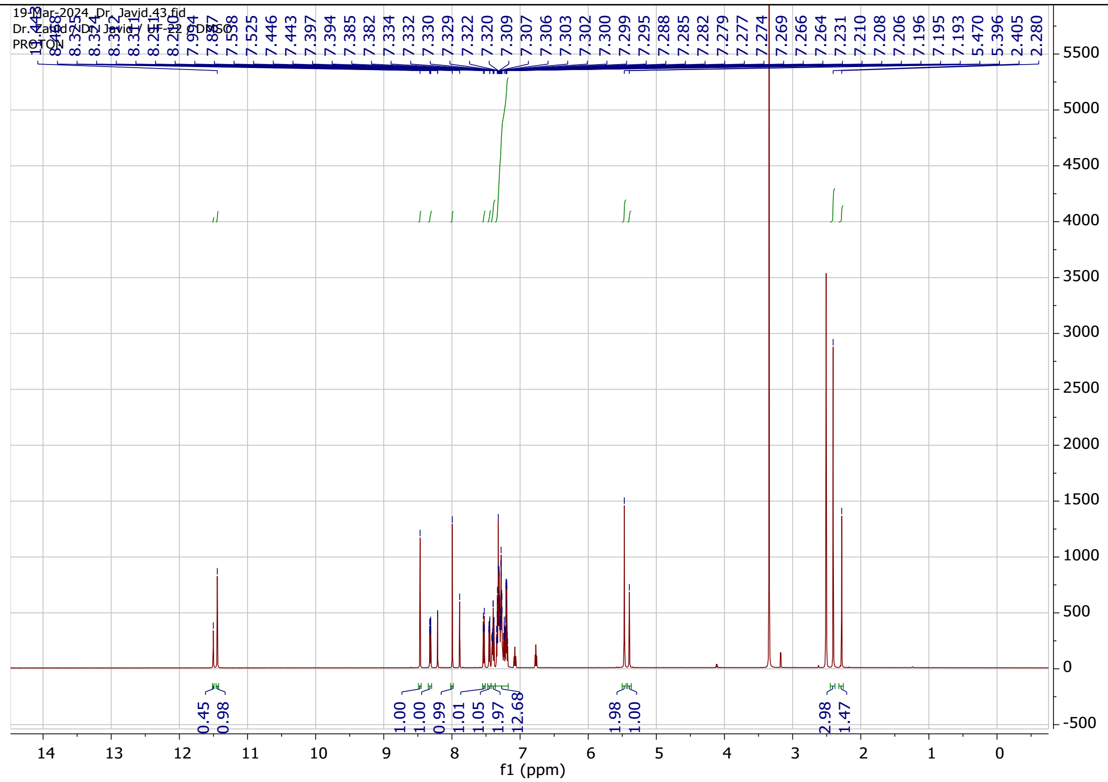

13CNMR of 5v

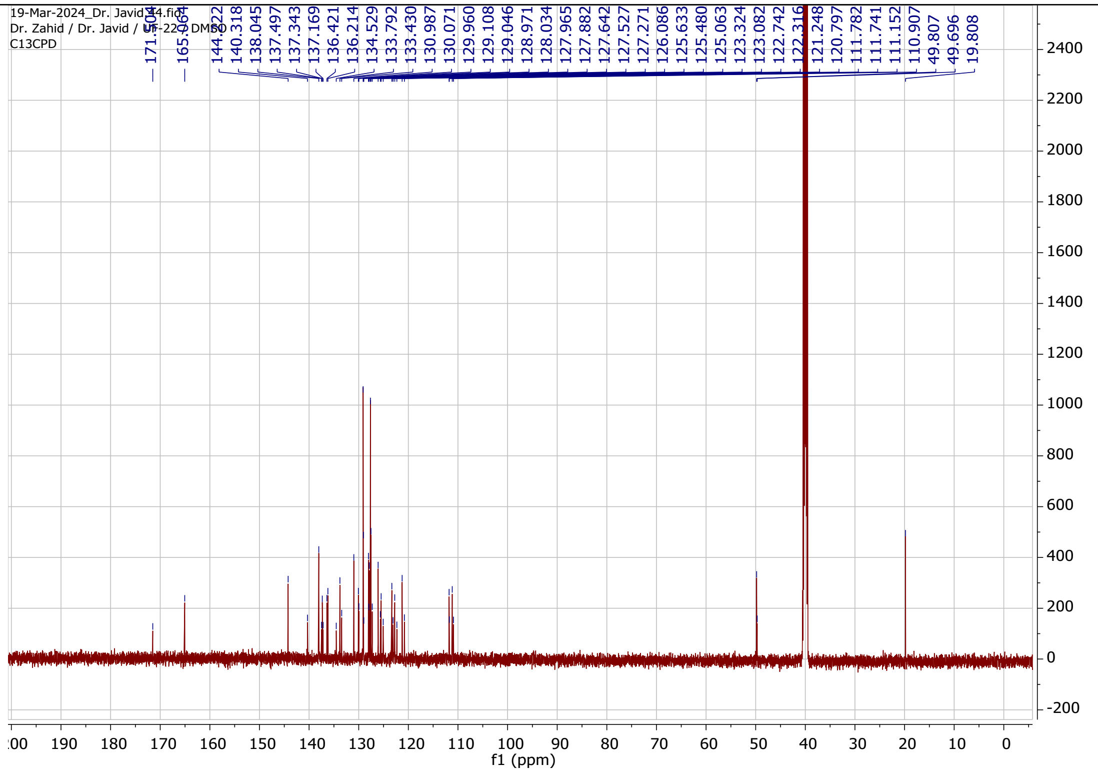

Supplement: RA-015-D5RA02194D-s001 [file RA-015-D5RA02194D-s001.pdf]
